# tmQM-RDF Data Set: A Knowledge Graph Representing Transition Metal Complexes

**DOI:** 10.1021/acs.jcim.6c01281

**Published:** 2026-06-24

**Authors:** Luca Cibinel, Trond Linjordet, Johan Pensar, David Balcells, Riccardo De Bin, Basil Ell

**Affiliations:** † Integreat—Norwegian Centre for Knowledge-driven Machine Learning, 0851 Oslo, Norway; ‡ Department of Mathematics, 6305 University of Oslo, P.O. Box 1053, Blindern, 0316 Oslo, Norway; ∇ Hylleraas Centre of Excellence for Quantum Molecular Sciences, Department of Chemistry, University of Oslo, P.O. Box 1033, Blindern, 0315 Oslo, Norway; § Department of Chemistry, University of Oslo, P.O. Box 1033, Blindern, 0315 Oslo, Norway; ∥ Institute for Chemical Reaction Design and Discovery (WPI-ICReDD), Hokkaido University, Sapporo 001-0021, Japan; ⊥ Center for Cognitive Interaction Technology, 9167 Bielefeld University, 33619 Bielefeld, Germany; # Department of Informatics, 6305 University of Oslo, P.O. Box 1080, Blindern, 0316 Oslo, Norway

## Abstract

Transition metal complexes (TMCs) have wide-ranging practical utility in chemistry, with possible applications that range from catalysis to medicinal chemistry. The study of TMCs and their properties is thus a field rich with potential, one in which machine learning and computational approaches can offer a substantial aid. For this reason, appropriate and accessible data sets, collecting a wide range of information, are required in order to facilitate the effective analysis and investigation of such compounds. This paper contributes to the data modeling effort via the introduction of the *transition metal quantum mechanics RDF* (tmQM-RDF) data set, a knowledge graph constructed using the Resource Description Framework (RDF) vocabulary which collects rich and detailed descriptions of approximately 60k TMCs. These descriptions are both qualitative and quantitative, encompassing the compositional nature of TMCs as well as the entirety of their molecular graphs. The Python package tmqmrdfdata is released together with the knowledge graph to provide a user-friendly interface. An example of the power of the proposed representation is presented, showcasing how the information available in tmQM-RDF can be exploited for TMC manipulation tasks, achieving promising performance even with relatively simple probabilistic models.

## Graphical abstract

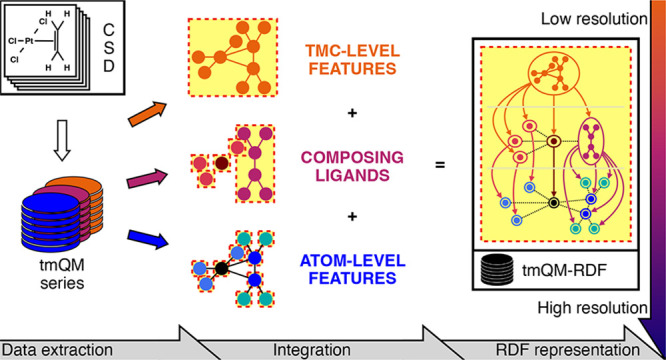

## 1 Introduction

Transition metal complexes (TMCs) are chemical compounds with a widespread range of applications. Fields such as catalysis, medicine, and materials science can gain significant benefits from advancements in our understanding of TMCs.^1,2^ What makes this chemical family so broadly relevant is the possibility of adjusting their properties by appropriate manipulation of the bonds between the transition metal center and the ligands.^3^ At the same time, the involvement of *d* orbitals in these bonds make TMCs more difficult to represent as molecular graphs, compared to other classes of molecules.^4,5^ This is one of the key challenges that arise in the study of TMCs. Another fundamental obstacle is represented by the combinatorial explosion of the number of possible chemical structures, due to the numerous possible assemblies of the central transition metal atom with the surrounding ligands.^3^

In order to tackle and overcome these issues, computational techniques have become increasingly predominant in the last decades. Density functional theory (DFT) is one of the fundamental pillars of modern computational chemistry, as it enables the preliminary screening of compounds of interests on the basis of their electronic properties.^6^ More recently, machine learning (ML) approaches have attracted considerable interest as surrogates for DFT simulations, which can easily become prohibitively expensive in terms of computational costs.^4,7^ ML models, on the other hand, have considerably lower inference times, making them an attractive solution for tasks like property prediction, electronic structure calculation or even structure generation.^8–10^

This new paradigm, however, comes with its own set of caveats. First, popular ML algorithms, like deep neural networks (DNNs), are notorious black boxes, and do not allow for their parameters to be intuitively interpreted.^8,10^ Second, acceptable performances are typically achieved only when an adequately large amount of data is available for the training of the model. This is perhaps one of the most crucial aspects of ML and DNNs, as the quantity, quality, and chemical diversity of the training data can have a dramatic impact on the results.^7,11,12^

Notable efforts have been dedicated, then, to the curation of suitable data sets which can provide a solid foundation for ML training. In the case of TMCs, the most prominent representative of this effort is the tmQM data set series,^5,13–16^ which describes a subset of the TMCs reported in the Cambridge Structural Database (CSD).^17^ Among its many contributions, we primarily highlight the merit of having provided, with tmQM, one of the first large-scale data sets on the geometric and electronic properties of TMCs,^13^ while also tackling the problem of molecular graph representation, in tmQMg,^5^ and that of extensive ligand description, with tmQMg-L.^14^ The intrinsic power and utility of this data set series have been demonstrated by the numerous applications and extensions found in the literature.^4,7,18,19^

Nevertheless, diversity and size are not the only desirable properties of a data set. Accessibility, ease of manipulation, and compatibility with other resources are also important factors that can affect the overall impact of a data set on the research process.^20^ Data integration and machine readability are the main focus of the Semantic Web Standards, centered around the objective of creating a “Web of Data” that is easily accessible, and most importantly interpretable, to both humans and machines.^21^ This kind of methodology prompted the development of a long-standing series of computational tools for chemical research.^20,22,23^ In addition, several chemical data sets and ontologies, i.e., collections of large-scale standardized representations, have been deployed over the years, contributing to the development of computational chemistry under this framework.^24–27^

This work participates in the data representation effort by building on top of the tmQM data set series, specifically on the three data sets tmQM, tmQMg and tmQMg-L. Many of the key challenges in modern data collection, such as chemical variety, ML-readiness, and appropriate chemical representation, are already addressed,^5,13,14^ and we contribute to the existing resources by organizing the content of the three data sets into a single coherent representation that can facilitate data access. Inspired by the aforementioned principles of data availability, readability, and operability, we present the tmQM-RDF data set, an integrated description of a large (approximately 60k entries) and diverse population of TMCs, encoded using the Resource Description Framework (RDF) vocabulary^28^ and its RDF Schema (RDFS) semantic extension.^29,30^

By leveraging the RDF and RDFS vocabulary, tmQM-RDF falls into the category of knowledge graphs (KGs),^31^ where the fundamental units of information are triples of the form $(t_{s},t_{p},t_{o})\in T^{3}$ , where T is a (possibly infinite) set of terms. Each triple represents a subject-predicate-object structure, but indeed it can also be interpreted as a directed labeled edge between two nodes, namely the *subject t*_*s*_ and the *object t*_*o*_, where the label is the *predicate t*_*p*_. In tmQM-RDF, then, knowledge about TMCs is easily accessible using this dual interpretation.

We furthermore contribute an example application of the novel integrated data representation, and define the task of *plausible TMC completion*. By extracting frequent structural motifs from the data set and then estimating their joint distribution using statistical techniques, we show how to quantitatively assess the appropriateness of a candidate TMC structure considered as a completion of an initial molecular scaffold.

The remainder of this paper is organized as follows. Section 2 introduces the vocabulary used to build the tmQM-RDF KG. Section 3 presents a detailed account of how tmQM-RDF is built using the information from the tmQM series. In Section 4 we present and discuss our example on plausible TMC reconstruction powered by the structural analysis of the content of tmQM-RDF. Finally, in Section 6 we summarize our main contributions. Additional content, such as notable subsets of tmQM-RDF, and technical details about the creation of the data set and the example application, can be found in the Supporting Information (SI).

## 2 Methods

The act of converting existing chemical data sets into RDF format is not unprecedented and several notable examples can be found in the literature.^32–34^ The creation of tmQM-RDF falls within this category, as it requires a careful examination of the data contained in the tmQM data set series and the elaboration of an appropriate *terminology component*, or *TBox*.^35^ This section focuses on this aspect of data modeling, by introducing the challenges involved in designing the semantic representation of TMCs and the proposed solution, intended as an RDF vocabulary.

In accordance with the RDF syntax, we use Uniform Resource Identifiers (URIs) to identify entities and resources, blank nodes to state the existence of entities for which it is not necessary to state their precise identity,[Fn fn1] and literals to report quantitative data.

For the sake of brevity, we adopt the common convention of shortening URIs by employing prefix names. An excerpt of the prefixes we use is available in Table 1. For a full list, the reader is referred to the SI. As a rule, the newly introduced prefixes adhere to the following scheme: the first two lowercase letters denote the general topic (the whole **c** o **m** plex, **l** i **g** and-like structures, **a** to **m** ic structures, and **d** ata **s** ets), the following uppercase letter specifies the first subtopic ( **T** MC, **B** onds, metal **C** entre, **L** igand, **S** tructural connectivity, **A** tom). A variable number of lowercase letters can follow, indicating further specification.

**Table 1:** In RDF, Entities Are Represented via URIs, Which Are Often Organized by Namespaces^a^

Prefix	Namespace	Relevant prefixes
rdf	http://www.w3.org/1999/02/22-rdf-syntax-ns#	
rdfs	http://www.w3.org/2000/01/rdf-schema#	
xmls	http://www.w3.org/2001/XMLSchema#	
ms	https://www.integreat.no/research/rdf/tmqm-rdf-dataset/#/misc/	
nm	https://www.integreat.no/research/rdf/tmqm-rdf-dataset/#/numerical/	
*Families of namespaces*		
cm*	https://www.integreat.no/research/rdf/tmqm-rdf-dataset/#/complex/*	cmT cmTp
lg*	https://www.integreat.no/research/rdf/tmqm-rdf-dataset/#/ligand/*	lgB lgC lgCr lgL lgLr lgS
tm*	https://www.integreat.no/research/rdf/tmqm-rdf-dataset/#/atomic/*	tmA tmAr tmB tmBp tmS
ds*	https://www.integreat.no/research/rdf/tmqm-rdf-dataset/#/datasets/*	ds

a These namespaces can employ prefixes as their alias, as a way to shorten URIs. This table summarizes the namespace prefixes used in this work. To improve readability, certain groups of prefixes are not listed entirely, but rather are grouped using the symbol * as a placeholder for a (possibly empty) sequence of characters. For each group, a (non-exhaustive) list of the most important prefixes is reported. See the SI for a complete discussion.

### 2.1 Semantic Integration of the tmQM Series

The vocabulary that we employ to semantically integrate the data sets of the tmQM series must be tailored to the data, in order to appropriately convey the information once the unified representation is assembled. The content of tmQM, tmQMg, and tmQMg-L is then hereby briefly introduced, followed by an explanation of how the data is organized in a single, coherent KG.

#### 2.1.1 tmQM Data Set Series

In this work we consider three of the data sets in the tmQM series,[Fn fn2] namely tmQM,^13^ tmQMg,^5^ and tmQMg-L.^14^ These three data sets are closely related and describe the same population of TMCs, but each one provides a different perspective:

- tmQM describes TMCs primarily at a whole-complex level, providing quantum properties computed at the DFT­(TPSSh-D3BJ/def2-SVP) level of theory, based on geometric data optimized at the GFN2-xTB level. Geometric information, in the form of atomic Cartesian coordinates, are also available.
- tmQMg provides some TMC-level quantum properties as well, but unlike tmQM, this data set focuses on the atomic composition of the TMCs. In particular, two different graphical representations have been developed to analyze TMCs from a graph-theoretical point of view, namely the directed and the undirected natural quantum graph.^5^ Here we only employ the undirected version, as this is the one that most closely relates to the classical molecular graph representation. Calculations of geometries, frequencies, and thermochemistry properties in this data set have been conducted at the PBE-D3BJ/def2-SVP level, whereas single-point energy and natural bond orbital (NBO) calculations have been performed at the PBE0-D3BJ/def2-TZVP level. Notice that this data set only contains complexes with at most 85 atoms, hence this constraint will be transmitted to tmQM-RDF as well.
- tmQMg-L acts as a ligand library in which extensive properties, descriptors, and features belonging to ligands, intended as independent entities, are reported. We shall treat the entries of this data set as abstract reference definitions of species of ligands, in a similar way in which chemical elements in the periodic table define species of atoms.^37^ This data set has been extracted from tmQMg after applying a series of filters, including successful charge/coordination mode computation and absence of 3c2e bonds.^14^ Thzae implications of these filters for tmQM-RDF are discussed in Section 3.2.

#### 2.1.2 Hierarchical Representation

We organized the data using a three-level hierarchical representation, in which each level describes the features of a TMC at a different resolution. Each TMC, i.e., each corresponding subgraph of the resulting KG, will be constructed using this scheme.

The top level is the *complex* level, in which we only consider properties that belong to the entire TMC. Here we derive the data mostly from tmQM, but partly also from tmQMg, as it also contains whole-TMC features. Below this level we find the *ligand* level, which specifies the composition of the TMC in terms of its constituting ligands and the metal center, in the tmQMg-L sense. Here we report only structural information, as numerical features are treated either as pertaining to specific atomic bonds or to the general representation of the entire ligand. In the latter case, these properties are specified elsewhere, in what can be considered the ligand-level equivalent of the periodic table. This level draws information from tmQMg-L, whose entries are compared against the information in tmQMg to correctly locate the ligands within each TMC (see Section 3). Finally, the most detailed level is the *atomic* level, which essentially corresponds to the molecular graph of the TMC, enriched with all the relevant node and edge features, as extracted from tmQMg.

### 2.2 Terminology Component of tmQM-RDF

In order to achieve a functional RDF representation that adheres to the principles of the three-level hierarchical representation, there are several modeling decisions that have to be carefully taken. From a high-level perspective, such choices concern themselves with the structural description of TMCs and their electronic properties. The structure of a TMC, in particular, is captured by the description of each level in the hierarchy, as well as the connections between the levels themselves. The latter is essential in order to attain a coherent integration of data at different resolutions.

An exhaustive documentation of the schema can be found in the SI. A small example of a compliant *assertion component*, or *ABox*, i.e., a set of assertions on actual objects that follows the specification of the TBox,^35^ is shown in Figure 1.

**Figure 1:**
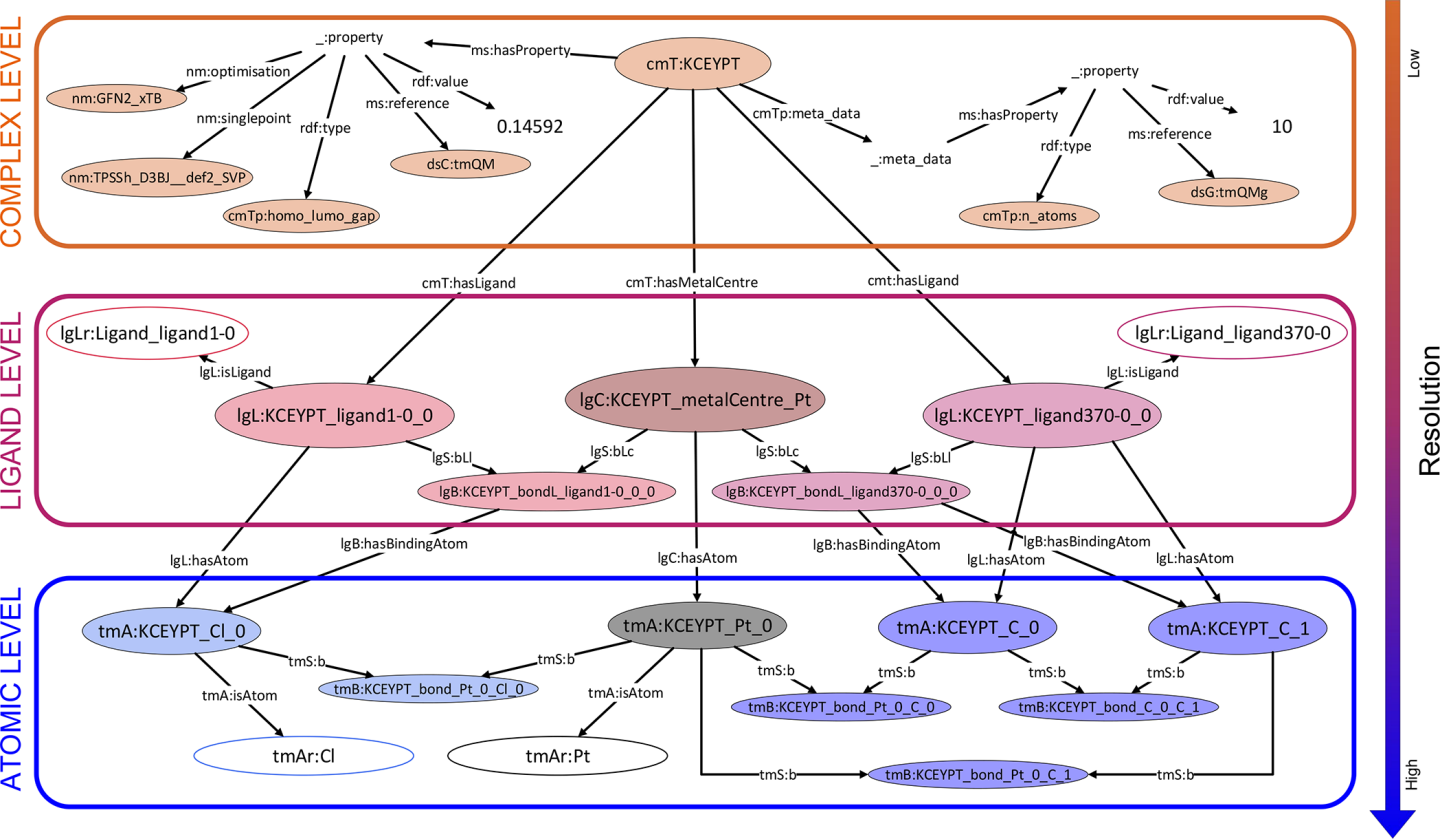
Visual example of an ABox compliant with the TBox introduced in Section 2.2 showcasing how the data from the tmQM series can be represented. Nodes with neither background nor border represent either blank nodes (_:*) or literals (“*”). Nodes with a white background represent RDFS classes. The remaining nodes represent instances of classes. For the sake of readability, not all the features and literal datatypes are represented.

#### 2.2.1 Structure-Related Terminology

The vocabulary has been organized in such a way that information is presented starting from the lowest to the highest resolution, as shown in Figure 2. The fundamental skeleton of a TMC is rooted in an abstract representation of the complex itself, an instance of the RDFS class cmT:TransitionMetalComplex.

**Figure 2:**
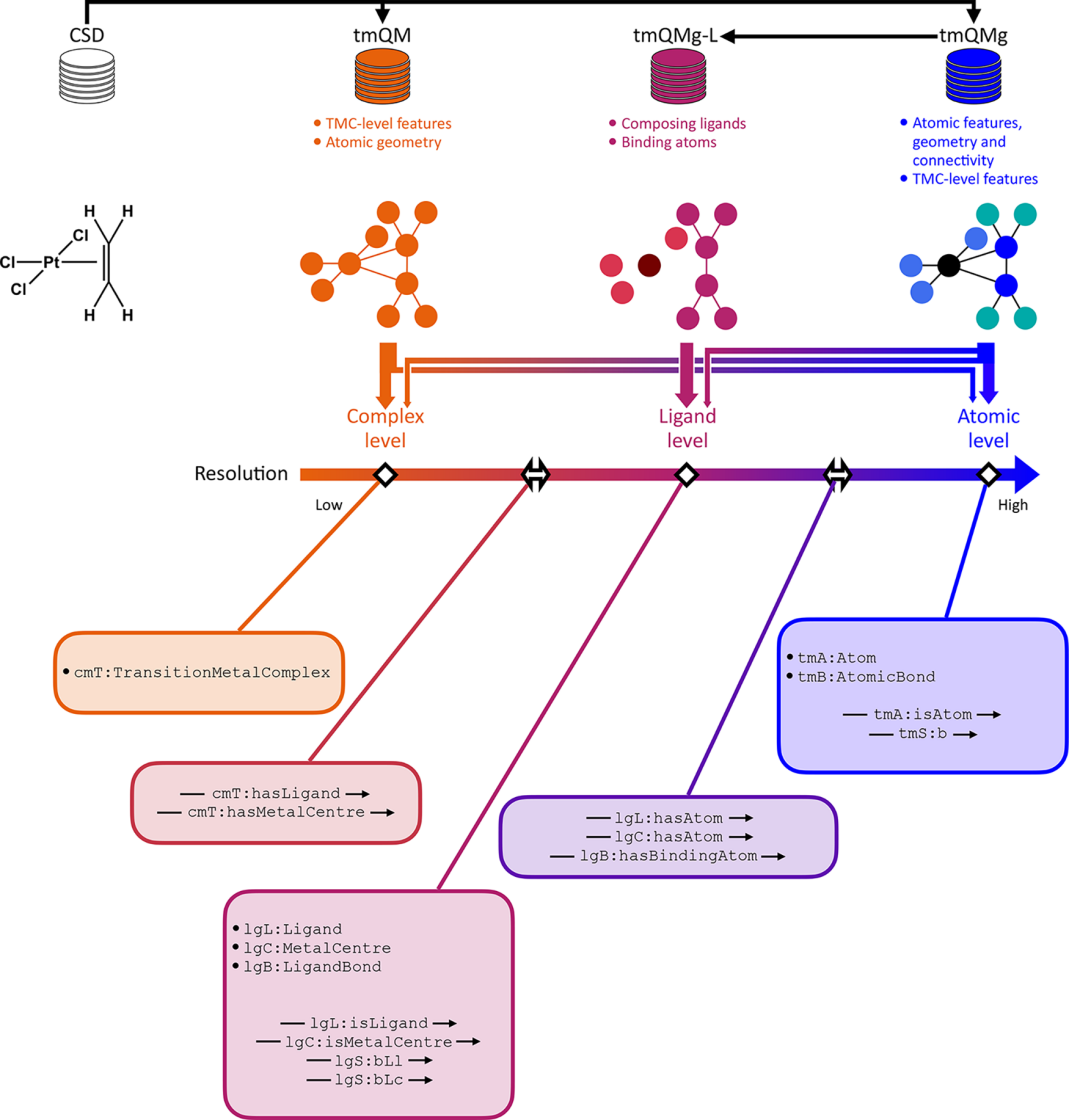
Simplified summary of the structure-related component of the tmQM-RDF TBox. The related RDFS classes and predicates are designed to capture the hierarchical representation from Section 2.1.2 and effectively harmonize the data from tmQM, tmQMg, and tmQMg-L. A more extensive representation, clarifying the full set of relationships among the terms of the TBox, can be found in the SI.

A TMC can then be further decomposed into its ligands and the metal center. This also requires appropriate abstract representations for these structures, leading to the introduction of the classes lgL:Ligand and lgC:MetalCentre. At this point, three additional aspects need to be addressed: the nature of each substructure, their mutual connectivity, and their relationship with the cmT:TransitionMetalComplex instance. Specifying the “nature” of a substructure actually means identifying each structural component (either a ligand or the metal center) as an instance of a certain chemical species, which can either be a ligand species in the sense of tmQMg-L, as explained in Section 2.1.1, or as a chemical element, in the case of the metal center.[Fn fn3] Identification is achieved via the predicates lgL:isLigand and lgC:isMetalCentre, which connect instances to the correct labels. The connectivity between structures, on the other hand, is defined by the set of all the metal center-binding sites of each ligand. Each of these sites is represented using the class lgB:LigandBond. Ligands point to their binding sites using the predicate lgS:bLl. The metal center points to each binding site using the predicate lgS:bLc. Finally, each ligand and the metal center are registered as components of the TMC using the predicates cmT:hasLigand and cmT:hasMetalCentre, which the abstract TMC token uses to point to each ligand/metal center instance.

At the atomic level, the most detailed one, the natural quantum graph of the TMC is encoded. The vocabulary associated with this level follows the same principles employed for the ligand level, but with fewer intricacies. In this case, there exists only one type of entity, i.e., the atoms, represented by the class tmA:Atom. As above, instances of this class are actually representatives of one of the chemical elements found in the periodic table. Facts of this form are expressed using the predicate tmA:isAtom. Connectivity, i.e., atomic bonds, relies on the class tmB:AtomicBond. Each instance represents a bond between two atoms, which state their participation in the bond by pointing to the corresponding tmB:AtomicBond instance using the predicate tmS:b.

The entities in the atomic level are directly related to the ligand level only. Ligands point to their composing atoms through lgL:hasAtom, whereas the metal center does so via lgC:hasAtom. This scheme also applies to the instances of lgB:LigandBond, for which each binding site points to the atoms that take part in it by using the predicate lgB:hasBindingAtom. This mechanism also serves as an implicit definition for the hapticity of a ligand:[Fn fn4] atoms that are part of the same binding site are to be considered as being haptic with respect to each other.

#### 2.2.2 Property-Related Terminology

In principle, every entity in the tmQM-RDF KG can be endowed with properties, either qualitative or quantitative. Regardless of the holder of a property or the nature thereof, all such statements are expressed using some variation of the following scheme.

A property is summarized by an instance of the RDFS class ms:ObservedProperty. This instance will be acted upon by, at least, three more predicates: rdf:type, which states what property is being described, rdf:value, which indicates the actual value of the property, and ms:reference, indicating in which data set of the tmQM series the property was originally reported. A property described in such a way is assigned to an entity through the predicate ms:hasProperty, whose object is the instance of ms:ObservedProperty and the subject is the entity expressing the property.

For some numerical properties, the DFT levels of theory used in their computation are also explicitly available and are reported in the KG. Such information is attached to the relevant properties using the predicates nm:optimization and nm:singlepoint, which indicate the methods used to optimize the molecular geometries and compute their electronic structure properties, respectively.

This is the basic structure used by tmQM-RDF to describe properties. In some cases, this scheme has to be extended to accommodate more complex features, such as lists of Cartesian coordinates or counts of elements within a structure. Precise details about these additional formulations are given in the SI.

## 3 Transition Metal Quantum Mechanics RDF Data Set

A collection of 59, 143 TMCs, represented using the RDF vocabulary developed in Section 2, has been assembled into the *transition metal quantum mechanics RDF (tmQM-RDF) data set*. To facilitate access and data retrieval, the tmQM-RDF knowledge graph is shipped together with a supporting Python package, tmqmrdfdata, which serves both as a download utility and as a user-friendly interface.

As anticipated, tmQM-RDF relies on the information extracted from the tmQM data set series, which has been organized in the three hierarchical levels described above in Section 2. As we want to ensure that tmQM-RDF only contains TMCs for which information can be found in all the data sets of the series, we apply filters to exclude structures that do not meet this requirement.

The fusion of the three parent data sets into tmQM-RDF consists of three main steps, which are further divided into smaller subtasks:

1. tmQMg and tmQMg-L are linked together.
  a. The geometries of the ligand instances in tmQMg-L are matched against the geometries reported in tmQMg, in order to associate each instance with the correct group of atoms.
  b. Using this information, the ligand-level composition of each TMC in tmQMg is computed.
  c. A sanity check is performed, verifying that for each TMC, the ligands extracted from tmQMg-L cover the entire molecular graph. TMCs that fail this check are removed from the data set.[Fn fn5]
2. tmQM and tmQMg are linked together.
  a. The content of tmQM is inspected and indexed according to the viable TMCs identified in the previous step. The TMCs for which it is not possible to retrieve complete information are removed from the data set.[Fn fn6]
  b. The information in tmQM is reorganized in a new structure that mirrors that of tmQMg.
3. tmQM, tmQMg and tmQMg-L are merged into tmQM-RDF.
  a. All the processed information is used to build the RDF representation of the remaining TMCs, using the vocabulary defined in Section 2.1.

### 3.1 Data Set Summary

In total, the tmQM-RDF knowledge graph contains approximately 660 million triples. Of these, only 728 are part of the TBox. The remaining triples form the ABox. The assertions regarding TMCs are formed by around 640 million triples (on average, each TMC is described by 11k triples), those about ligands amount to 14 millions (approximately 436 triples per ligand), whereas the remaining 428 triples describe metal centers and other elements.

As for the chemical expressivity of tmQM-RDf, a concise summary is provided in Figure 3. Figure 3, panel (a) shows a bar plot of the size of the subpopulations of tmQM-RDF identified by the metal center, and thus it offers an initial account of the diversity of the complexes represented in the data set. In particular, it shows the wide selection of transition metals that can be encountered, while also emphasizing that this representation is not uniform.

**Figure 3:**
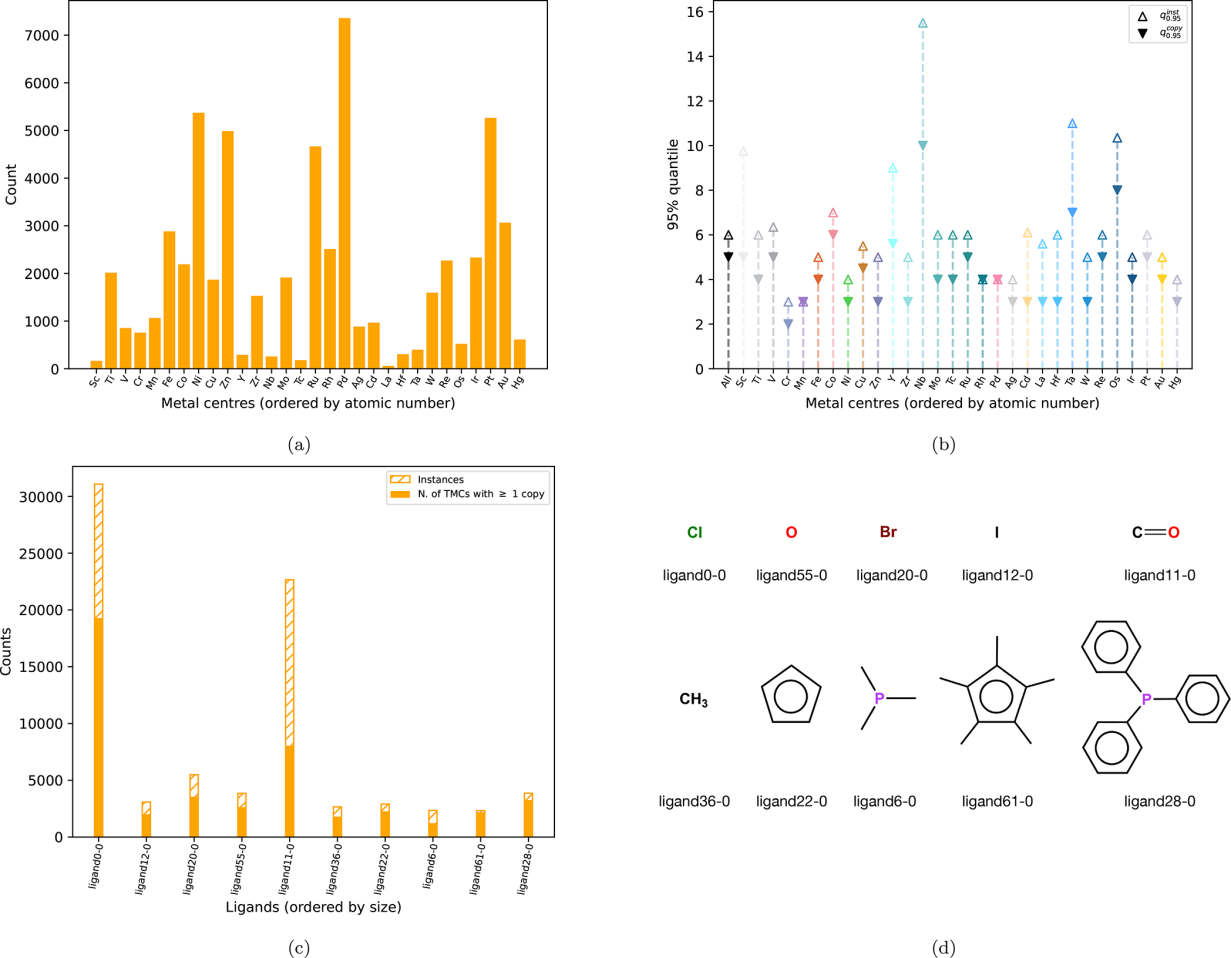
(a) Bar plot of the counts of the appearances of the metal centers found in tmQM-RDF. (b) 95*%* quantiles of the empirical distributions of the count of single ligand instances (*q*_0.95_^inst^) and of the count of TMCs that contain at least one copy of each ligand (*q*_0.95_^copy^), in each subpopulation of TMCs identified by the metal center. Vertical lines are added for readability. (c) Bar plot of the counts of the appearances of the 10 most frequent ligands in tmQM-RDF. (d) Visual representation of the 10 most frequent ligands in tmQM-RDF.

As per the abundance and the variety of the ligands that bind to the metal centers, there are currently 27,905 different ligands in tmQM-RDF, and the average number of ligands per TMC is 3.24 (SD = 1.16). It is also interesting to investigate the frequency of appearance of each ligand, as adequate representativeness is essential for downstream tasks that depend on data to extract realistic correlations between different properties, substituents, and behaviors (e.g., generative tasks). With this regard, Figure 3, panel (b) shows the 95*%* quantile of the empirical distributions of two metrics representing two levels of ligand (absolute) frequency: the total count of occurrences of each ligand (*q*_0.95_^inst^) and the number of TMCs that possess at least one copy of each ligand (*q*_0.95_^copy^). These measurements are taken both for the entire data set and for each metal center-specific subpopulation. Both *q*_0.95_^inst^ and *q*_0.95_^copy^ have been designed to capture information availability with regards to ligands. Low values (*q*_0.95_^copy^ = 5 and *q*_0.95_^inst^ = 6 for the entire data set) indicate that most ligands appear only a handful of times. Specifically, *q*_0.95_^inst^ = 6 means that at least 95% of the ligands appear no more than 6 times in total, whereas *q*_0.95_^copy^ = 5 means that at least 95% of ligands appear, each, in at most 5 different TMCs. When compared to the size of tmQM-RDF, these numbers point to a strongly sparse distribution of ligands.

We ultimately consider the 10 most frequent ligands, in the *q*_0.95_^inst^ sense, as a simple means of visually representing the most typical structures found within the data set. We show a plot of the counts of these ligands, in the same two modalities introduced above, and their graphical representations in Figure 3, panels (c) and (d), respectively.

### 3.2 Summary of Excluded TMCs

The sanity checks put in place during the creation of tmQM-RDF lead to the exclusion of 14, 405 complexes, which amount to approximately 21*%* of the available TMC population in tmQMg. As explained above, a necessary condition for a complex to be included in tmQM-RDF is that each subgraph of its natural quantum graph corresponding to a ligand is registered in tmQMg-L as an instance of a ligand species. If this check fails for any of the subgraphs, then the entire complex is excluded. The only other possible cause of exclusion is the absence of complete data regarding the TMC in tmQM, but such a scenario has never been observed during runtime. It follows, then, that any restriction to the final tmQM-RDF population is inherited entirely from tmQMg-L.

The extraction of tmQMg-L required the computation of charge and coordination mode for each ligand. Such operation has a reported success rate of 92%.^14^ Additionally, ligands for which 3-center (3C) bonds are detected or which are part of TMCs whose quantum graph is not connected are excluded as well.[Fn fn7]

These conditions, specifically the 3C exclusion rule, can potentially reduce the overall diversity. We conducted a comparative analysis of the TMC population in tmQMg, tmQM-RDF, and the difference between the two data sets. Figure 4 reports the results with regard to the empirical frequencies of the available chemical elements, the complex sizes (in number of atoms), and a comprehensive summary of the different whole-TMC electronic properties. The three populations appear quantitatively and qualitatively comparable, suggesting that no extreme distortion is introduced with respect to tmQMg, apart from those already present in tmQMg-L.

**Figure 4:**
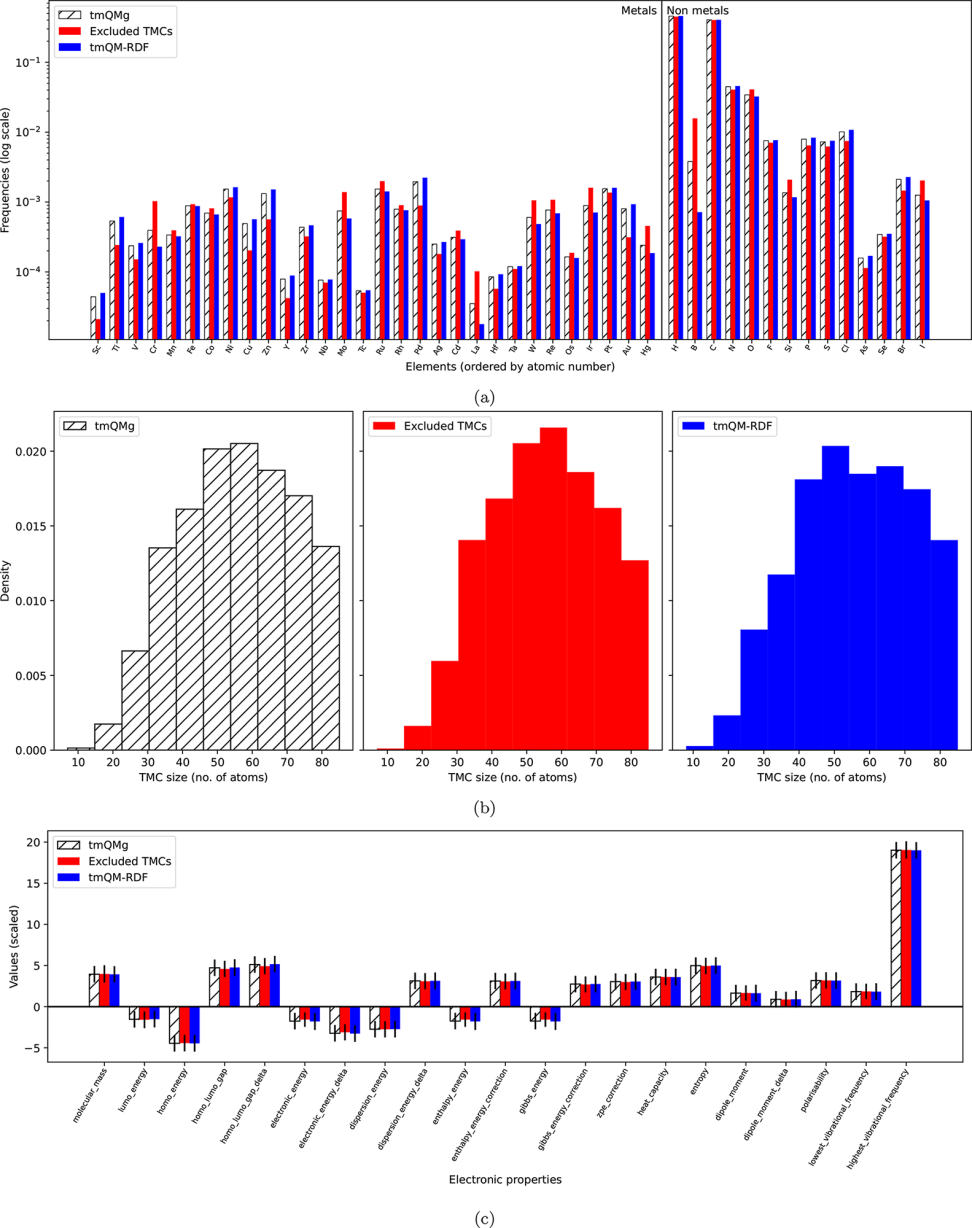
Comparative study of the chemical features found in tmQMg, tmQM-RDF, and the TMCs excluded from tmQM-RDF. (a) Bar plot of the frequencies of the elements found in tmQM-RDF, divided between metals and nonmetals. (b) Histogram of the TMC sizes, in number of atoms. (c) Bar plot of the whole-TMC electronic properties, scaled so that the distributions of the properties of tmQMg have unit variance.

## 4 Experiments: Plausible TMC Reconstruction

In this section we propose an illustrative application of the tmQM-RDF data set, by considering a simple TMC reconstruction and manipulation task, which we call *plausible TMC completion*. Given an incomplete molecular scaffold (i.e., a TMC with a missing ligand) and an appropriately ranked set of candidate completions into full TMCs, we shall refer to the top–*k* highest scoring reconstructions as *plausible*,[Fn fn8] for an appropriate integer value of *k*. This definition is reminiscent of the concept of most likely sentence completion in LLM research^38,39^ and also of the methodological framework of the LigandDiff generative model for scaffold completion.^40^

Conceptually, in this experiment we consider a population G of TMCs (in practice, either one of the two selections introduced below in Section 4.1) sampled from the set of TMCs described within tmQM-RDF. The data set G is then split into two parts: a training set $G_{\mathrm{train}}$ , used to acquire relevant structural and chemical information that can be used to assign a score to a candidate TMC, and a test set $G_{\mathrm{test}}$ , which constitutes the population of target TMCs that have to be reconstructed starting from molecular scaffolds.

The former is used to implement the training phase of our experiment, the latter for the test phase. Additional information on our approach, including precise definitions and formulations, can be found in the SI.

### 4.1 Experimental Data Sets

With the aim of facilitating the computational experiments, we select two smaller data sets sampled from the general population described within tmQM-RDF, whose distribution of chemical composition and structure does not deviate excessively from that of the parent data set. Both data sets consist of 1300 TMCs, split into training (1000 TMCs) and test (300 TMCs) partitions. In order to cover a wide experimental scope, the two subpopulations consider transition metal centers belonging to groups from separated regions of the periodic table. One data set considers an early region (Cr, Mo, W, *earlyTM* data set selection), whereas the other considers a late region (Ni, Pd, Pt, *lateTM* data set selection). Summaries of the two data sets and additional information about the sampling procedure can be found in the SI.

### 4.2 Training Phase

The ultimate goal of the training phase is to construct a real-valued score function f:G→R that can estimate the degree to which it is plausible that a given TMC was sampled from the population of interest. To achieve this, we proceed as follows.

1. From $G_{\mathrm{train}}$ , a set of frequent (basic graph) patterns, in the SPARQL sense,^41–43^ is extracted. These are meant to capture recurrent substructures within the TMC population, with regards to the modalities through which ligands attach to the metal center atom, i.e., ligand identities, denticity/hapticity order and binding atoms.
2. Using a similarity-based agglomerative clustering algorithm, the set of mined patterns is partitioned. In this way, a set of families of typical substructures can be determined. The similarity between two patterns is evaluated as the cosine similarity between binary vectors, each representing one of the two patterns. The entries of these vectors corresponds to the TMCs in $G_{\mathrm{train}}$ and evaluate to 1 if and only if the associated TMC is matched by the pattern, and 0 otherwise.
3. A (binary) feature vector ***Y***_*G*_ is now created for each TMC $G\in G_{\mathrm{train}}$ . Each feature corresponds to one of the families identified in the previous point and will evaluate to 1 if and only if that family is expressed within the TMC (i.e., there exists a pattern in the family that matches the TMC). The feature will evaluate to 0 otherwise.
4. The joint distribution of the features is estimated.

This last step is the most crucial of the training phase. If G∈G is a TMC, ***Y***_*G*_ is the corresponding binary feature vector introduced in step 3 and $P(Y_{G}|G_{\mathrm{train}})$ is its joint distribution, then the score of *G* can be defined as

$$f(G):=\log\;P(Y_{G}|G_{\mathrm{train}}).$$ 1

If the joint probability distribution of the features is available, and the features correctly represent the fundamental structural modalities underneath the data set, then highly representative TMCs will generate feature configurations that possess a high probability. Obviously, this distribution is not known and the high dimensionality of the feature space makes estimation generally complicated.^44^ For this reason, we model the distribution using the Bayesian Network (BN) framework^45,46^ and estimate it using the training data. BNs are specifically designed to deal with correlated, high-dimensional features, hence they constitute a suitable solution to the challenges at hand.

A graphical summary of the training phase is depicted in Figure 5.

**Figure 5:**
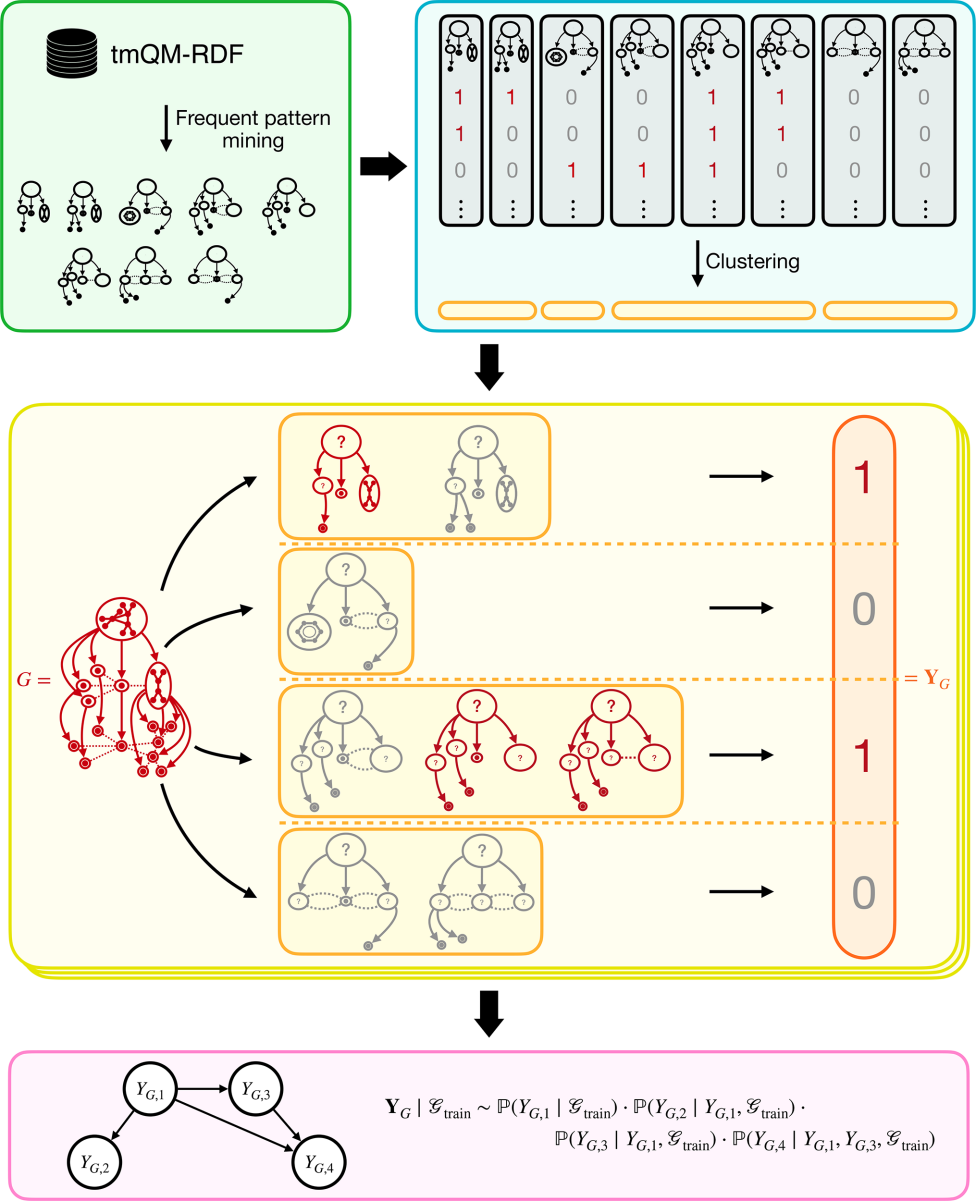
Graphical summary of the training phase of the proposed experiment. First, frequent patterns are mined from tmQM-RDF (top-left panel). Then, the mined patterns are clustered using feature vector representations built by checking the presence/absence of matches of each pattern against the training TMCs (top-right panel). A new feature vector is defined for each TMC in the considered population, where each feature corresponds to one of the previously identified clusters and evaluates to 1 if there exists at least one matching pattern in that cluster, and to 0 otherwise (central panel). Finally, the joint distribution of the new features is estimated using a Bayesian Network (bottom panel).

### 4.3 Test Phase

The test phase is meant to evaluate the goodness of the estimated score function *f* as a tool for TMC manipulation. In particular, under the assumption that the TMCs in G , and therefore in $G_{\mathrm{test}}$ , are themselves representative, we expect that a suitable *f* should be able to adequately reconstruct the elements of G when new transition metal complexes are created by attaching one ligand to an incomplete molecular scaffold. For ease of exposition, we consider here a single test TMC $G\in G_{\mathrm{test}}$ , but the procedure below will be repeated identically for each TMC in the test set.

1. The set *L*_train_ of all the ligands that appear in $G_{\mathrm{train}}$ is computed.
2. A molecular scaffold is created, by removing from *G*, at random, one of its ligand. The ligand to remove is chosen among the ligands of *G* that also appear in *L*_train_.
3. A family of candidate reconstructions is obtained through the completion of the molecular scaffold using, one by one, the ligands from different subsets of *L*_train_. These subsets are determined by applying different constraints on the candidate ligands. In this work we consider the following filters:
  - no constraints;
  - same hapticity and denticity orders as the removed ligand;[Fn fn9];
  - same charge as the removed ligand.
  Notice that, by removing from *G* a ligand from *L*_train_, we ensure that *G*, the original TMC, also appears among the considered reconstructions.
4. Each reconstruction is scored via *f* and the set of reconstructions is ranked accordingly.

A graphical summary is shown in Figure 6.

**Figure 6:**
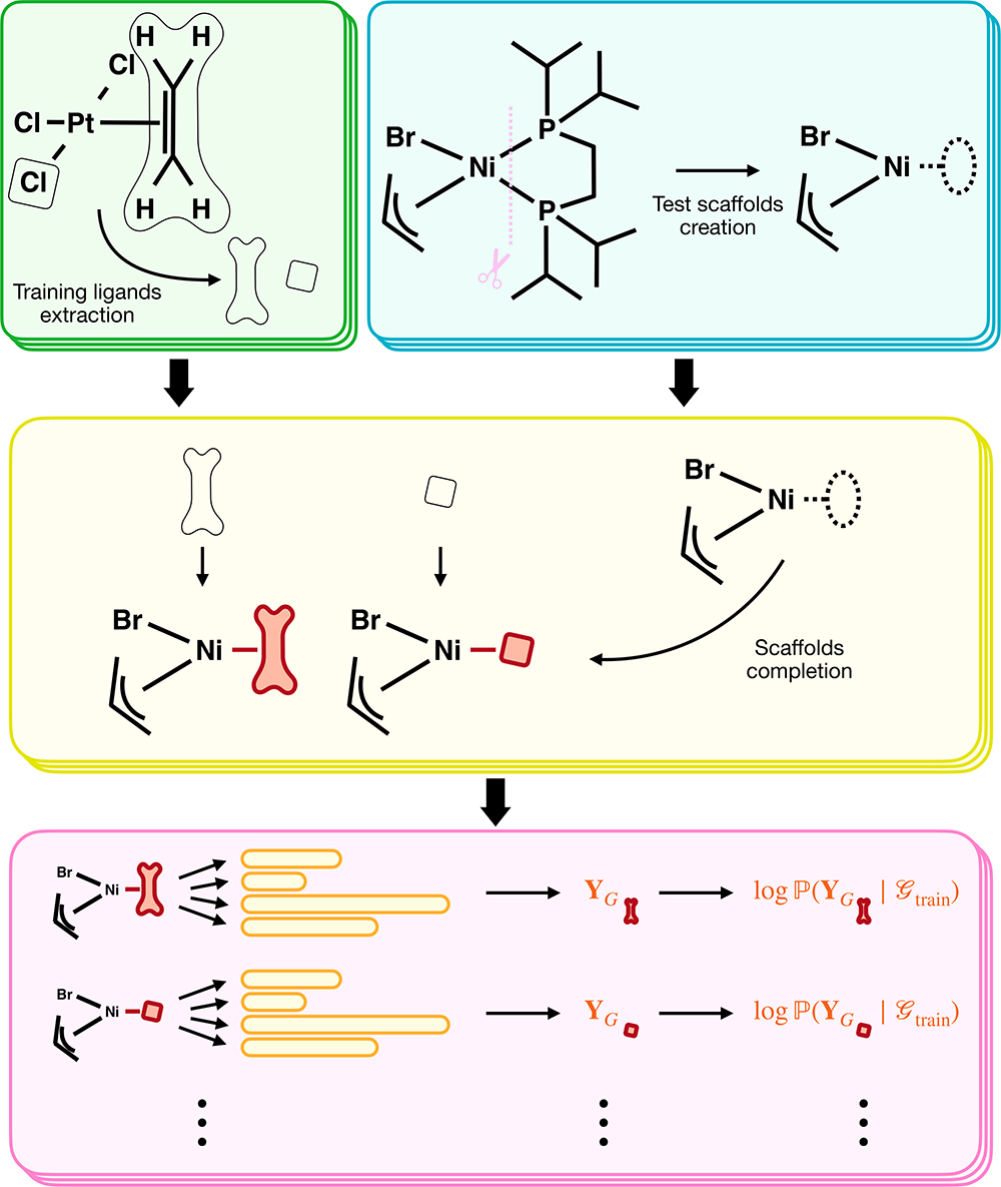
Graphical summary of the test phase of the proposed experiment. First, the set *L*_train_ of all the ligands encountered in the *training* set is computed (top-left panel). Then, for each TMC in the *test* set, a molecular scaffold is created by randomly removing one of the ligands (top-right panel). For each of these scaffolds, a family of possible reconstructions is created by considering all the possible substitutions of the missing ligand with a ligand from *L*_train_ (central panel). Finally, each family is internally ranked according to the decreasing log-probability of the completions’s features (bottom panel).

Being this experiment focused on TMC reconstruction, a natural way to interpret the resulting ranked family of reconstructions is to look for the original TMC among the plausible reconstructions. An equally natural metric to consider, then, is the top–*k* accuracy,^47,48^ which can be interpreted as the fraction of test TMCs that are deemed to be plausible (recall the definition of plausible TMC completion given at the beginning of the section). We focus here on the thresholds *k* ∈{1, 10}.

### 4.4 Results and Discussion

In Table 2 we summarize the results of the experiment. For each of the two data set selections introduced in Section 4.1, we report the number of entries in the structural feature vector ***Y***_*G*_, together with the total number of frequent patterns that have been mined and later clustered into the resulting families of substructures. In addition, we report the measured values of top–*k* accuracy,[Fn fn10] under each constraint, for *k* ∈{1, 10}.

**Table 2:** Number of Features Used, Average Number of Candidate Reconstructions per Scaffold, and Top–*k* Accuracy Measured during the TMC Completion Task for the Two Dataset Selections^a^

	No. of features	Filter								
		*No filter*			*Hapticity/denticity order*			*Charge*		
			Top–*k* accuracy			Top–*k* accuracy			Top–*k* accuracy	
Data set selection	Clustered (mined)	No. of candidates	*k* = 1	*k* = 10	No. of candidates	*k* = 1	*k* = 10	No. of candidates	*k* = 1	*k* = 10
*earlyTM*	455 (1994)	828	0.189	0.796	337.0 (SD = 121.5)	0.268	0.940	321.2 (SD = 116.6)	0.270	0.862
			[0.462]	[0.740]		[0.540]	[0.828]		[0.632]	[0.818]
*lateTM*	518 (3710)	325	0.297	0.709	133.1 (SD = 41.1)	0.354	0.860	141.75 (SD = 26.8)	0.327	0.853
			[0.153]	[0.381]		[0.200]	[0.531]		[0.205]	[0.534]

a With respect to the number of available features, the quantity in parentheses indicate the number of mined frequent patterns which have been clustered into the final families of typical substructures. The scores achieved by the baseline are reported in square brackets beneath the values of the BN-based method.

We compare our proposed scoring method against a simple frequency-based baseline, which scores each candidate reconstruction according to the relative frequency of the chosen ligand, conditionally on the metal center of the scaffold and the filter being employed.

Based on the results in Table 2, we focus the discussion below on two key takeaways. We provide additional considerations (and a more extensive set of results) in the SI.

First, our approach is capable of achieving more than 70–90*%* accuracy already for *k* = 10 for both data sets. Considering that there are hundreds of possible reconstructions in each scenario for each incomplete TMC, as reported in Table 2, this indicates that our approach is able to recognize the test TMCs as plausible members of the TMC population from which $G_{\mathrm{train}}$ was sampled. Around this threshold, moreover, our scoring function outperforms the frequency-based baseline, suggesting that the mined structural features and their BN-estimated joint distribution are better at capturing the binding behavior of less frequent ligands. Most importantly, this successfully validates the usefulness of the integrated representation offered by the tmQM-RDF knowledge graph.

Second, these results highlight two different, but in some sense dual, phenomena taking place in the two considered data sets. In the case of the *lateTM* data set, despite having access to a ∼2 times larger number of patterns compared to *earlyTM* (3710 versus 1994), our method performs slightly worse on *lateTM*. One possible explanation is that there truly is a small set of structures which alone accounts for most of the structural variability in tmQM-RDF. The high number of patterns found when analyzing *lateTM* could then be an indication of overfitting. In other words, the mined patterns may be lacking in quality, or failing to capture a sufficiently diverse set of structures. This could also explain why such a large number of frequent patterns gave rise to a number of families which is comparable to that found in *earlyTM*. Nonetheless, it is equally interesting to notice that our scoring function is more accurate than the baseline at *k* = 1 only in the *lateTM* case. A quick investigation regarding the composition of the complexes in the two data set reveals that the ligand distribution in the *earlyTM* is more skewed than that of the *lateTM* (see Figure 7). This means that, in *earlyTM*, rare ligands are actually rarer than in the *lateTM* selection, making it harder to acquire information on them. So, while in the *lateTM* the pattern mining procedure appears to be prone to overfitting, in *earlyTM* we observe an apparent lack of statistical signal. This stresses the importance of an accurate calibration of pattern mining.

**Figure 7:**
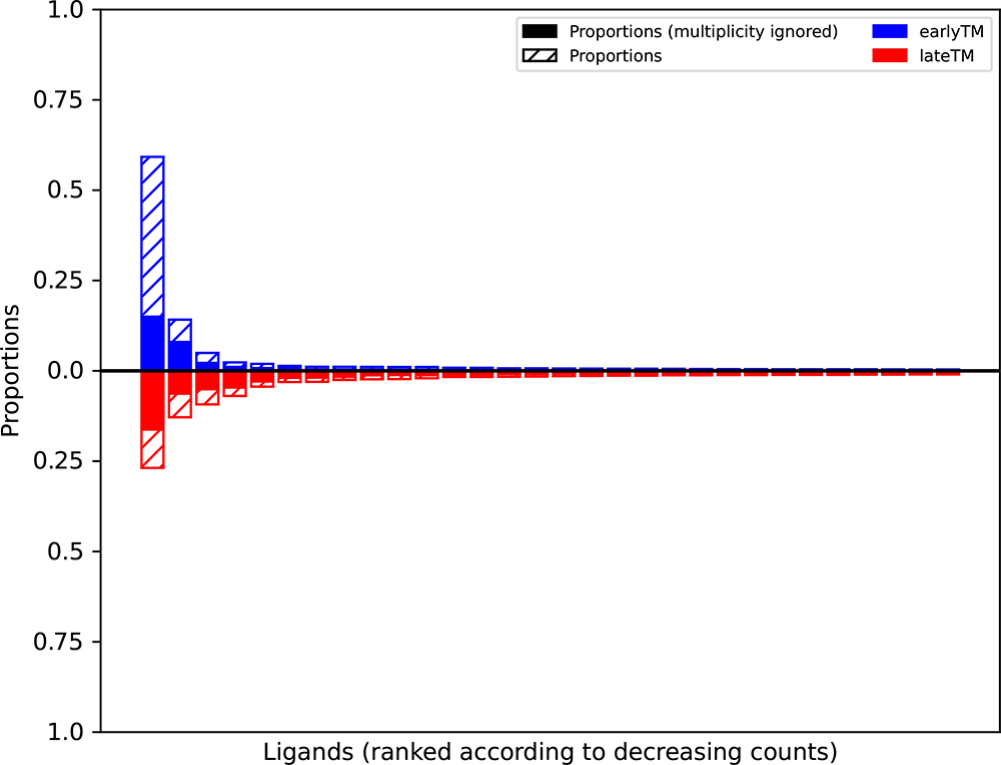
Comparison of the (normalized) counts of the 30 most frequent ligands in the *earlyTM* and *lateTM* selections, ranked according to their decreasing counts. The plot shows that low-ranking ligands in *earlyTM* are rarer than the corresponding ligands in *lateTM*.

#### 4.4.1 Performance with Rare Ligands

We expand our analysis by restricting the test set to only those scaffolds whose original ligand appeared no more than 5 times in the training set. The performances of our methods in this specific scenario, compared against those of the baseline, are illustrated in Figure 8.

**Figure 8:**
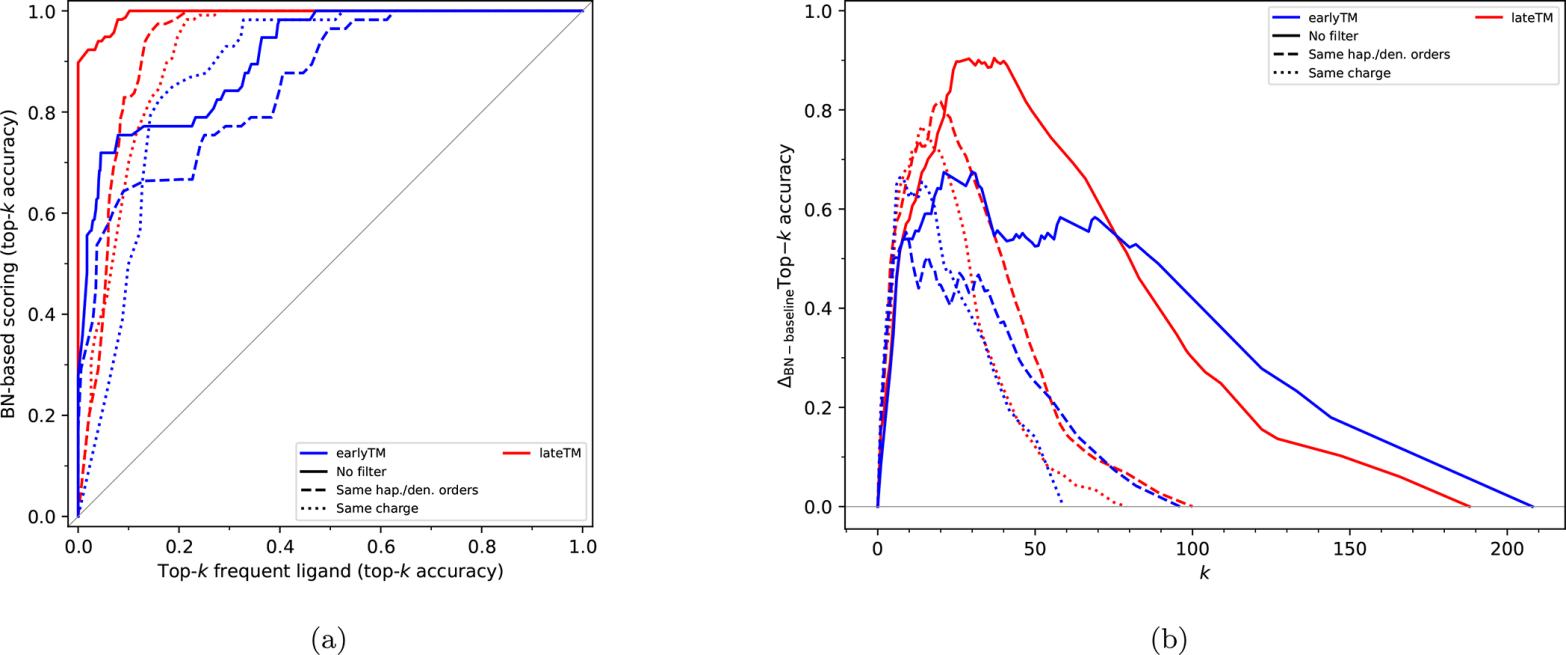
Results of the experiments when the test set is restricted to only those scaffold whose original ligand appeared more than 5 times in the training set. (a) Plot of the top–*k* accuracy achieved by the BN-scoring function against that of the baseline. The region of space above the diagonal indicates improvement over the baseline. (b) Plot of the difference in achieved top–*k* accuracy between the BN-based scoring function and the baseline, against the values of *k*. Positive values indicate improvement over the baseline.

When such a restriction is applied, our scoring function consistently outperforms the baseline, signifying that the structural patterns extracted from tmQM-RDF is capable of capturing at least part of the chemical information about relatively rare ligands, despite their extreme sparsity.

## 5 Case Studies

We now apply the experimental methodology of Section 4 to two case studies, namely the TMCs CAKRAH (from the *earlyTM* selection) and ILUYOD (from the *lateTM*). We are particularly interested in exploring possible modifications of both TMCs with the aid of our RDF-informed statistical model. In these modifications, each ligand is replaced by another drawn from a set of ligands extracted with one of the following filters:

1. **Keeping charge.** The replacement ligand charge must match that of the original ligand.
2. **Keeping hapticity.** For nondentic, haptic ligands with a single η^*n*^ moiety, the replacement ligand should also be nondentic and with a single η^*m*^ moiety of the same hapticity order; that is, *n* = *m*.
3. **Keeping denticity.** For nonhaptic, κ^*n*^ dentic ligands, the replacement ligand should also be nonhaptic and κ^*m*^ dentic, with the same denticity order; that is, *n* = *m*.
4. **No constraint.** No filtering is applied on charge, hapticity, or denticity.

This time, we will not only consider the most probable replacements, but also the *least* probable ones. Compounds that are deemed to be anomalous with respect to the reference population (in our case $G_{\mathrm{train}}$ ) are not necessarily invalid or irrelevant, but could also be interesting novelties not yet examined and whose motifs are, consequently, unknown to the learned scoring function.[Fn fn11]

As an illustrative example, in the CAKRAH TMC (Figure 9) the most probable replacements of the η^3^ anionic allyl ligand were either, when keeping the charge, the *Cp* ring, a ligand commonly found in the early TMCs, or, when keeping hapticity, a similar allyl ligand with *tert*-butyl substituents in the terminal positions. Without constraints, the least probable replacement was a bidentate phosphine ligand, uncommon in early TMC chemistry and yielding an unusually large, eight-membered metallacycle.

**Figure 9:**
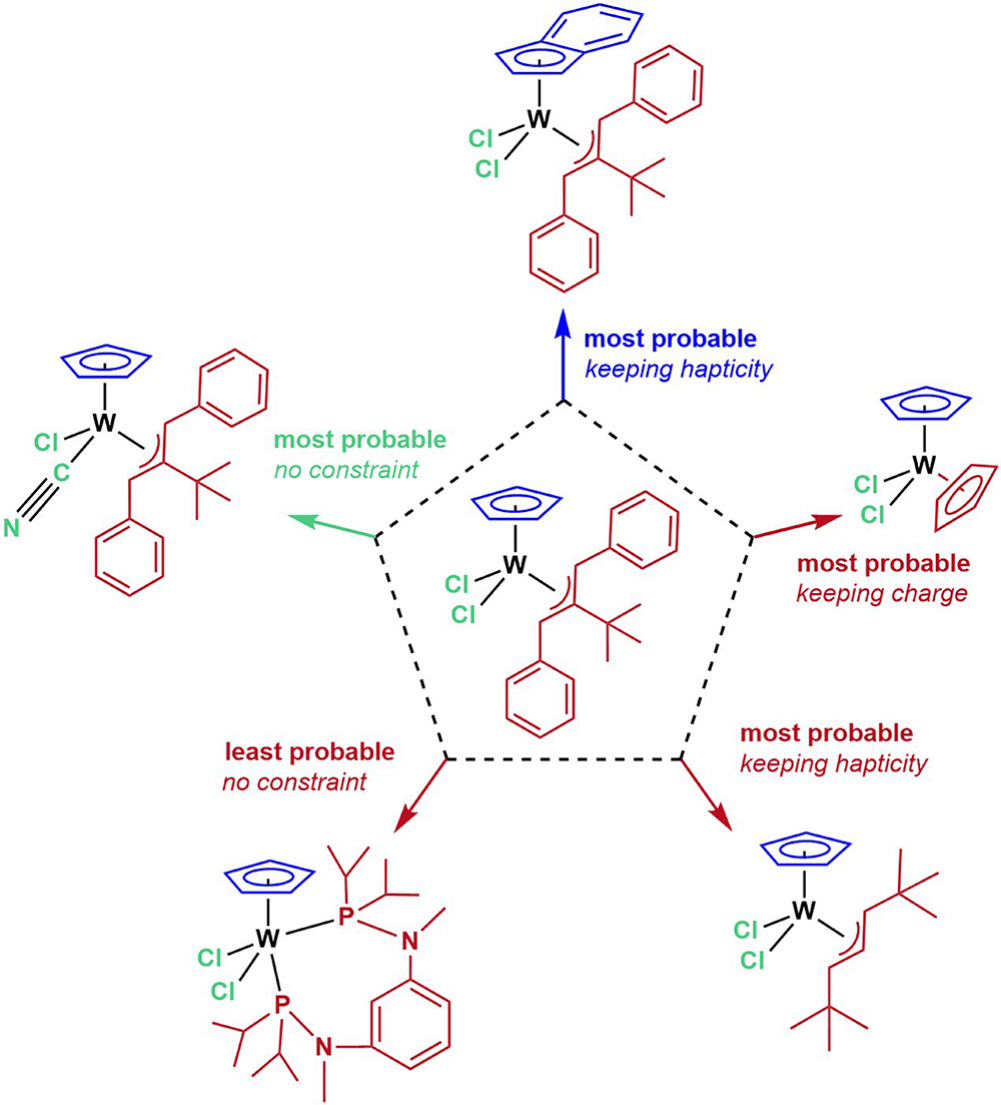
Possible ligand substitutions proposed by our model for the TMC CAKRAH (*earlyTM*).

In the ILUYOD TMC (Figure 10), the most probable replacement of the bidentate phosphine was the popular *para*-methyl substituted bipyridine ligand, when keeping denticity. In contrast, the least probable, unconstrained replacement introduced a rarer tridentate NCP-pincer ligand that changed the coordination number of the metal center. Also, in line with chemical intuition, the most probable replacement of the allyl ligand was either bromide, when keeping the charge, or the common 1,3-*bis*-phenyl substituted allyl ligand, when keeping the hapticity order.

**Figure 10:**
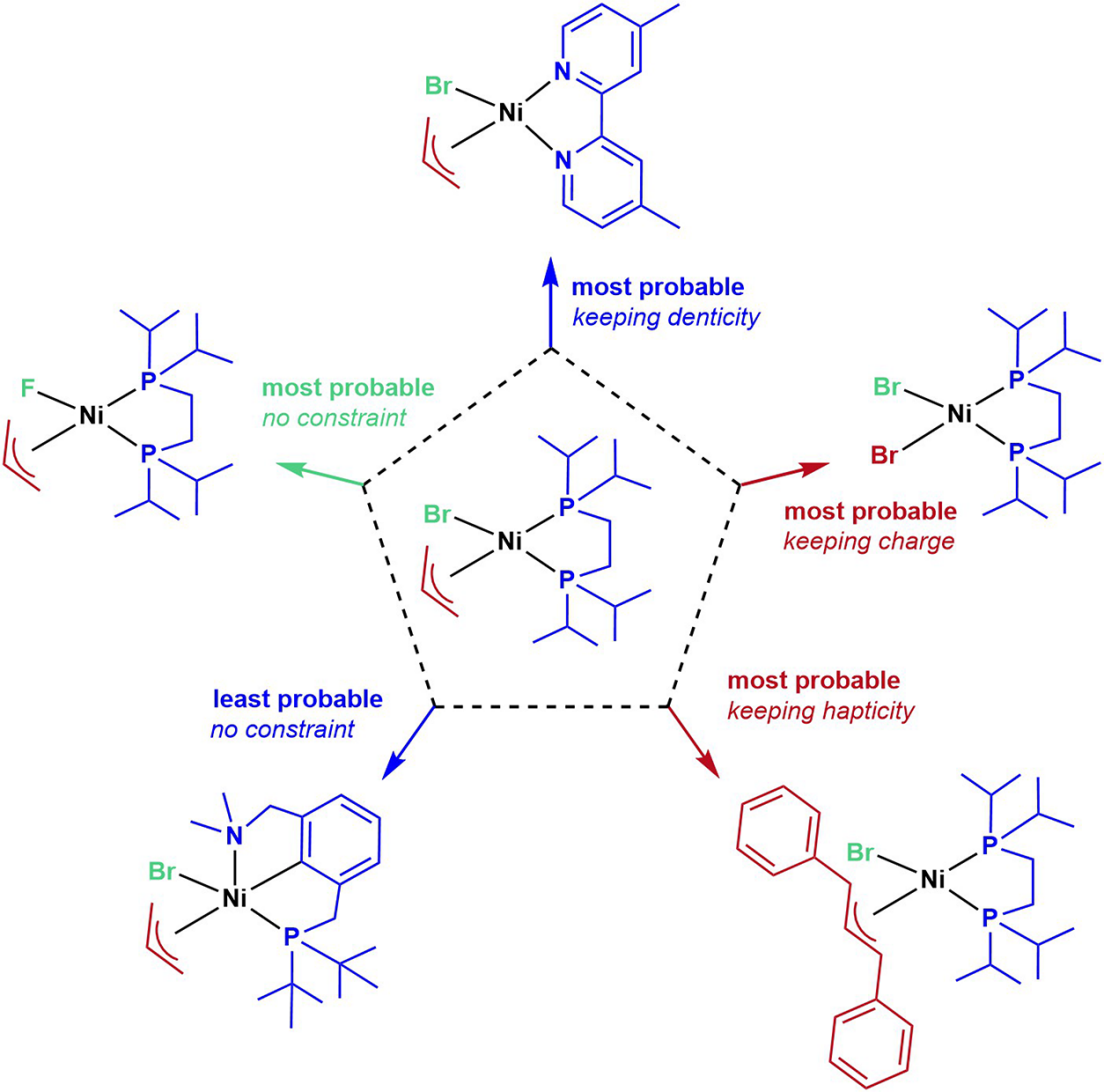
Possible ligand substitutions proposed by our model for the TMC ILUYOD (*lateTM*).

## 6 Conclusions

In this paper we introduce the tmQM-RDF knowledge graph. This resource collects detailed information on a large population of TMCs, by carefully extracting, aggregating, and processing the data that have been collected in the tmQM data set series. At the core of this effort lies a dedicated vocabulary, based on the RDF syntax, specifically designed to make chemical and structural information explicit, interpretable and accessible.

It is indeed in the accessibility of the data that our proposal produces its most valuable contribution, as it allows for the extraction of heterogeneous data from a unified source using the SPARQL query language. Possible queries can be arbitrarily complex, targeting for instance quantitative properties or the structural composition of a TMC, either in therms of atoms or ligands. Even in the case in which it is not possible or convenient to retrieve all the desired information from tmQM-RDF, our knowledge graph still includes all the relevant keys used by the original data sets to index the data, hence more complex investigations can be easily carried out starting from the response provided by tmQM-RDF.

We showed how a simple use case aimed at TMC manipulation can benefit from the formulation used by tmQM-RDF. In particular, we showed that it is possible to extract extensive amounts of relevant structural information that spans both the ligand and the atomic level of a TMC by relying on the SPARQL graph pattern formalism. A relatively simple probabilistic model, such as a Bayesian Network, was capable of harnessing the signal embedded in those patterns, in order to successfully reconstruct a new population of TMCs, generally outperforming simple heuristics-based baselines relying on empiric ligand frequency. This suggests a promising future development in the field of data-driven computational TMC manipulation and analysis.

## Supplementary Material



## Data Availability

The latest release of the tmQM-RDF knowledge graph can be downloaded using the companion package tmqmrdfdata or, alternatively, from https://github.com/luca-cibinel/tmQM-RDF. The latter also contains the development version of tmQM-RDF and the code used in the experiments of this paper. A summary of all the parameters involved in the creation of tmQM-RDF and in the experiments is available in the SI.

## Footnotes

a In other words, they behave as *existentially quanitified variables*.^29,36^

b For brevity, we shall still refer to the group formed by tmQM, tmQMg and tmQMg-L as the *tmQM data set series*. There is no risk of ambiguity, as no other data set is included in this work.

c The abstract ligand-level representation of the metal center is treated as a separate (albeit related) entity from the actual atom, which pertains to the atomic level instead. For this reason, the chemical species considered here is not an actual entry of the periodic table, but rather a virtual chemical species used exclusively at the ligand level. Consider, for instance, lgCr:MetalCentre_Pt versus tmAr:Pt. Both represent the element Pt, but the former is used at the ligand level, whereas the latter is used at the atomic level.

d Hapticity and denticity orders are also explicitly defined as part of the properties of a ligand species, as outlined in the SI.

e Out of the 74,548 TMCs originally extracted from tmQMg, this step removes 15,405 of them (approximately 21*%*), leaving 59,143 viable TMCs.

f This step does not remove any TMC, leading to the final data set size of 59,143 TMCs.

g This fact has been confirmed by the authors of tmQMg-L and can also be verified by inspecting the code in the tmQMg-L GitHub repository.^14^

h “Plausibility”, in this case, should be interpreted as *plausibility given the known and reported TMCs that met the criteria for inclusion in tmQM-RDF*. As tmQM-RDF ultimately derives from the CSD through an extensive series of filters and subsampling procedures, the reference chemical population available for this experiment is limited compared to the entirety of the theoretically admissible chemical compounds.^17^ Another consequence is that any set of features that can be extracted from the training data will only allow to discriminate between structures which are *similar* or *dissimilar* from the (possibly biased) reference population. Differentiation between *valid but unseen* and *invalid* chemical compounds is not possible under the proposed framework.

i If the original ligand possesses the denticity and hapticity orders κ^*n*_0_^, η^*n*_1_^, ···, η^*n*_*S*_^ and the candidate replacement possesses the orders κ^*m*_0_^, η^*m*_1_^, ···, η^*m*_*Q*_^, the filter “*same hapticity and same denticity orders*” is satisfied if and only if *S* = *Q* and *n*_0_ = *m*_0_, *n*_1_ = *m*_1_, ···, *n*_*S*_ = *m*_*S*_.

j To account for potential ties, we define top–*k* accuracy as the average Hit@*k* measure.^49^ In addition, as our method is not designed to appropriately differentiate among ligands which share the same connectivity with the metal center, when sorting ties ligands with the same binding modalities are considered equivalent.

k See Note h.

## Notes

Additional information, including theoretical notions and technical implementation details; introductory explanation of the Resource Description Framework; comprehensive list of RDF prefixes and URIs used in tmQM-RDF; sampling procedures for the two selections mentioned in Section 4.1 (PDF)
