# Supplementary material for: tmQM-RDF Data Set: A Knowledge Graph Representing Transition Metal Complexes
Source: J Chem Inf Model. 2026 Jun 24;66(13):7524–38. doi: 10.1021/acs.jcim.6c01281 (PMC13370852; doi:10.1021/acs.jcim.6c01281)
Supplement: Supplementary file 1 [file ci6c01281_si_001.pdf]

Supporting Information
tmQM-RDF Dataset: a Knowledge Graph Representing Transition Metal Complexes

Luca Cibinel,[*,†,‡] Trond Linjordet,[¶,§] Johan Pensar,[†,‡] David Balcells,[‖] Riccardo De Bin,[†,‡] and Basil Ell[†,⊥,#]

†*Integreat – Norwegian Centre for Knowledge-driven Machine Learning, 0851 Oslo, Norway*

‡*University of Oslo – Department of Mathematics, P.O. Box 1053, Blindern, 0316, Oslo, Norway*

¶*University of Oslo – Department of Chemistry, P.O. Box 1033, Blindern, 0315 Oslo, Norway*

§*Hokkaido University – Institute for Chemical Reaction Design and Discovery (WPI-ICReDD), Sapporo 001-0021, Japan*

‖*University of Oslo – Hylleraas Centre of Excellence for Quantum Molecular Sciences, Department of Chemistry, P.O. Box 1033, Blindern, 0315 Oslo, Norway*

⊥*Bielefeld University – Center for Cognitive Interaction Technology, 33619 Bielefeld, Germany*

#*University of Oslo – Department of Informatics, P.O. Box 1080, Blindern, 0316, Oslo, Norway*

E-mail: lucaci@math.uio.no

Contents

S1 An Introduction to the Resource Description Framework S4

S1.1 Knowledge Graphs and the Resource Description Framework S4

S1.1.1 The Resource Description Framework S5

S1.1.2 The RDF Schema Extension S7

S2 The Documentation of the Terminology Component of tmQM-RDF S10

S2.1 Structural Representation S11

S2.1.1 Whole TMC S11

S2.1.2 Ligands and Metal Centre S13

S2.1.3 Ligand-Metal Centre Bonds S14

S2.1.4 Atoms S15

S2.1.5 Atomic Bonds S16

S2.1.6 Connections Between Levels S16

S2.2 Properties S17

S2.2.1 General Property Description Scheme S17

S2.2.2 Non-Elementary Properties S19

S2.2.3 Abstract Ligand Properties S20

S2.2.4 TMC Metadata S21

S2.2.5 Information for Computational Reproducibility S21

S2.3 Comprehensive tmQM-RDF Namespaces, Classes, and Properties List S22

S3 Thematic Selections for Experimental Purposes S22

S4 Experimental Methods S31

S4.1 Graph Patterns S32

S4.1.1 Frequent Pattern Mining and Data Exploration S33

S4.1.2 Filtering by Relevance S34

S4.2 Elementary Graph Pattern-Based Features S35

S4.3 Clustering Into Families of Substructures S37

S4.4 Aggregated Graph Features S41

S4.5 Probability-Based TMC Scoring Model S42

S4.5.1 Learning the DAG S43

S4.5.2 Parameter Estimation S45

S4.6 The Complete Training Phase S45

S5 Technical Details About Frequent Pattern Mining S46

S5.1 Notation S47

S5.2 Preliminary Definitions S48

S5.3 Frequent Pattern Mining Algorithm S49

S6 Isolating Interesting Patterns S51

S7 Implementation of Pattern Matching S52

S8 On the Domination Relationship Between Graph Patterns S53

S9 Similarity Metrics S56

S9.1 Cosine Similarity S57

S9.1.1 Proxy Feature Vectors S57

S9.1.2 Semantic Feature Vectors S58

S9.2 Similarity Metric for DLGs S60

S9.2.1 Naive Weighting Scheme S62

S9.2.2 Learned Semantic Weights S65

S10 Computational Parameters S67

S11 Extensive Experimental Results S68

S11.1Discussion S68

S1 An Introduction to the Resource Description Framework

This section introduces the concept of knowledge graph and the vocabulary of the Resource Description Framework (RDF) and of its extension RDF Schema (RDFS).

S1.1 Knowledge Graphs and the Resource Description Framework

A *knowledge graph* (KG) can be roughly defined as a "*graph of data intended to accumulate and convey knowledge [...], whose nodes represent entities [...] and whose edges represent [...] relations between these entities.*"[1] In practice, a KG is usually modelled as a *directed labelled graph* (DLG), i.e., a set of labelled nodes (entities) paired with a set of labelled edges (relations). Labelled edges, together with their endpoints, are supposed to encode elementary sentences of the form

$$\text{subject} \xrightarrow{\text{predicate}} \text{object},$$

such as

$$\text{KCEYPT} \xrightarrow{\text{hasMetalCentre}} \text{Pt}.$$

More formally, given a (possibly infinite) set of terms $\mathcal{T}$, a DLG can be defined as a subset $G \subseteq \mathcal{T}^3$, thus as a set of triples $(t_s, t_p, t_o) \in G$.[2] The first and the third element represent, respectively, the *subject* and the *object*, and are to be interpreted as the *nodes* of the graph. The *edges* of G are identified by the triples themselves, with the convention that the edge is directed from t_s (the tail) to t_o (the head), with the label being t_p. This formulation is highly flexible, as it does not prescribe any specific scheme or topological constraint (like tree-shaped graphs). As a consequence, the resulting knowledge base can be easily modified: new pieces of information can be added by simply adding new triples. This flexibil-

ity, together with the availability of a standardised vocabulary (introduced below), makes this framework particularly accessible, both from a development and a deployment perspective.

Before proceeding, it is fundamental to stress the asymmetric treatment of nodes and edges implicitly stated in this definition, in order to avoid confusion. While edges require a label assignment, via a triple structure, nodes are identified with the term (i.e., the label) used to represent them. This automatically implies that distinct nodes cannot be labelled using the same term. Contrarily, it is permitted for two different edges to receive the same label t_p but, on the other hand, we adopt here the restriction that at most one edge can exist between any two nodes. This last constraint is particularly restrictive with respect to the KG literature, where it is typically absent, but, as will become clear below, multiple edges are not necessary for this work and hence are prohibited solely for a matter of notational simplicity. This restriction does not lead to any loss of generality as all the experimental methodologies reported in Section 4, in the main text, and Section S4 can be immediately adapted to the multi-edge case. Moreover, since nodes are implicitly defined via edge specifications, a natural consequence is that there can never be an isolated node within a DLG. Finally, the definition of a graph given here is notably different from the classical one, which usually explicitly prescribe a node set and an edge set. Again, this choice is motivated by a desire for simplicity. Further details and more rigorous definitions are available in Section S9.

S1.1.1 The Resource Description Framework

One specific model for KGs that makes use of the DLG formulation is the *Resource Description Framework (RDF)*,[1] which categorises terms into three different classes: *Uniform Resource Identifiers* (URIs), which uniquely identify entities, *literals*, which are meant to represent quantitative data such as strings or numbers, and *blank nodes*, which are only used to state the existence of entities of interest and, in technical terms, behave as existentially quantified variables.[3,4] At the heart of RDF lies the intention of combining flexibility, simplicity, and

```
:molecule1 :hasAtom _:aaa .
_:aaa :isAtom :H .
:molecule1 :hasAtom _:bbb .
_:bbb :hasAtomicNumber “6” .
```

(a)

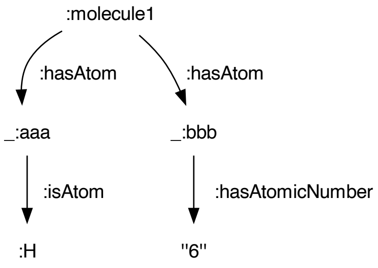

(b)

Figure S1: (a) A simple set of facts, in the form of a set of triples written in Turtle syntax. These facts are stated using URIs (`:*`), blank nodes (`_:*`), and literals (“`*`”). (b) The equivalent directed labelled graph representation of the same set of facts.

power of expressivity and this effort ultimately lead to a solid and standardised framework.[3,5,6]

Using the mathematical language introduced above, if $\mathcal{U}, \mathcal{L}$, and $\mathcal{B}$ are the (pairwise disjoint) sets of URIs, literals, and blank nodes respectively, then $\mathcal{T} = \mathcal{U} \cup \mathcal{L} \cup \mathcal{B}$ and an RDF graph is a DLG $G \subseteq (\mathcal{U} \cup \mathcal{B}) \times \mathcal{U} \times \mathcal{T}$.[2] In other words, literals are only allowed to be placed in object position and predicates may only be represented via URIs.

Example S1.1 describes a simple RDF graph, encoding few elementary facts using the elements just introduced.

Example S1.1. In this example, URIs, blank nodes and literals are all represented as strings. URIs begin with `:`, blank nodes with `_:` and literals are enclosed in quotation marks (“...”).

Here we present a simple knowledge graph that encodes that within a given molecule (`:molecule1`) there exist two atoms (`:hasAtom`), one with chemical label (`:isAtom`) H (`:H`), and the other with atomic number (`:atomicNumber`) 6. Notice that this knowledge base does not explicitly identify the atoms as named entities or resources, but merely states their existence within the molecule, hence the statements will involve two blank nodes (`_:aaa` and `_:bbb`). Figure S1 shows the DLG representation of this set of facts, as well as the corresponding set of triples. △

S1.1.1.1 URI syntax URIs are, as mentioned above, tools meant to uniquely identify a resource. In practice, a URI is a string that satisfies specific syntactical constraints.[5] A more specific class of URI is made up of so called *http URIs*, which identify resources available on the Web and point to the locations of those resources on their respective networks.[7] An example of such URI is

`http://www.w3.org/1999/02/22-rdf-syntax-ns#type`,

which represents a predicate used below (see Section S1.1.2) to express class assignments. This example also shows how URIs can be particularly long and cumbersome to express in plain text, which is why in the RDF literature it is common to find *prefixed names*,[8] i.e. a notation that allows to express URIs by contracting part of the string using uniquely identified *namespaces*. Following the previous example, even though this convention is not restricted to http URIs, we can define the namespace `rdf` as

```
@prefix rdf:  http://www.w3.org/1999/02/22-rdf-syntax-ns#
```

in compliance with the Turtle syntax.[8] In this statement, `@prefix` is an instruction that defines a new namespace, `rdf:` is the chosen namespace ID, and

`http://www.w3.org/1999/02/22-rdf-syntax-ns#`

is the associated URI string. Now, whenever we wish to use the URI above, we can simply use `rdf:type` instead. Prefixes can also be empty strings, as it happened in Example S1.1.[8] We recall that a short summary of the namespaces used in this work is available in Table 1 (in the main document) while the full list can be found in Section S2.3.

S1.1.2 The RDF Schema Extension

RDF alone is a simple assertional language, and it is not capable of specifying descriptions or relations among *types* of objects in a way that also allow for the *entailment* of new

knowledge (for instance, if we know that A is of type X and that X is a subtype of Y, then it is natural to infer that A is also of type Y; this is not natively possible with the sole RDF vocabulary). This functionality is provided by the *RDF Schema* (RDFS) semantic extension, in which proper concepts of *classes* and *subclasses* are introduced, together with a set of entailment rules that define natural inheritance mechanisms.[3,6] Class assignments are expressed via the predicate `rdf:type`, whereas subclass inheritance is expressed via `rdfs:subClassOf`. In a similar way, an appropriate semantic structure regarding properties is introduced. The predicate `rdfs:subPropertyOf` allows to mark two properties as being one a subproperty of the other (and hence the superproperty will apply every time that the subproperty does). The predicates `rdfs:domain` and `rdfs:range`, on the other hand, are used to define the domain and the range of a property (in a similar way to which domain and range are defined for a mathematical function), thus enabling automatic class assignments for the subject and the object of any triple in which the property of interest acts as a predicate.

By leveraging on the RDFS vocabulary, it is possible to separate the content of a knowledge base into two components, namely the terminology box (TBox), a set of assertions about the *concepts* used in the knowledge base, and the assertion box (ABox), a set of assertions on *individual instances and objects*.[9,10] Example S1.2 extends the RDF graph from Example S1.1 by defining an appropriate RDFS terminology.

Example S1.2. Here we extend the RDF graph presented in Example S1.1 by including RDFS terminology (see Figure S2). In particular, we define the classes `:ClassMolecule`, `:ClassAtom`, and `:ClassChemicalElement` that represent molecules, atoms, and chemical labels respectively. Class definition statements are formed by declaring that an object is an instance (`rdf:type`) of `rdfs:Class`. Afterwards, we specify domain and range of the three predicates `:hasAtom`, `:isAtom`, and `:hasAtomicNumber`.
For example, the domain of `:hasAtom` is `:ClassMolecule`, which means that any term found

TBox:

```
:ClassMolecule rdf:type rdfs:Class .
:ClassAtom rdf:type rdfs:Class .
:ClassChemicalElement rdf:type rdfs:Class .
:hasAtom rdfs:domain :ClassMolecule .
:hasAtom rdfs:range :ClassAtom .
:isAtom rdfs:domain :ClassAtom .
:isAtom rdfs:range :ClassChemicalElement .
:hasAtomicNumber rdfs:domain :ClassAtom .
:hasAtomicNumber rdfs:range xmls:integer .
```

ABox:

```
:molecule1 :hasAtom _:aaa .
_:aaa :isAtom :H .
:molecule1 :hasAtom _:bbb .
_:bbb :hasAtomicNumber “6” .
```

Figure S2: The set of facts from Figure S1 is expanded by adding a TBox. The TBox defines several classes that clarify the role and nature of the entities in the ABox, while also specifying appropriate domain and range constraints for the predicates.

in subject position in a triple which has `:hasAtom` as predicate is of class `:ClassMolecule`. Since the triple (`:molecule1`, `:hasAtom`, `_:aaa`) is present in the RDF graph, this means that we can infer the triple (`:molecule1`, `rdf:type`, `:ClassMolecule`), i.e., `:molecule1` is an instance of the class `:ClassMolecule`.

In the case of the predicate `hasAtomicNumber`, the range is defined as `xmls:integer`. This is a class defined within the `xmls` namespace. △

This work makes use of the RDF data model and vocabulary and of the RDFS vocabulary and entailment scheme in order to provide semantic representations of TMCs. There are also other frameworks that can be used to model KGs, such as *property graphs*, which also allow for the annotation of nodes with key-value pairs. While such a framework could seem to be more desirable in a context in which quantitative properties (like node features) are naturally paired with structural descriptors, this formulation is not as standardised as RDF.[1] For simplicity, then, RDF and RDFS are the chosen means of representation.

S2 The Documentation of the Terminology Component of tmQM-RDF

In order to achieve a functional RDF representation that adheres to the principles of the three-level hierarchical representation introduced in the main text, there are several modelling decisions that have to be carefully considered.

With regards to the encoding of the structural component of TMCs, appropriate classes and predicates have to be introduced to represent:

1. the *TMC*, as an entity to which properties that apply to the complex as a whole can be attached;

2. the *ligands* and the *metal centre*, as abstract structural components and specific instances of those abstractions;

3. the *ligand-metal centre bonds*, as entities representing the bonds between structural components and their properties;

4. the *atoms*, both as abstractions and physical instances, similarly to the ligands and the metal atom;

5. the *atomic bonds*, as representations of the bonds between atoms and their properties;

6. the *connections between levels*, intended as containment relationships meant to specify which structural elements belong to which higher level structure (e.g., which atoms are part of a given ligand).

Structural information must then be enriched with quantitative and qualitative features and properties, which poses a new set of challenges:

1. introducing a *general property description scheme*;

2. appropriately describing *non-elementary features* (intended as features which cannot be represented with a single numerical or categorical value);

3. separating *abstract ligand properties* from those of actual instances of chemical entities;

4. appropriately describing *TMC metadata*;

5. appropriately referencing *information for computational reproducibility.*

We will consider each of these points in detail in the rest of the section. A complete summary of the resulting TBox is schematised in Figure S3. We shall also use the example ABox presented in Figure 1, in the main text, as a running example to provide illustrations for the most fundamental concepts. For simplicity, this ABox is reported in Figure S4. Contextually, this example ABox will also demonstrate another feature introduced in tmQM-RDF, namely that the TMCs and all the substructures therein are referenced using the CSD code (six alphabetic characters) that identifies the TMC. For instance, within the subgraph of the tmQM-RDF KG that describes the TMC that in the CSD is listed as KCEYPT, all the URIs that pertain to ligands and atoms of this TMC will include the KCEYPT code.

S2.1 Structural Representation

S2.1.1 Whole TMC

At the complex level, the TMC is the only structural feature, hence a single RDFS class is sufficient for adequate representation. For this purpose, the class `cmT:TransitionMetalComplex` has been defined and one of its instances is used to summarise the complex, interpreted as a single entity. In Figure S4, this instance is represented by the URI `cmT:KCEYPT`. In this case, the class assignment is implicit and performed via the domain specification of the predicates that apply to the node.

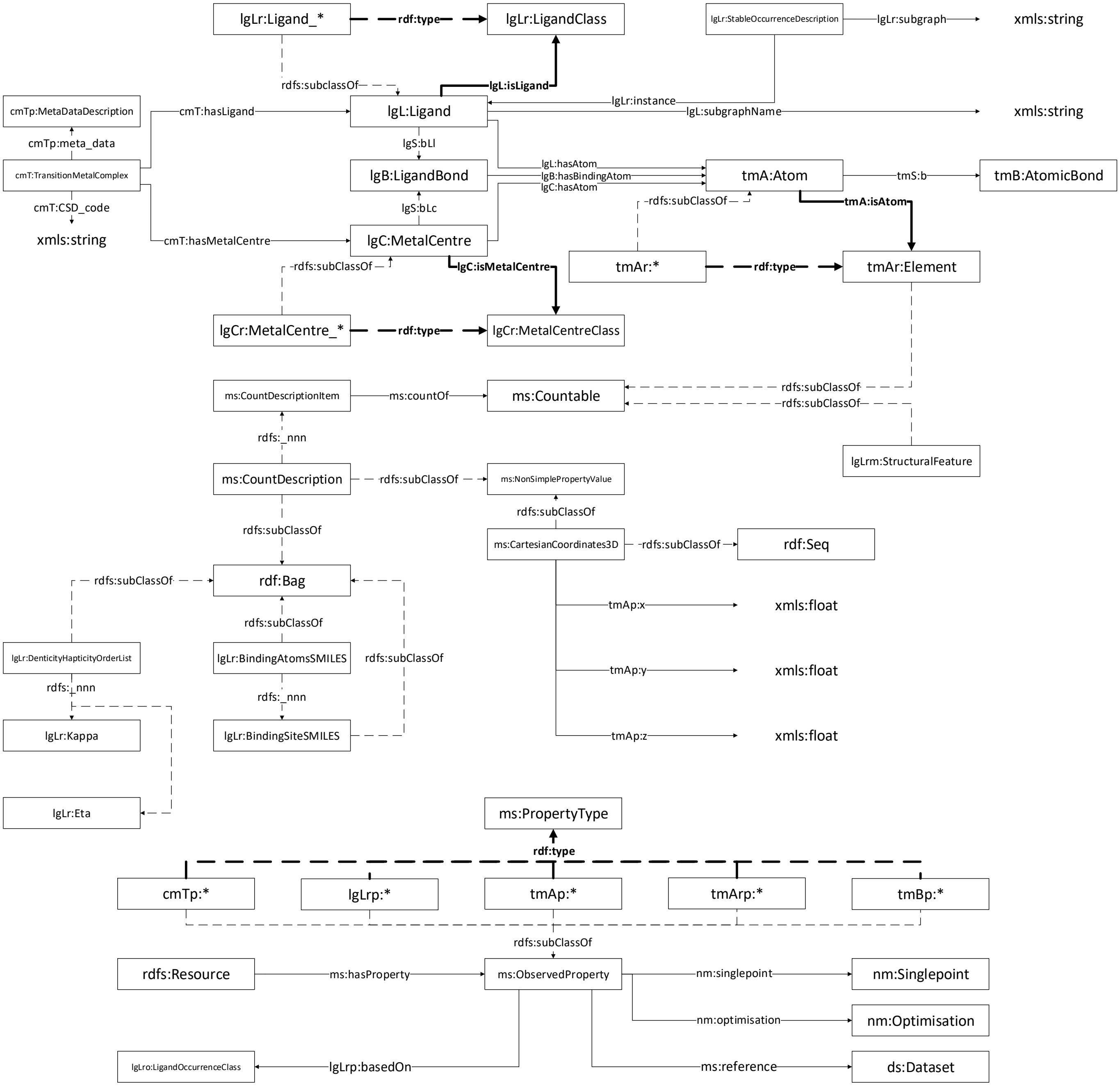

Figure S3: Statements about general concepts are collected in the TBox. Here the TBox of tmQM-RDF is visually represented. Nodes represent the available classes. Solid edges represent the available predicates, where the tail and the head of the edge represent domain and range restrictions for that predicate. Dashed edges, instead, represent class-related assertions, i.e. subclass relationships between classes or class assignments, or other RDFS infrastructural properties and are therefore not indented as domain/range restrictions. Bold edges highlight the predicate `rdf:type` and its subproperties (notice that an edge can be both solid and bold or both dashed and bold). The symbol * is used as a placeholder for a sequence of charachters, representing chemical elements symbols (in `tmAr:*` and `lgCr:MetalCentre_*`), ligand ids (in `lgLr:Ligand_*`), property names (in `cmTp:*`, `lgLrp:*`, `tmAp:*`, `tmArp:*`, and `tmBp:*`), or other miscellaneous items.

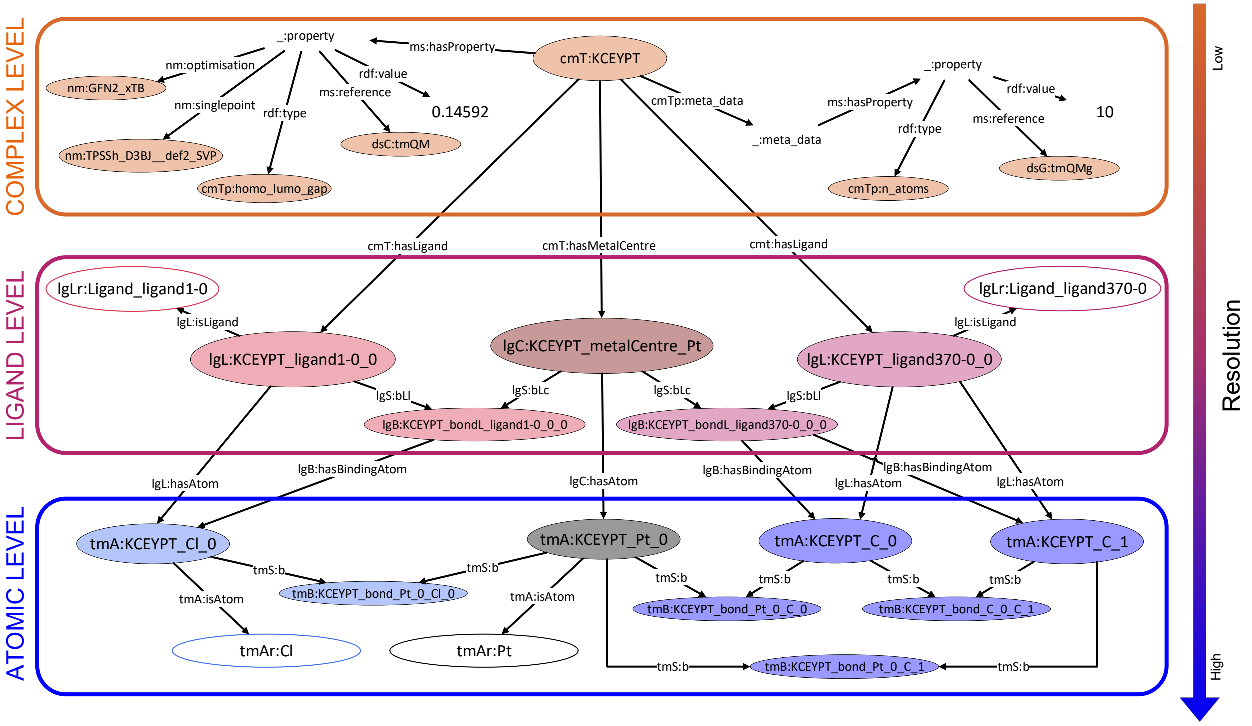

Figure S4: A visual example of an ABox compliant with the tmQM-RDF TBox, showcasing how the data from the tmQM series can be represented. Nodes with neither background nor border represent either blank nodes (`_:*`) or literals (“*”). Nodes with a white background represent RDFS classes. The remaining nodes represent instances of classes. For the sake of readability, not all the features and literal datatypes are represented.

S2.1.2 Ligands and Metal Centre

At the ligand level URIs representing either the metal centre or one of the ligands are assigned a chemical identity (in the tmQMg-L sense).

In the case of ligands, the predicate `lgL:isLigand`, a subproperty of `rdf:type`, assigns a subclass of `lgL:Ligand` to a URI. For instance, `lgLr:Ligand_ligand1-0` represents the ligand that in tmQMg-L is identified as “ligand1-0” (i.e., a single Cl atom). This subclass is itself an instance of `lgLr:LigandClass` and codifies the information on the chemical identity of the ligand.

An example of this scheme, as shown in Figure S4, is the triple

$$\texttt{lgL:KCEYPT\_ligand1-0\_0} \xrightarrow{\texttt{lgL:isLigand}} \texttt{lgLr:Ligand\_ligand1-0}.$$

Here, lgL:KCEYPT_ligand1-0_0 is a URI that represents the first instance of ligand1-0 found within KCEYPT. Moreover, lgLr:Ligand_ligand1-0 is a subclass of lgL:Ligand and an instance of lgLr:LigandClass. This means that the following triples can also be found within the tmQM-RDF knowledge graph:

$$\texttt{lgLr:Ligand\_ligand1-0} \xrightarrow{\texttt{rdfs:subClassOf}} \texttt{lgL:Ligand}$$
$$\texttt{lgLr:Ligand\_ligand1-0} \xrightarrow{\texttt{rdf:type}} \texttt{lgLr:Ligand\_Class}.$$

Moreover, since lgL:isLigand is a subproperty of rdf:type, the RDFS axioms allow us to infer[I] the triple

$$\texttt{lgL:KCEYPT\_ligand1-0\_0} \xrightarrow{\texttt{rdf:type}} \texttt{lgL:Ligand}.$$

The metal centre is handled in an analogous way, using the predicate lgC:isMetalCentre and the classes lgC:MetalCentre and lgCr:MetalCentreClass respectively. An example of a subclass of lgC:MetalCentre (and instance of lgCr:MetalCentreClass) is lgCr:MetalCentre_Pt.

S2.1.3 Ligand-Metal Centre Bonds

A bond between a ligand and the metal centre is represented via one or more instances of the class lgB:LigandBond, to which the ligand and the centre are connected via the predicates lgS:bLl (which connects the ligand) and lgS:bLc (which connects the centre). One new lgB:LigandBond instance (and the corresponding predicates) is introduced for each binding site. In case of hapticity, this is is dealt with through the connection with the atomic level, described below.

In Figure S4, the bond between the metal centre (lgC:KCEYPT_metalCentre_Pt) and the

[I] The entailment follows from the RDFS entailment rules rdfs7 and rdfs9.[3]

ligand `lgL:KCEYPT_ligand1-0_0` introduced above is represented as

$$\texttt{lgL:KCEYPT\_ligand1-0\_0} \xrightarrow{\texttt{lgS:bLl}} \texttt{lgB:KCEYPT\_bondL\_ligand1-0\_0\_0}$$
$$\texttt{lgC:KCEYPT\_metalCentre\_Pt} \xrightarrow{\texttt{lgS:bLc}} \texttt{lgB:KCEYPT\_bondL\_ligand1-0\_0\_0}.$$

S2.1.4 Atoms

At the atomic level, atoms are represented using the same mechanism used for the metal centre and the ligands. The predicate `tmA:isAtom` (again, a subproperty of `rdf:type`) assigns a chemical label (i.e., an element) to a URI. The label is expressed as an instance of the class `tmAr:Element` and a subclass of `tmA:Atom`, and it is meant to represent the chemical element of the atom (e.g., `tmAr:C` for carbonium).

Referencing Figure S4 once more, the Cl atom in `lgL:KCEYPT_ligand1-0_0` is represented via

$$\texttt{tmA:KCEYPT\_Cl\_0} \xrightarrow{\texttt{tmA:isAtom}} \texttt{tmAr:Cl}.$$

The TBox statements about `tmAr:Cl` are

$$\texttt{tmAr:Cl} \xrightarrow{\texttt{rdfs:subClassOf}} \texttt{tmA:Atom}$$
$$\texttt{tmAr:Cl} \xrightarrow{\texttt{rdf:type}} \texttt{tmAr:Element},$$

again with the possibility of inferring[II]

$$\texttt{tmA:KCEYPT\_Cl\_0} \xrightarrow{\texttt{rdf:type}} \texttt{tmA:Atom}.$$

[II] Once again, this relies on the RDFS entailment rules `rdfs7` and `rdfs9`.[3] Recall that `tmA:isAtom` is also a subproperty of `rdf:type`.

S2.1.5 Atomic Bonds

As with the ligand level bonds, chemical bonds between atoms are represented by instances of the class tmB:AtomicBond, to which the involved atoms are connected via the predicate tmS:b.

An example from Figure S4, which is the atomic level-equivalent of the ligand level bond showed above, is

$$\texttt{tmA:KCEYPT\_Cl\_0} \xrightarrow{\texttt{tmS:b}} \texttt{tmB:KCEYPT\_bond\_Pt\_0\_Cl\_0}$$
$$\texttt{tmA:KCEYPT\_Pt\_0} \xrightarrow{\texttt{tmS:b}} \texttt{tmB:KCEYPT\_bond\_Pt\_0\_Cl\_0}.$$

Notice that, in this case, a single predicate is used for all the participants in the bond (both atoms), unlike in the ligand level, in which the two participants have different structural roles (i.e., one is a ligand, the other is the metal centre).

S2.1.6 Connections Between Levels

Each level communicates with the levels below and above through predicates that clarify which components are substructures of which larger structure (e.g., which atom belongs to which ligand).

At the complex level, the instance of the cmT:TransitionMetalComplex class is linked to the metal centre and the ligand objects at the ligand level through the predicates cmT:hasMetalCentre and cmT:hasLigand, respectively. As anticipated, the domain of both these predicates serves as the class declaration of the cmT:TransitionMetalComplex instance.

At the ligand level, each ligand object and the metal centre point to their composing atoms through the predicates lgL:hasAtom (for ligands) and lgC:hasAtom (for the metal centre). At this level, however, there are also objects representing the ligand-metal centre bonds, which also involve atoms. Therefore, it is necessary to introduce similar predicates for

these objects as well. Since the transition metal atom always participates in these bonds, only the atoms that belong to a ligand are highlighted. This is done through the predicates `lgB:hasBindingAtom`, whose domain is the class `lgB:LigandBond`. This predicate is also used to specify the hapticity order of a ligand: if a ligand-metal centre bond involves several adjacent atoms, each of those atoms is the object of a triple whose subject is the same bond object and the predicate is `lgB:hasBindingAtom`.

In Figure S4, the links between the TMC object at the complex level, the ligand examined so far in the previous examples and its single composing atom read as follows:

$$\texttt{cmT:KCEYPT} \xrightarrow{\texttt{cmT:hasLigand}} \texttt{lgL:KCEYPT\_ligand1-0\_0}$$
$$\texttt{lgL:KCEYPT\_ligand1-0\_0} \xrightarrow{\texttt{lgL:hasAtom}} \texttt{tmA:KCEYPT\_Cl\_0}$$
$$\texttt{lgB:KCEYPT\_bondL\_ligand1-0\_0\_0} \xrightarrow{\texttt{lgB:hasBindingAtom}} \texttt{tmA:KCEYPT\_Cl\_0}.$$

For an example of the representation of haptic ligands, we need to look at the other ligand-level ligand object represented in Figure S4, i.e., `lgL:KCEYPT_ligand370-0_0`. This is an haptic ligand as it binds to the metal centre via two adjacent C atoms and, therefore, its bond object, `lgB:KCEYPT_bondL_ligand370-0_0_0`, possesses two binding atoms:

$$\texttt{lgB:KCEYPT\_bondL\_ligand370-0\_0\_0} \xrightarrow{\texttt{lgB:hasBindingAtom}} \texttt{tmA:KCEYPT\_C\_0}.$$
$$\texttt{lgB:KCEYPT\_bondL\_ligand370-0\_0\_0} \xrightarrow{\texttt{lgB:hasBindingAtom}} \texttt{tmA:KCEYPT\_C\_1}.$$

S2.2 Properties

S2.2.1 General Property Description Scheme

Within the scope of this work, any entity (in technical terms, any `rdfs:Resource` instance) can be endowed with one or more properties. In order to express this, the predicate `ms:hasProperty` is used. As shown in Figure S3, the range of `ms:hasProperty` is the class `ms:ObservedProperty`, which in practice is instantiated as a blank node. The reason

behind this choice is that the instances of the class `ms:ObservedProperty` are used merely to summarise the property, but they do not express any information themselves. The actual data about the property is stated by using three additional predicates that act on the blank node: `rdf:type` assigns an instance of `ms:PropertyType` (i.e., a property name, for identifiability), `ms:reference` points to an instance of `ds:Datataset`, representing the original dataset that provided the data and, finally, `rdf:value` states the actual value.

For example, the TMC KCEYPT has a HOMO-LUMO gap (`cmTp:homo_lumo_gap`) that, according to tmQM, amounts to 0.14592 E_h. This is stated in tmQM-RDF as

$$
\begin{aligned}
\texttt{cmT:KCEYPT} &\xrightarrow{\texttt{ms:hasProperty}} \texttt{\_:ppp} \\
\texttt{\_:ppp} &\xrightarrow{\texttt{rdf:type}} \texttt{cmTp:homo\_lumo\_gap} \\
\texttt{\_:ppp} &\xrightarrow{\texttt{ms:reference}} \texttt{dsC:tmQM} \\
\texttt{\_:ppp} &\xrightarrow{\texttt{rdf:value}} \text{“}\texttt{0.14592}\text{”}.
\end{aligned}
$$

A list of all the properties used in this work can be found in Section S2.3.

Each property may require some additional specification or some other consideration, all of which are explained in this section, but all of them employ and extend this baseline structure. The most variable aspect of a property description is its value. Simple properties, such as the HOMO-LUMO gap, can be expressed using a literal. Other properties, like the NBO type of an atomic bond (`tmBp:nbo_type`), use a single URI instead. There are, however, other properties that cannot be described using a single term, and require more complicated structures.

S2.2.2 Non-Elementary Properties

When a single URI or literal does not suffice to specify the value of a property, a blank node, instance of (subclasses of) `ms:NonSimplePropertyValue` is used as the object of `rdf:value`. Several other predicates, applied to the blank node, are then used to fully describe the property value.

Multiple-term values generally follow the scheme of the `rdfs:Container` classes,[6] hence they serve as "collectors" of the information that composes the property value. In tmQM-RDF, such *non-elementary properties* can be tuples of 3D Cartesian coordinates, lists of counts (e.g., element counts or structure counts), lists of binding atoms, lists of hapticity/denticity binding orders, or pointers to the most stable occurrence of a ligand.

In the case of count lists, which we show here as a representative example, the blank node is an instance of `ms:CountDescription`. Via the predicates `rdf:_nnn`, where `nnn` represents a positive integer, the entries of the list are specified. Each entry is itself a blank node and an instance of `ms:CountDescriptionItem`, which specifies the object being counted (an instance of `ms:Countable`), via the `ms:countOf` predicate, and the actual count, via `rdf:value`. A compliant set of triples is:

$$\texttt{cmT:KCEYPT} \xrightarrow{\texttt{cmTp:meta\_data}} \texttt{\_:mmm}$$

$$\texttt{\_:mmm} \xrightarrow{\texttt{ms:hasProperty}} \texttt{\_:ppp}$$

$$\texttt{\_:ppp} \xrightarrow{\texttt{rdf:type}} \texttt{cmTp:element\_counts}$$

$$\texttt{\_:ppp} \xrightarrow{\texttt{ms:reference}} \texttt{dsG:tmQMg}$$

$$\texttt{\_:ppp} \xrightarrow{\texttt{rdf:value}} \texttt{\_:vvv}$$

$$\texttt{\_:vvv} \xrightarrow{\texttt{rdf:type}} \texttt{ms:CountDescription}$$

$$\texttt{\_:vvv} \xrightarrow{\texttt{rdf:\_1}} \texttt{\_:eee}$$

$$\texttt{\_:eee} \xrightarrow{\texttt{rdf:type}} \texttt{ms:CountDescriptionItem}$$

$$\_\text{:eee} \xrightarrow{\texttt{ms:countOf}} \texttt{tmAr:Cl}$$

$$\_\text{:eee} \xrightarrow{\texttt{rdf:value}} \text{“1”}.$$

Lists of binding atoms follow a closely related scheme, with `ms:CountDescription` and `ms:CountDescriptionItem` replaced by `lgLr:BindingAtomsSMILES` and `lgLr:BindingSite-SMILES`. The only difference is that `lgLr:BindingSiteSMILES` is itself a subclass of `rdfs:Container`.

Similarly, lists of binding orders collect all the necessary within an instance of `lgLr:DenticityHapticityOrderList`. Possible items of the list are resources of type `lgLr:Kappa` (for denticity) or `lgLr:Eta` (for hapticity).

For the other available non-elementary properties, i.e., Cartesian coordinates or pointers to the most stable ligand occurrence, the scheme can be simplified. The `rdf:_nnn` predicates are replaced by `tmAp:x`, `tmAp:y` and `tmAp:z` in the case of tuples of Cartesian coordinates (`_:vvv` is of type `ms:CartesianCoordinates3D`), and by `lgLr:subgraph` and `lgLr:instance` for descriptions of stable ligand occurrences (`_:vvv` is of type `lgLr:StableOccurrenceDescription`), and their objects need not to be blank nodes, but can be literals or URIs.

S2.2.3 Abstract Ligand Properties

There are numerous quantitative and qualitative features that pertain to the entities described above. While complex-level and atomic features are attached directly to the entities of interest, contextually to the description of each TMC, properties that refer to entire ligands are reported separately. Since tmQMg-L describes each ligand using general representations that are not specific to any particular instance of the ligands, the properties thereby reported can be thought of as properties of the entire ligand class. For this reason, ligand-level properties are not specified as properties of the physical ligands within TMCs, but rather are listed once, together with the definition of the ligand classes `lgLr:Ligand_*`.

S2.2.4 TMC Metadata

Additional TMC metadata, as defined in tmQMg, is included in each TMC subgraph by introducing a blank node, instance of the class `cmT:MetaDataDescription`. As with the class `ms:ObservedProperty`, this object serves as a summary of the collection of metadata. Actual properties are specified via the usual property description scheme as properties of the metadata blank node (as shown above), which in turn is connected to the TMC node through the `cmTp:meta_data` predicate. Expanding on the aforementioned example, we can find:

$$\texttt{cmT:KCEYPT} \xrightarrow{\texttt{cmTp:meta\_data}} \texttt{\_:mmm}$$
$$\texttt{\_:mmm} \xrightarrow{\texttt{rdf:type}} \texttt{cmTp:MetaDataDescription}.$$

S2.2.5 Information for Computational Reproducibility

For those features for which this information is readily available, the quantum chemistry methods used in the tmQM series are specified together with their `ms:ObservedProperty` objects. The predicates `nm:optimisation` and `nm:singlepoint`, which point to instances of `nm:Optimisation` and `nm:Singlepoint` respectively, indicate the methods used to optimise the molecular geometries and compute their electronic structure properties.

As shown in Figure S4, this information is available for the HOMO-LUMO gap of KCEYPT, hence it is also reported in tmQM-RDF:

$$\texttt{cmT:KCEYPT} \xrightarrow{\texttt{ms:hasProperty}} \texttt{\_:ppp}$$
$$\texttt{\_:ppp} \xrightarrow{\texttt{rdf:type}} \texttt{cmTp:homo\_lumo\_gap}$$
$$\texttt{\_:ppp} \xrightarrow{\texttt{nm:optimisation}} \texttt{nm:GFN2\_xTB}$$
$$\texttt{\_:ppp} \xrightarrow{\texttt{nm:singlepoint}} \texttt{nm:TPSSh\_D3BJ\_\_def2\_SVP}.$$

This means that the HOMO-LUMO gap, for KCEYPT, has been computed using a geometry

optimised at the GFN2-xTB level and then by performing single-point lowest-energy state calculations at the TPSSh-D3BJ/def2-SVP level.

S2.3 Comprehensive tmQM-RDF Namespaces, Classes, and Properties List

This section offers a comprehensive summary of the namespaces, RDFS classes, and properties that can be found in tmQM-RDF. Namespaces are reported in Table S1. The full list of RDFS classes can be found in Table S2. Properties for the complex, ligand, and atomic level, respectively, are listed in Tables S3, S4, and S5.

S3 Thematic Selections for Experimental Purposes

As the tmQM-RDF dataset is extremely rich and diverse, it can be convenient to have access to a smaller subset of TMCs on which to conduct preliminary experiments, such as those presented in Section 4 in the main text. To this end, we here introduce two selections of 1600 TMCs extracted from the population of TMCs in tmQM-RDF, each divided into a training set (1000 TMCs), a validation set (300 TMCs), and a test set (300 TMCs).[III] These two selections have been designed with the intent of replicating the variety found in tmQM-RDF while also restricting the scope of the datasets to a precise chemically relevant subpopulation of complexes. In particular, one selection (the *earlyTM* selection) only contains complexes whose metal centres are either Cr, Mo, W (which are early transition metals) whereas the other (the *lateTM* selection) only admits the metal centres Pd, Ni, Pt (late transition metals; these also happen to be the three most frequent centres that appear in tmQM-RDF, as shown in Figure 3, panel (a), of the main document).

[III]While the experiments proposed in the main text do not require a validation set, more sophisticated frameworks, like conformal prediction,[11] could naturally make use of the validation set, for instance, as a calibration set. For this reason, we include an additional validation partition in the present discussion, to provide a complete and exhaustive documentation.

Table S1: The prefixes used in this paper and the corresponding namespaces. Notice that, for reasons of space, namespaces other than `rdf`, `rdfs`, and `xmls` are specified as relative URIs with respect to the base URI `https://www.integreat.no/research/rdf/tmqm-rdf-dataset/#/`.

Prefix	Namespace	Short description
rdf	http://www.w3.org/1999/02/22-rdf-syntax-ns#	The RDF Concepts Vocabulary (RDF)
rdfs	http://www.w3.org/2000/01/rdf-schema#	The RDF Schema vocabulary (RDFS)
xmls	http://www.w3.org/2001/XMLSchema#	The XML Schema vocabulary
ms	misc/	Non-specific URI path
cm	complex/	Base URI path for complex level terms
cmT	complex/TMC/	General TMC-related facts
cmTp	complex/TMC/property/	TMC properties
lg	ligand/	Base URI path for ligand level terms
lgC	ligand/centre/	General metal centre-related facts
lgCp	ligand/centre/property/	Metal centre properties
lgCr	ligand/centre/reference/	Abstract metal centre representations
lgCrp	ligand/centre/reference/property/	Properties of metal centre representations
lgL	ligand/ligand/	General ligand-related facts
lgLp	ligand/ligand/property/	Ligand properties
lgLr	ligand/ligand/reference/	Abstract ligand representations
lgLrp	ligand/ligand/reference/property/	Properties of abstract ligand representations
lgLro	ligand/ligand/reference/occurrence/	Ligand occurrences used as references
lgLrm	ligand/ligand/reference/motif/	Structural elements of ligand representations
lgB	ligand/bond/	Ligand-centre bond objects
lgBp	ligand/bond/property/	Ligand-centre bond properties
lgBr	ligand/bond/reference/	Abstract ligand-centre bond representations
lgBrp	ligand/bond/reference/property/	Properties of ligand-centre bond representations
lgS	ligand/structure/	Ligand level structural connectivity
tm	atomic/	Base URI path for atomic level terms
tmA	atomic/atom/	General atom-related facts
tmAp	atomic/atom/property/	Atomic properties
tmAr	atomic/atom/reference/	Abstract atom representations
tmArp	atomic/atom/reference/property/	Atomic representation properties
tmB	atomic/bond/	Atomic bond objects
tmBp	atomic/bond/property/	Atomic bond properties
tmBr	atomic/bond/reference/	Abstract atomic bond representations
tmBrp	atomic/bond/reference/property/	Properties of atomic bonds representations
tmS	atomic/structure/	Atomic level structural connectivity
ds	datasets/	Datasets
dsC	datasets/complexes/	tmQM
dsG	datasets/graphs/	tmQMg
dsL	datasets/ligands/	tmQMg-L
nm	numerical/	Numerical methods

Table S2: The RDFS classes employed in tmQM-RDF.

Class	Description
ms:CartesianCoordinates3D	A set of coordinates in the Cartesian coordinate system of the three-dimensional Euclidean space $\mathbb{R}^3$
ms:ComplexPropertyValue	A complex property value that cannot be represented using a single URI/literal
ms:Countable	A countable entity
ms:CountDescription	The description of a count property, intended as a bag of items, each one representing the count of a specific object
ms:CountDescriptionItem	An item in the description of a count property, representing the count of a specific object
ms:ObservedProperty	An observation of a property
ms:PropertyType	A possible property type, intended as one of the many types of properties that are reported in the tmQM series
cmT:TransitionMetalComplex	Transition Metal Complexes
cmTp:MetaDataDescription	An object whose purpose is to collect metadata about a TMC
lgC:MetalCentre	A metal centre
lgCr:MetalCentre_*	A specific metal centre class, subclass of MetalCentre and instance of MetalCentreClass. * must be replaced with the symbol of the desired metal centre
lgCr:MetalCentreClass	A metal centre class, intended as a label that can be assigned to a metal centre
lgL:Ligand	A ligand
lgLr:BindingAtomsSMILES	The description of the binding atoms of a ligand in terms of indices within a SMILES string. It is a collection of BindingSiteSMILES objects
lgLr:BindingSiteSMILES	The description of a group of haptic atoms in a ligand in terms of indices within a SMILES string
lgLr:DenticityHapticityOrderList	The description of the denticity and/or hapticity orders of a ligand
lgLr:Eta	The hapticity order η of a ligand
lgLr:Kappa	The denticity order κ of a ligand
lgLr:Ligand_*	A specific ligand class, subclass of Ligand and instance of LigandClass. * must be replaced with the ID of the desired ligand class
lgLr:LigandClass	A ligand class, intended as a label that can be assigned to a ligand
lgLr:StableOccurrenceDescription	The description of the most stable occurrence of a ligand class, made of a subgraph name and a ligand instance (if available in the dataset)
lgLro:LigandOccurrenceClass	The types of occurrences/representations of a ligand that can be encountered and used for computations (i.e., SMILES string, most stable occurrence, relaxed structure)
lgLrm:StructuralFeature	A structural feature of a ligand that can be counted
lgB:LigandBond	A chemical bond between a metal centre and a binding site within a ligand
tmA:Atom	An atom
tmAr:*	A specific chemical element, subclass of Atom and instance of Element. * must be replaced with the chemical symbol of the desired element
tmAr:Element	A chemical element, intended as a label that can be assigned to an atom
tmB:AtomicBond	A chemical bond between two atoms
tmB:NBOType	A possible NBO type, intended as a label that can be assigned to a chemical bond
ds:Dataset	A dataset
nm:Optimisation	A method for the optimisation of molecular geometries via relaxation of energy gradients
nm:Singlepoint	A method for the computation of the energy and other electronic structure properties of optimized geometries

Table S3: The URIs representing to the complex level properties.

Property	Description
cmTp:CSD_years	Year of the CSD update to which the data refers to
cmTp:charge	Overal charge of the metal complex
cmTp:dipole_moment	Dipole moment in Debyes
cmTp:dipole_moment_delta	Change of dipole moment between different levels of theory
cmTp:dispersion_energy	Dispersion energy
cmTp:dispersion_energy_delta	Dispersion energy difference between the different levels of theory
cmTp:electronic_energy	Electronic energy
cmTp:electronic_energy_delta	Electronic energy difference between the different levels of theory
cmTp:element_counts	Element counts
cmTp:enthalpy_energy	Enthalpy energy
cmTp:enthalpy_energy_correction	Enthalpy energy correction
cmTp:entropy	Entropy energy
cmTp:gibbs_energy	Gibbs energy
cmTp:gibbs_energy_correction	Gibbs free energy correction
cmTp:heat_capacity	Heat capacity
cmTp:highest_vibrational_frequency	Vibrational frequency with the largest value, in cm-1
cmTp:homo_energy	HOMO energy
cmTp:homo_lumo_gap	HOMO-LUMO gap
cmTp:homo_lumo_gap_delta	Change of HOMO-LUMO between different levels of theory
cmTp:lowest_vibrational_frequency	Lowest vibrational frequency in cm-1
cmTp:lumo_energy	LUMO energy
cmTp:metal_center_element	Metal element
cmTp:metal_center_group	Periodic table group of the metal
cmTp:metal_center_period	Periodic table period of the metal
cmTp:metal_node_degree	Metal node degree
cmTp:metal_node_natural_charge	Natural atomic charge of the metal node, in e units
cmTp:molecular_mass	Molecular mass
cmTp:n_atoms	Total number of atoms
cmTp:n_electrons	Total number of electrons in the metal complex
cmTp:polarisability	Overal polarizability of the metal complex
cmTp:spin	Overal spin multiplicity of the metal complex
cmTp:stoichiometry	Stoichiometry of the metal complex, in Hill format
cmTp:zpe_correction	Zero-point energy

Table S4: The URIs representing to the ligand level properties.

Property	Description
lgLrp:G_parameter	Ligand G parameter
lgLrp:I1_over_I3	Moments of inertia I1 and I3 ratio
lgLrp:I2_over_I3	Moments of inertia I2 and I3 ratio
lgLrp:buried_volume	Ligand buried volume
lgLrp:charge	Ligand charge
lgLrp:dentic_element_counts	Number of dentic-metal-bound atoms
lgLrp:denticity_hapticity_orders	The list of denticity and/or hapticity orders of the ligand
lgLrp:dipole_moment	Ligand dipole moment
lgLrp:element_counts	Element-wise count of ligand atoms
lgLrp:exact_cone_angle	Ligand exact cone angle
lgLrp:haptic_element_counts	Element-wise counting of haptic-metal-bound atoms
lgLrp:homo_lumo_gap	Ligand HOMO-LUMO gap
lgLrp:is_alternative_charge	Whether the charge reported in tmQMg-L disagrees with the charge reported in the OctLig dataset for the same ligand
lgLrp:largest_frequency	Ligand largest frequency
lgLrp:logp	Ligand logP
lgLrp:metal_bound_homo_d	d orbital character of metal-bound HOMO
lgLrp:metal_bound_homo_energy	HOMO energy involving metal bound atom
lgLrp:metal_bound_homo_f	f orbital character of metal-bound HOMO
lgLrp:metal_bound_homo_p	p orbital character of metal-bound HOMO
lgLrp:metal_bound_homo_s	s orbital character of metal-bound HOMO
lgLrp:metal_bound_lumo_d	d orbital character of metal-bound LUMO
lgLrp:metal_bound_lumo_energy	Metal-bound LUMO energy
lgLrp:metal_bound_lumo_f	f orbital character of metal-bound LUMO
lgLrp:metal_bound_lumo_p	p orbital character of metal-bound LUMO
lgLrp:metal_bound_lumo_s	s orbital character of metal-bound LUMO
lgLrp:molar_volume	Ligand molar volume
lgLrp:n_atoms	Ligand total number of atoms
lgLrp:n_dentic_bound	Number of dentic-metal-bound atoms
lgLrp:n_metal_bound	Number of metal-bound atoms
lgLrp:polarisability	Ligand polarizability
lgLrp:sasa_area_free	Ligand SASA area (free)
lgLrp:sasa_area_stable	Ligand SASA area (stable)
lgLrp:sasa_volume_free	Ligand SASA volume (free)
lgLrp:sasa_volume_stable	Ligand SASA volume (stable)
lgLrp:smiles_metal_bond_node_idx_groups	Indicies of metal-bound atoms in the SMILES
lgLrp:solid_angle	Ligand coordination solid angle
lgLrp:solid_cone_angle	Ligand solid cone angle
lgLrp:stable_occurrence	The most stable occurrence of a ligand
lgLrp:structure_counts	Counts of selected structures within ligands
lgLrs:SMILES	Ligand SMILES
lgLrp:n_haptic_bound	Number of haptic bonds

Table S5: The URIs representing to the atomic level properties.

Property	Description
tmAp:hydrogen_count	Number of hydrogen atoms bound to atomic node
tmAp:lone_pair_energy_min_max_difference	Energy difference between lowest- and highest-energy lone pair orbitals
tmAp:lone_pair_max_d_occupation	Maximum d occupation of lone pair orbitals
tmAp:lone_pair_max_energy	Maximum energy of lone pair orbitals
tmAp:lone_pair_max_occupation	Maximum electron occupation of the NBO lone pairs
tmAp:lone_pair_max_p_occupation	Maximum p occupation of lone pair orbitals
tmAp:lone_pair_max_s_occupation	Maximum s occupation of lone pair orbitals
tmAp:lone_vacancy_energy_min_max_difference	Energy difference between lowest- and highest-energy lone vacancy orbitals
tmAp:lone_vacancy_min_d_occupation	Minimum d occupation of lone vacancy orbitals
tmAp:lone_vacancy_min_energy	Minimum energy of lone vacancies
tmAp:lone_vacancy_min_occupation	Minimum occupation of lone vacancy orbitals
tmAp:lone_vacancy_min_p_occupation	Minimum p occupation of lone vacancy orbitals
tmAp:lone_vacancy_min_s_occupation	Minimum s occupation of lone vacancy orbitals
tmAp:n_lone_pairs	Number of lone pair orbitals
tmAp:n_lone_vacancies	Number of lone vacancy orbitals
tmAp:natural_atomic_charge	Natural atomic charge, in e units
tmAp:natural_electron_configuration_d_occupation	Electron occupation of the d orbitals
tmAp:natural_electron_configuration_p_occupation	Electron occupation of the p orbitals
tmAp:natural_electron_configuration_s_occupation	Electron occupation of the s orbitals
tmAp:natural_electron_population_valence	Electron occupation of valence orbitals
tmAp:node_id	A unique internal identifier used to distinguish the nodes of the atomic graph
tmAp:node_position	Atom index in associated xyz geometry
tmAp:valency_index	Valency index from NBO analysis
tmArp:atomic_number	Atomic number
tmBp:antibond_energy_min_max_difference	Energy difference between lowest- and highest-energy antibonding orbitals
tmBp:antibond_min_d_occupation	Minimum d occupation of antibonding orbitals
tmBp:antibond_min_energy	Minimum energy of antibonding orbitals
tmBp:antibond_min_occupation	Minimum overall occupation of antibonding orbitals
tmBp:antibond_min_p_occupation	Minimum p occupation of antibonding orbitals
tmBp:antibond_min_s_occupation	Minimum s occupation of antibonding orbitals
tmBp:bond_distance	Bond distance, in Å
tmBp:bond_energy_min_max_difference	Energy difference between lowest- and highest-energy bonding orbitals
tmBp:bond_max_d_occupation	Maximum d occupation of bonding orbitals
tmBp:bond_max_energy	Highest energy of bonding orbitals
tmBp:bond_max_occupation	Maximum overall occupation of bonding orbitals
tmBp:bond_max_p_occupation	Maximum p occupation of bonding orbitals
tmBp:bond_max_s_occupation	Maximum s occupation of bonding orbitals
tmBp:n_bn	Number of bonding orbitals for bond
tmBp:n_nbn	Number of antibonding orbitals for bond
tmBp:nbo_type	NBO type defining the bond
tmBp:wiberg_bond_order	Wiberg bond order

The two selections have been sampled from the full dataset following the procedure below:

1. a “seed” of the N_{seed} most frequent ligands found within the subset of tmQM-RDF identified by the desired metal centres has been computed.

2. a candidate set made of all the complexes whose ligands are all included in the “seed” has been computed;

3. from the candidate set, 1600 TMCs have been sampled with probability inversely proportional to the number of atoms[IV];

4. the train/validation/test split has been performed.

A quick assessment of the representativeness of each selection can be performed by comparing the sets of the most frequent ligands within the selection to the same set extracted from tmQM-RDF (by only considering, however, complexes which have the same metal centres as the selection). Figures S5 and S6 portray the results of this kind of analysis by showing the counts of the occurrences of the most frequent ligands in the original dataset and in the three partitions of each selection. Overall it can be seen that, while the sets of most frequent ligands do not coincide perfectly, for both selections at least half of the ligands retain their status of most frequent across all the datasets (specifically, for the *earlyTM* selection those ligands are *ligand0-0*, *ligand55-0*, *ligand11-0*, *ligand42-0*, *ligand22-0*, *ligand6-0*, and *ligand764-0*, whereas for the *lateTM* selection the ligands are *ligand0-0*, *ligand12-0*, *ligand20-0*, *ligand36-0*, *ligand91-0*, *ligand29-0*, and *ligand28-0*). Moreover, by looking at the expressiveness of each ligand-metal centre pair, it is possible to notice that, especially in the *earlyTM* selection, there are only minor qualitative differences (in the *lateTM* selection, on the other hand, it is more frequent to observe ligands for which one centre becomes overrepresented in the selection with respect to tmQM-RDF).

[IV] In this phase, the relative proportions of TMCs having a given metal centre are constrained to match those found in tmQM-RDF

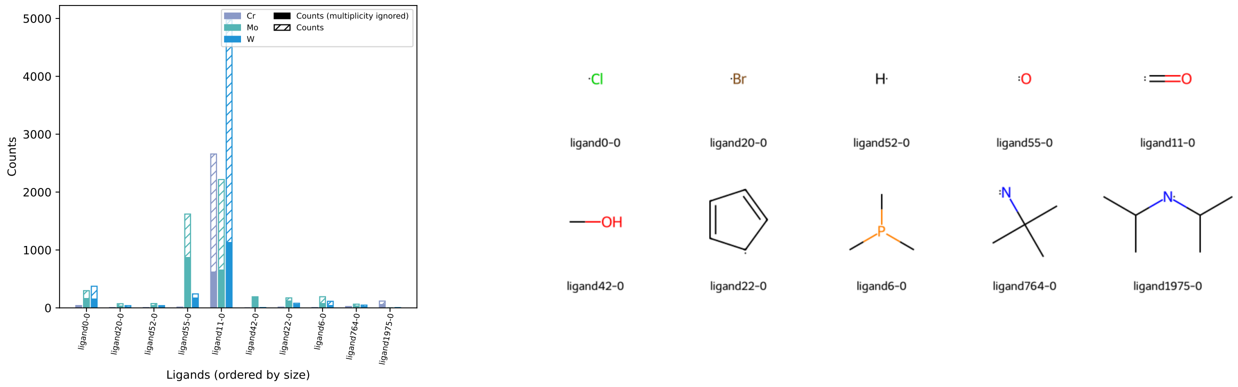

(a) tmQM-RDF (Cr - Mo - W)

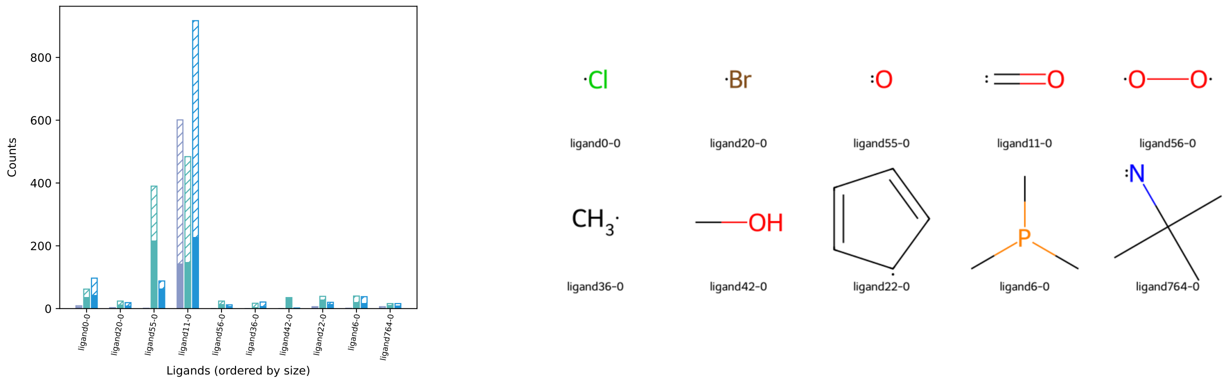

(b) Train

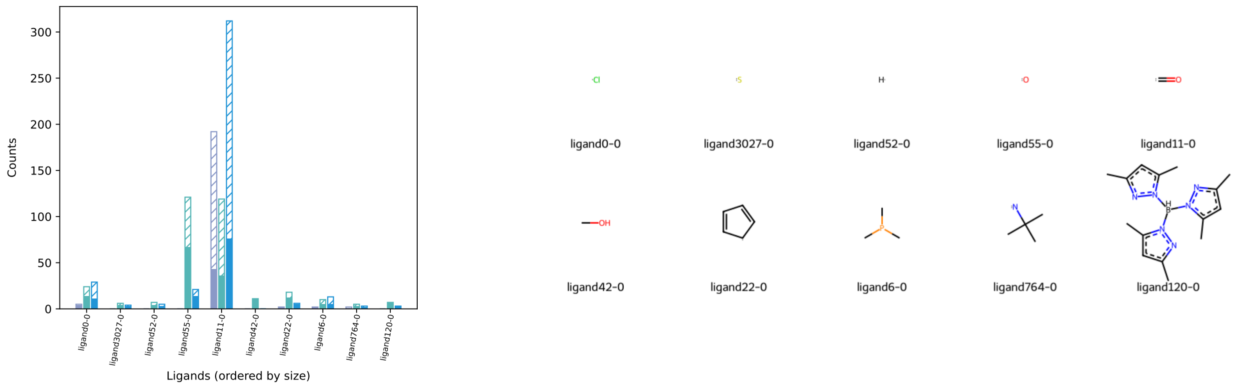

(c) Validation

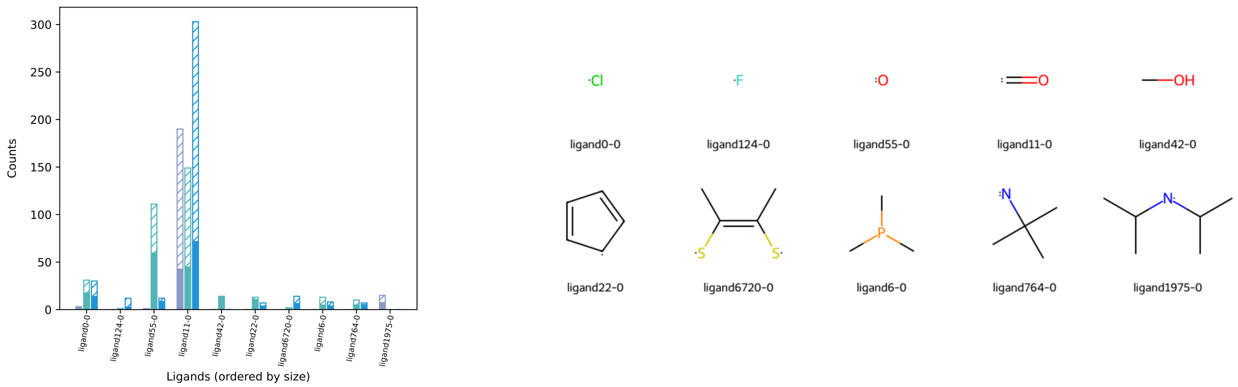

(d) Test

Figure S5: The bar plots of the counts of the appearances of the 10 most frequent ligands, divided by metal centre, (left) and a visual representation of the same ligands (right) in: (a) tmQM-RDF, accounting only for Cr, Mo and W centres, (b), the training set, (c) the validation set and (d) the test set of the *earlyTM* selection. In the barplots, two different counting methods are being represented: in one scenario, every single occurrence of a ligand is considered (dashed bars), in the other, multiple occurrences of a ligand within a single complex are not considered (dashed bars).

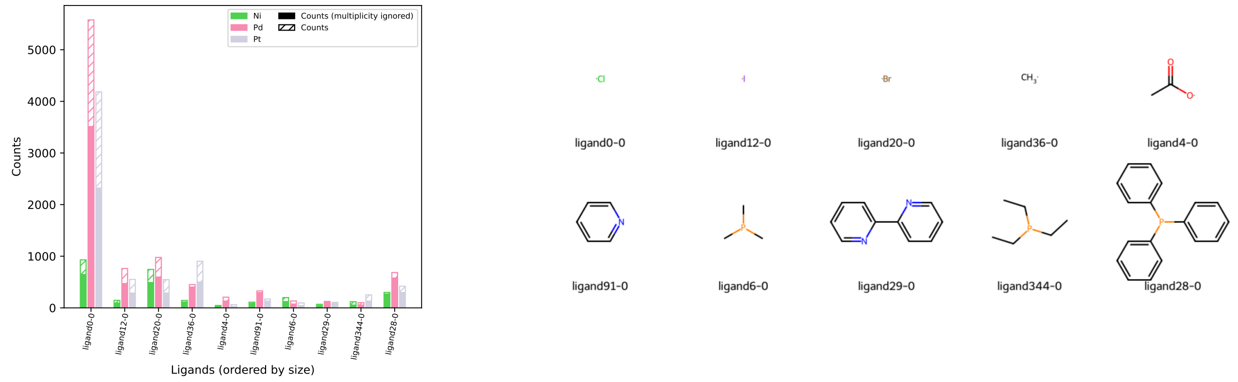

(a) tmQM-RDF (Ni - Pd - Pt)

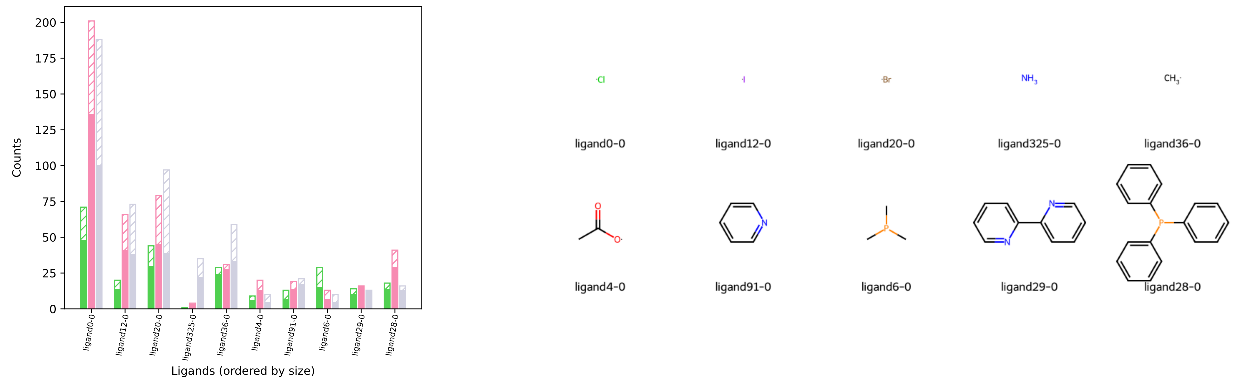

(b) Train

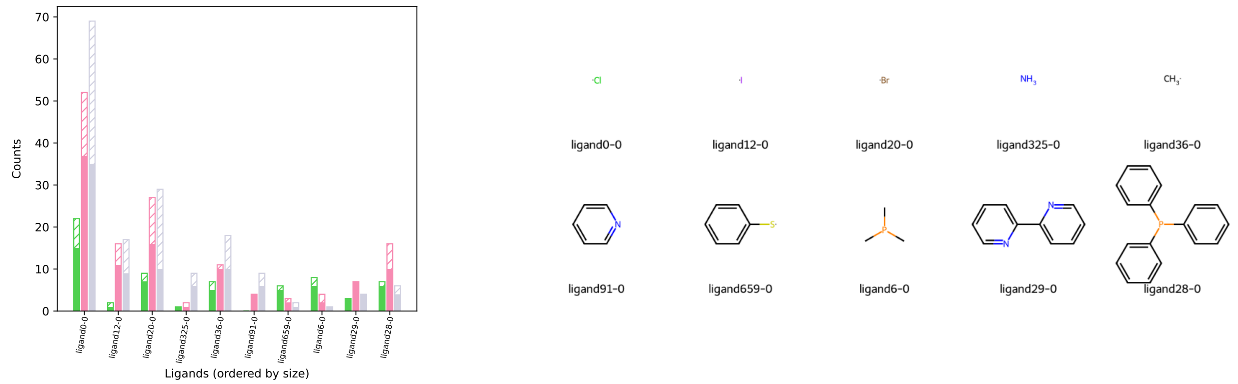

(c) Validation

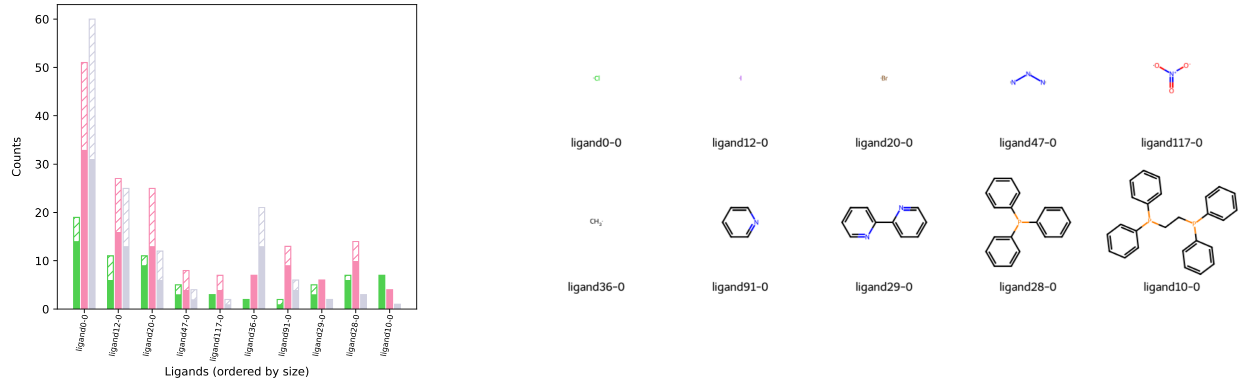

(d) Test

Figure S6: The bar plots of the counts of the appearances of the 10 most frequent ligands, divided by metal centre, (left) and a visual representation of the same ligands (right) in: (a) tmQM-RDF, accounting only for Ni, Pd and Pt centres, (b), the training set, (c) the validation set and (d) the test set of the *lateTM* selection. In the bar plots, two different counting methods are being represented: in one scenario, every single occurrence of a ligand is considered (dashed bars), in the other, multiple occurrences of a ligand within a single complex are not considered (dashed bars).

S4 Experimental Methods

In this section we provide an initial account of the technical details behind the experimental methods employed in Section 4 of the main document.

We recall that the purpose of the experiment is to extract relevant structural features from tmQM-RDF in the form of graph patterns. The patterns are clustered into families of typical substructures, which are then converted into binary graph-level features that indicate whether that family is expressed or not in a given graph. The joint distribution of the features is estimated and used to assign a score to a TMC. New TMCs are obtained by substituting one of the ligands in an existing TMC and then evaluated via the score function.

All the material needed to perform this learning task is formally introduced here. In the following, let $\mathcal{G}$ be the graph dataset representing the population of TMCs which is the object of the study. We partition $\mathcal{G}$ in a training set $\mathcal{G}_{\text{train}}$, used to estimate the score function, and a test set $\mathcal{G}_{\text{test}}$, which will be manipulated on the basis of the estimated score.

In the main text, $\mathcal{G}$ has been simply presented as a subpopulation of the family of TMCs that are described in tmQM-RDF. Here, $\mathcal{G}$ is explicitly defined as a set of RDF graphs, as defined in Section S1.1.1, which correspond each to the subgraph of tmQM-RDF that encodes the information about the respective TMC of interest.

The first problem that has to be addressed is that of appropriately defining what a family of substructures is and how it can lead to the definition of a structural feature, in the context of this work and the tmQM-RDF dataset. Both of these concepts strongly rely on the notion of graph pattern, in the SPARQL sense,[2,12] which is here regarded as the primary data exploration tool used to acquire structural information about the TMCs under examination.

We will give a formal introduction to graph patterns and explain how patterns naturally lead to an elementary definition of a graph feature vector. Afterwards, we will move to the problem of clustering patterns into families of substructures using an agglomerative clustering algorithm[13] based on the notion of graph similarity. Finally, we will define the actual feature

vector we will employ for our score assessment, by accordingly aggregating the elementary features.

These newly-defined features need to be studied from a statistical point of view, with the objective of defining a score function that can assess a TMC on the basis of its structural components. Here, "score" is intended as the log-probability of the resulting feature vector, as measured by an estimate of the joint distribution of the features themselves. The challenges that arise when trying to estimate such a high-dimensional distribution are examined and dealt with by means of the Bayesian Network (BN) formulation.[14,15]

S4.1 Graph Patterns

Informally, a graph pattern is a knowledge graph that employs the same language of the RDF dataset of interest (here, tmQM-RDF), with the addition of variables.
Using the notation introduced in Section S1.1, if $\mathcal{T} = \mathcal{U} \cup \mathcal{L} \cup \mathcal{B}$ is the usual set of terms and $\mathcal{V}$ is a set of *variables*, an *RDF graph pattern* is a graph $p \subseteq (\mathcal{U} \cup \mathcal{B} \cup \mathcal{V}) \times (\mathcal{U} \cup \mathcal{V}) \times (\mathcal{T} \cup \mathcal{V})$.[2,12] The sets of terms and variables that actually appear in p are denoted with $\mathcal{T}_p$ and $\mathcal{V}_p$ respectively.

The presence of variables allows us to employ a graph pattern p as a query against an RDF graph $G \in \mathcal{G}$, by asking if G is expressing (in some sense) the structure represented by p. This leads to the definition of a *match* of p in G, which is intended to be a mapping $\mu : \mathcal{T}_p \cup \mathcal{V}_p \to \mathcal{T}_G$ such that $\mu(t) = t$ for every term $t \in \mathcal{T}_p$ and that $\mu(p) \subseteq G$, where $\mu(p)$ is a shorthand notation for the graph obtained by replacing every node and edge label in p with its image under μ. In fewer words, a match is a replacement (or grounding) of the variables in p such that the resulting graph is a subgraph of G.[2,12]

This work imposes an additional restriction to the definition of μ, known as the *no-repeated-anything graph pattern evaluation semantics*.[2] This means that no variable in p can

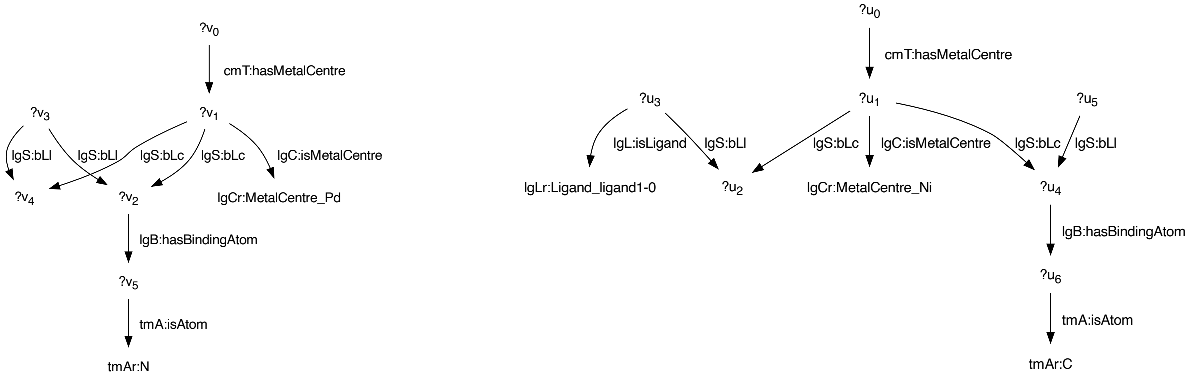

(a) (b)

Figure S7: Two examples of graph patterns. Nodes starting with "?" represent variables. (a) A graph pattern that will match any graph representing a TMC with a bidentate ligand, with N as one of its binding atoms, and Pd as its metal centre. (b) A graph pattern that will match any graph representing a TMC that contains one copy of ligand1-0, another ligand, which has C as one of its binding atoms, and Ni as the metal centre. Notice that, by the no-repeated-anything graph pattern evaluation semantics, these two ligands have to be distinct.

be mapped to a term already present in $\mathcal{T}_p$ nor can two different variables be mapped to the same term (i.e., μ is required to be injective). The set of the matches of p against G under this evaluation semantics is denoted with $\Omega_{G,p}$.

Details on how μ and $\Omega_{G,p}$ are computed in this work, using the SPARQL query language,[12] can be found in Section S7. Figure S7 shows two example graph patterns, while also demonstrating how this concept can be used to capture structural information.

S4.1.1 Frequent Pattern Mining and Data Exploration

Tasks like that of identifying frequent substructures in a dataset of graphs that can be represented using the RDF syntax, as is the case in the proposed experiment, can be naturally addressed by identifying frequently occurring patterns (via pattern mining).

Here, the term *pattern mining* refers to the computation of a set $\widetilde{\mathcal{P}}$ of *frequent patterns*

starting from a training set $\mathcal{G}_{\text{train}}$ of RDF graphs. A pattern p is considered frequent when

$$|\{G \in \mathcal{G}_{train} : \Omega_{G,p} \neq \emptyset\}| \geq \alpha, \tag{S1}$$

for a given user-defined threshold $\alpha \in \mathbb{N}$.

The pattern mining procedure that we employ in order to compute $\widetilde{\mathcal{P}}$ is hierarchical in nature. Given an initial *seed pattern* p_0, i.e., a pattern made of a single triple, the algorithm produces candidate patterns by extending p_0 by one triple at a time and then by evaluating whether these candidates are frequent or not. Subsequent iterations proceed in the same way, by extending each of the patterns found at the previous step using only one triple and by looking for frequent patterns in the resulting set of candidates. The algorithm stops when a maximum number of triples per pattern is reached or no pattern can be extended into a frequent pattern.

The procedure is described at a technical level in Section S5.

Notice that pattern mining is combinatoric in nature, hence it should be expected that the cardinality of $\widetilde{\mathcal{P}}$ will be high.

S4.1.2 Filtering by Relevance

In order to reduce the risk of employing patterns which are scarcely relevant with respect to the task being considered, we compute a subset $\mathcal{P} \subseteq \widetilde{\mathcal{P}}$ of patterns which are both frequent and encode information that we deem to be potentially useful.

In particular, since the experiment focuses on ligands and their binding atoms, we ignore patterns in which no binding atoms are specified. This means that a pattern $p \in \widetilde{\mathcal{P}}$ is considered to be interesting if the existence of at least one binding atom is asserted and the identity of that atom is specified. To avoid redundancy, we also impose that the chemical

identity of the ligand the atom belongs to must not be specified in the same pattern.

This condition alone, however, may be too restrictive, as more unusual TMCs may present configurations which are too rare to be encoded by a pattern which is both frequent and adhering to the rule above. For this reason, we also include in $\mathcal{P}$ patterns which are, in some sense, "precursors" of interesting patterns.

The most intuitive way of defining a precursor of a pattern $p \in \widetilde{\mathcal{P}}$, especially in view of the pattern mining algorithm in Section S4.1.1, is to consider a pattern $q \in \widetilde{\mathcal{P}}$ which is a *subpattern* of p, i.e., $q \subseteq p$. In this simple case, it immediately holds that any match μ_p of p against any graph G can be restricted to a match μ_q of q against that same graph. In particular, if p matches a graph, then q *must* match that graph as well. Equivalently, if q does not match a graph, p *cannot* match that graph.

As it turns out, this last property is more general than the strict subpattern relation. We say that q *dominates* p if, for any RDF graph G, $|\Omega_{G,q}| = 0$ implies $|\Omega_{G,p}| = 0$. This relation may appear significantly more complicated than the subpattern relation, but, as shown in Section S8, it is sufficient to be able to compute pattern matches against an RDF graph to be able to check for pattern domination.

Somewhat informally, we could then define the set $\mathcal{P}$ as the set of frequent patterns which are either interesting, as per the rule given above, or dominate an interesting pattern (see Figure S8). Precise details about the computation of $\mathcal{P}$ are given in Section S6.

S4.2 Elementary Graph Pattern-Based Features

Given a set $\mathcal{P}$ of frequent patterns, it is possible to compute a feature matrix $\widetilde{X} \in \mathbb{N}^{|\mathcal{G}|\times|\mathcal{P}|}$ such that

$$\widetilde{X}_{G,p} = |\Omega_{G,p}|, \tag{S2}$$

where the notation $\widetilde{X}_{G,p}$ implicitly assumes that the entries of $\widetilde{X}$ are indexed by the elements of $\mathcal{G}$ and $\mathcal{P}$.

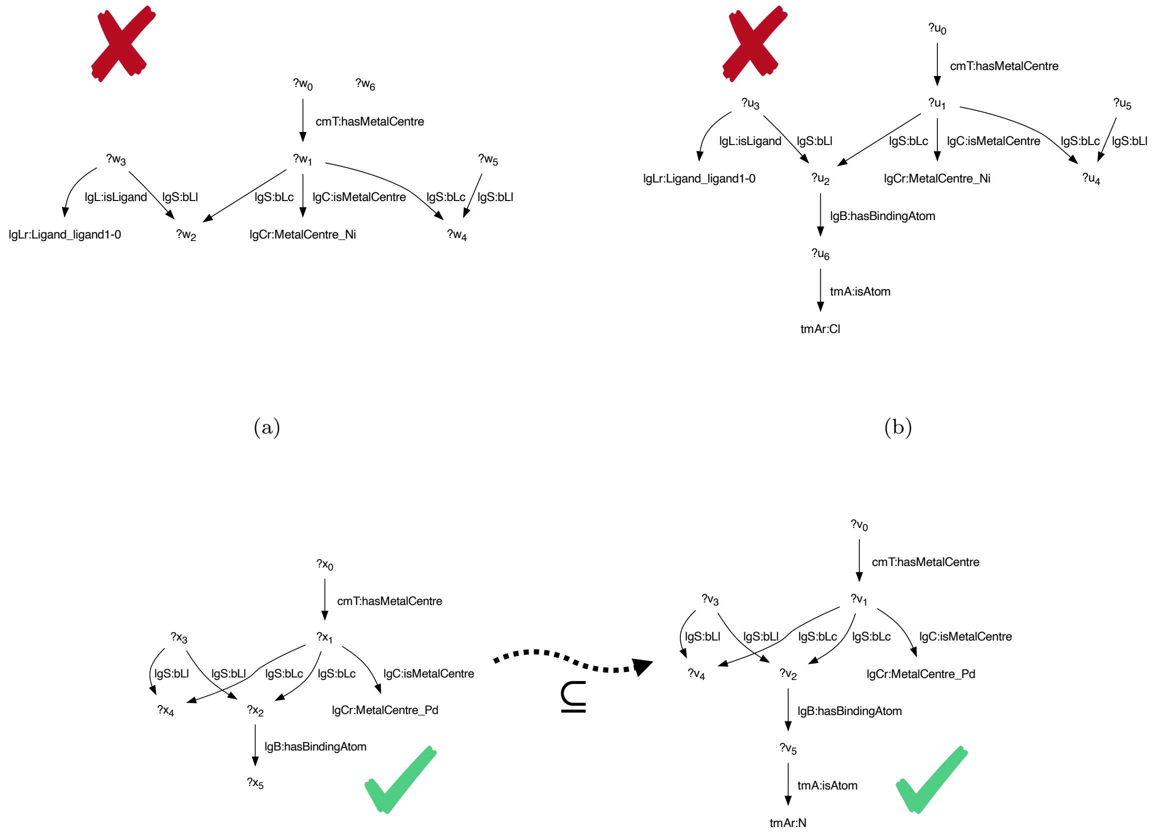

(a) (b)

(c)

Figure S8: (a) An example of an uninteresting graph pattern: it doesn't specify any binding atom (specifying a ligand alone is not enough for interestingness). (b) An example of an uninteresting graph pattern: even though it prescribes a binding atom (?u_6), whose identity is also specified as `tmAr:Cl`, this belongs to a ligand (?u_3) whose identity is also explicit (`lgLr:Ligand_ligand1-0`), thus making the information redundant. (c) An example of two interesting graph patterns: the pattern on the right indicates a binding atom (?v_5, which is an instance of `tmAr:N`) in a non-redundant way (the identity of its ligand, ?v_3 is unknown); the pattern on the left is a subpattern of (and hence dominates) the pattern on the right, which is interesting, hence it is interesting as well (even though it fails to specify the identity of the binding atom in ?x_5, which would have otherwise made it uninteresting).

Due to the combinatorial explosion of the number of possible groundings of the variables in a pattern, it may happen that the columns of $\widetilde{X}$ possess largely different scales (see Figure S9). For this reason, the actual elementary feature matrix X is defined as a binarised version

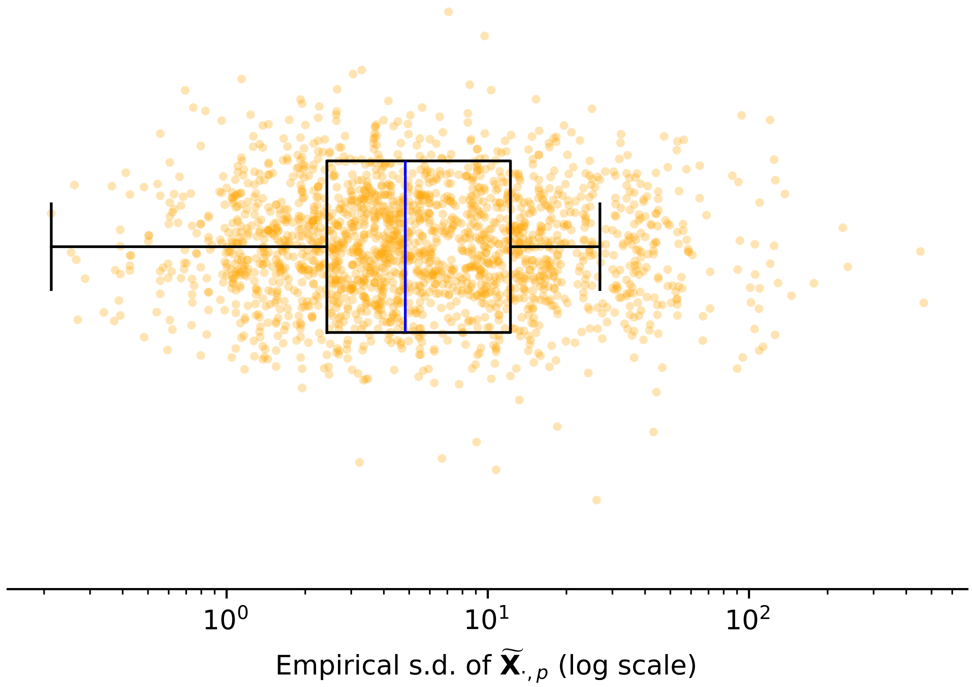

Figure S9: The boxplot of the empirical standard deviation (in log scale) of the columns of the elementary feature matrix $\widetilde{X}$ computed from the *earlyTM* dataset selection. Individual values are superimposed in the form of a scatterplot (a random vertical jitter has been added for readability).

of $\widetilde{X}$, where

$$X_{G,p} = \begin{cases} 1 & \text{if } \widetilde{X}_{G,p} > 0 \\ 0 & \text{otherwise} \end{cases} . \tag{S3}$$

S4.3 Clustering Into Families of Substructures

A graph pattern is forced to encode exactly one type of structure, up to the uncertainty eventually granted by the variables, and therefore any "naive" pattern mining procedure (as per Section S4.1.1) will likely capture structures which are way more specific than the desired fundamental behaviours. For example, in Figure S7, panel (a), it may be that specifying that N participates in a ligand-Pd bond is just a form of "noise", whereas the truly relevant information is the bidentate nature of the ligand. One way to address the issue is to employ a clustering algorithm to identify families of patterns that are similar according to some appropriate criterion. In this way, it is possible to capture the most meaningful substructure

types, while also effectively reducing the dimensionality of the feature matrix X introduced above.

The clustering method employed in this work falls within the category of hierarchical agglomerative clustering.[13] These methods proceed by progressively agglomerating items together, until all items have been merged into a single cluster or another stopping criterion is reached. Agglomeration is performed, at each step, by merging the two clusters that are closest to each other, according to a prespecified metric. Usually, a similarity (or dissimilarity) metric s between pairs of objects is employed, together with a so called *linkage criterion*, that specifies how to extend s to groups of objects. The chosen criterion, here, is the *average linkage* criterion, or *UPGMA (Unweighted Pair-Group Method using Arithmetic averages)*. This amounts to

$$s(A,B) = \frac{1}{|A|\cdot|B|}\sum_{\substack{p\in A\\ q\in B}} s(p,q), \tag{S4}$$

for each pair of clusters A and B. The linkage criterion also allows us to define a convenient stopping criterion. By specifying a threshold δ, we can terminate the procedure when all the pairwise similarities between clusters fall beneath δ. We determine the optimal value of δ among a set of candidates Δ by maximising the Silhouette coefficient[16] of the resulting clustering, under the constraint that there must be at least $M^{\mathcal{C}}_{\min}$ clusters[V].

As for the similarity metric s, we consider 2 candidate families of metrics and, for each family, we explore two possible configurations, giving in total 4 possible ways of computing s. In the main document we only considered one of these configurations, based on considerations that we make explicit in Section S11.1 For reasons of readability, we introduce here a brief description of each configuration, leaving the formal definitions for Section S9.

1. Cosine similarity: given two vectors in a d-dimensional space, the cosine similarity is

[V] This is enforced in order to reduce the possibility of mistakenly aggregating together patterns which represent different chemical modalities.

defined as the cosine of the angle between them. In order to use this similarity in the context of graph clustering, it is necessary to define a vector representation of a graph pattern p:

   (a) proxy vectors ($s_{\cos;\,p}$): under the assumption that similar patterns will match a similar set of graphs, the similarity between two patterns p and q can be computed as the cosine similarity between the columns $\boldsymbol{X}_{\cdot,p}$ and $\boldsymbol{X}_{\cdot,q}$ of the feature matrix X introduced in Section S4.2;

   (b) semantically-informed feature vectors ($s_{\cos;\,s}$): as DLGs, graph patterns can also be interpreted as sets of terms, other than sets of triples, hence it is possible to assign a weight to each term using the term frequency inverse document frequency (tf-idf)[17] scheme, allowing for the definition of a feature vector where each entry is indexed by a term and it evaluates to its tf-idf weight.

2. DLG similarity by Champin and Solnon (2003):[18] this metric is closely related to the graph edit distance[19] and it uses multivalent mappings between the nodes of two graphs to define an "overlap" or "intersection" of the graphs. If a weighting scheme for the terms in the graphs is available, a candidate similarity value can be obtained as the ratio between the total weight in the overlap and the total weight in the two graphs. The final similarity is computed as the highest candidate similarity value achievable in this way. The choice of the weighting scheme greatly affects the results:

   (a) naive weights ($s_{\mathrm{DLG};\,n}$): a constant weight ω_1 is assigned to labels which specify a precise chemical identity (e.g., `tmAr:C`, `tmAr:Pt`, ...) and a constant weight $\omega_0 < \omega_1$ is assigned to non-specific labels (e.g. `tmA:Atom`, `lgC:MetalCentre`, ...);

   (b) learned weights ($s_{\mathrm{DLG};\,l}$): a more advanced weighting scheme is inferred from the semantic content of $\mathcal{P}$ using the principles of the tf-idf scheme (see Section S9).

The behaviour of these 4 similarity metrics is investigated via a pairplot reported in Figure S10.

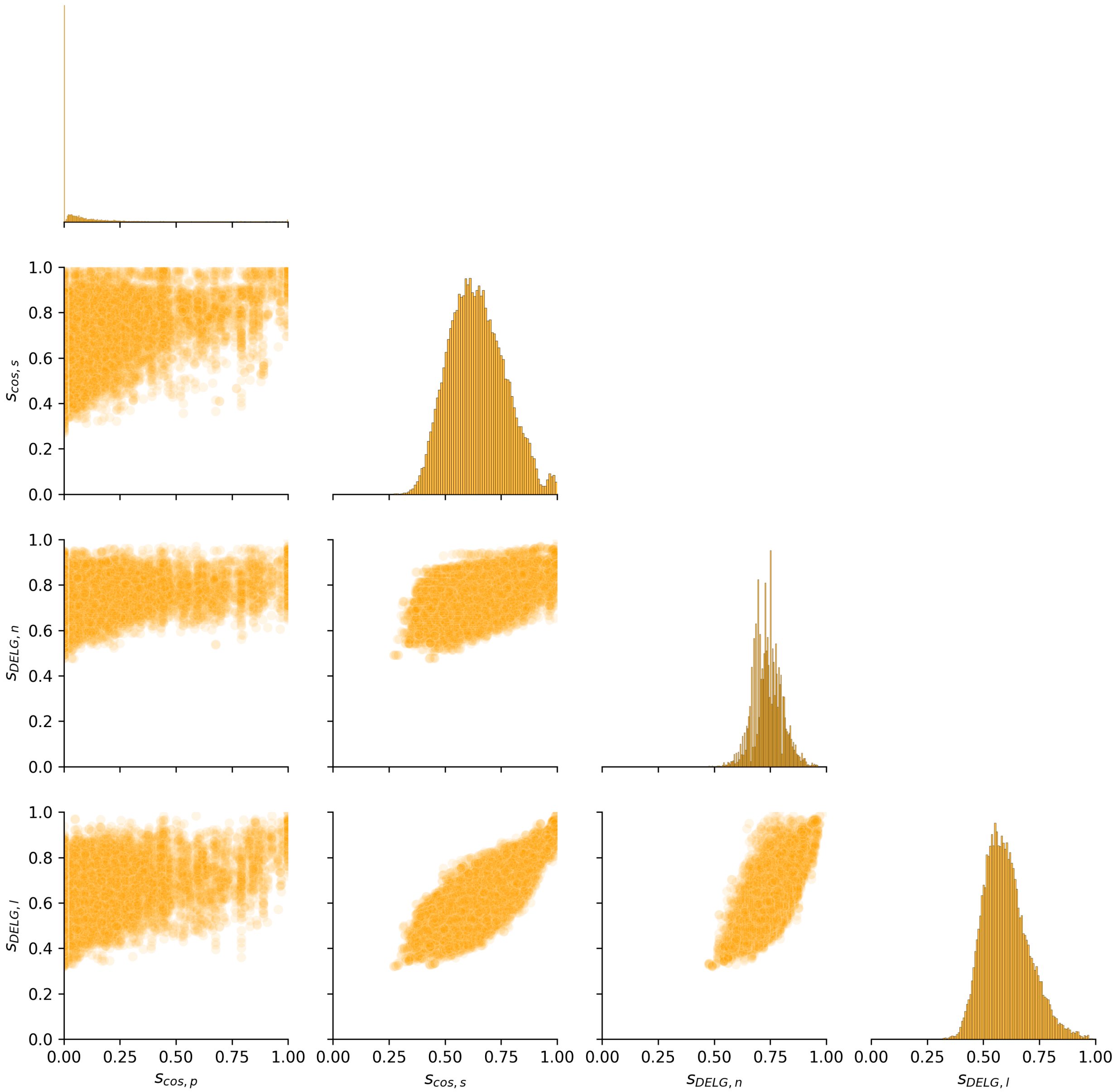

Figure S10: A pairplot of all the pairwise similarities among a random population of 300 patterns, sampled from the frequent patterns mined from the *earlyTM* dataset selection, for all the possible configurations of s. The plots on the diagonal of the grid are histograms of the similarity values computed for a given configuration, whereas the remaining plots are correlation plots.

S4.4 Aggregated Graph Features

Once a clustering $\mathcal{C}$ of the given set of patterns is obtained, it is possible to reduce the dimensionality of the feature matrix X and define a new set of features. If $\mathcal{C} = \{C_1, \ldots, C_P\}$, with $C_i \cap C_j = \emptyset$ for $i \neq j$, we can obtain a new feature matrix $Y \in \{0,1\}^{|\mathcal{G}|\times|\mathcal{C}|}$ by specifying an aggregation function a and then computing

$$Y_{G,C_i} = a(\boldsymbol{X}_{G,C_i}, C_i), \tag{S5}$$

where $\boldsymbol{X}_{G,C_i} = (X_{G,p})_{p\in C_i}$ (i.e., it is a view of the row vector $\boldsymbol{X}_{G,\cdot}$). In this work we consider two different aggregation functions:

1. $a_{\max}(\boldsymbol{X}_{G,C_i}, C_i) := \max\{X_{G,p} \mid p \in C_i\}$, which implies that

$$Y_{G,C_i} = \begin{cases} 1 & \text{if } \exists\, p \in C_i \text{ such that } \Omega_{G,p} \neq \emptyset \\ 0 & \text{otherwise} \end{cases}; \tag{S6}$$

2. $a_{\text{median}}(\boldsymbol{X}_{G,C_i}, C_i) := X_{G,p_{(C_i)}}$, where $p_{(C_i)} \in C_i$ is the *set median graph* of C_i,[20] i.e.

$$p_{(C_i)} = \operatorname*{argmax}_{p\in C_i} \sum_{p_j \in C_i} s(p, p_j). \tag{S7}$$

   In practice, this aggregation function has the effect of "collapsing" each cluster $C_i \in \mathcal{C}$ onto its median $p_{(C_i)}$.

Notice that each similarity metric described in Section S4.3 gives rise to a different clustering and, for each clustering, each aggregation function gives rise to different features. This means that, overall, there will be 8 versions of the feature matrix Y. As with the similarity metrics previously introduced, only one aggregation function is eventually presented in the main document. See Section S11.1 for further details.

S4.5 Probability-Based TMC Scoring Model

The rows of the feature matrix Y introduced in Section S4.4 can be interpreted as the observations of a numeric random vector $\boldsymbol{Y}_G$, derived as a deterministic function of a random TMC G, given the set of frequent patterns $\mathcal{P}$ and its clustering $\mathcal{C}$. In terms of defining a scoring function for G, this formulation becomes useful as it allows us to define $f(G) := \log \mathbb{P}(\boldsymbol{Y}_G \mid \mathcal{P}, \mathcal{C})$. The distribution of $\boldsymbol{Y}_G$ can therefore be interpreted as a way to assess the score of a TMC in the context of a TMC training population.

The combinatoric nature of pattern mining and the consequently elevated cardinality of $\mathcal{P}$ make $\boldsymbol{Y}_G$ a high-dimensional vector, even after clustering. This poses a serious challenge to the statistical learning problem at hand[21] and requires appropriate considerations. In this work we address the problem by modelling $\mathbb{P}(\boldsymbol{Y}_G \mid \mathcal{P}, \mathcal{C})$ using a *Bayesian Network* (BN),[14,15] a framework specifically designed to handle high-dimensional data.

In the BN model, the distribution of $\boldsymbol{Y}_G$ factorises according to a Directed Acyclic Graph B, whose nodes are the very clusters of $\mathcal{C}$ who also index the entries of the random vector. This factorisation relies on the concept of *parents* of a node $C \in \mathcal{C}$, i.e., the set of nodes that posses an outgoing edge pointing directly to C. This is denoted as $\mathrm{pa}(C) = \{C_j \in \mathcal{C} \mid C_j \to C \text{ in } B\}$. Intuitively, the parents are those variables that have a *direct* influence on C.[14] Building on this idea, a BN factorises the joint distribution of $\boldsymbol{Y}_G$ using the conditional distributions of $Y_{C_i} \mid \boldsymbol{Y}_{\mathrm{pa}(C_i)}, \mathcal{P}, \mathcal{C}$, with $\boldsymbol{Y}_{\mathrm{pa}(C_i)} = (Y_{C_j})_{C_j \in \mathrm{pa}(C_i)}$, i.e.,

$$\mathbb{P}(\boldsymbol{Y}_G \mid \mathcal{P}, \mathcal{C}) = \prod_{i=1}^{|\mathcal{C}|} \mathbb{P}(Y_{C_i} | \boldsymbol{Y}_{\mathrm{pa}(C_i)}, \mathcal{P}, \mathcal{C}). \tag{S8}$$

Since $\boldsymbol{Y}_G$ is a binary feature vector, the conditional distributions in (S8) can be modelled

using Bernoulli distributions, namely

$$\begin{aligned} Y_{C_i} \mid \boldsymbol{Y}_{\mathrm{pa}(C_i)}, \mathcal{P}, \mathcal{C} &\sim \mathrm{Bernoulli}(\theta_{\boldsymbol{Y}_{\mathrm{pa}(C_i)}}), \\ \mathbb{P}(Y_{C_i} = 1 | \boldsymbol{Y}_{\mathrm{pa}(C_i)}, \mathcal{P}, \mathcal{C}) &= \theta_{\boldsymbol{Y}_{\mathrm{pa}(C_i)}}. \end{aligned} \tag{S9}$$

Formula (S8) provides a series of benefits, the most evident of which is the factorisation of $\mathbb{P}(\boldsymbol{Y}_G \mid \mathcal{P}, \mathcal{C})$ into the product of simpler univariate distributions. Additionally, it is possible to naturally derive a set of conditional independence statements about $\boldsymbol{Y}_G$, specifically

$$Y_{C_i} \perp\!\!\!\perp \boldsymbol{Y}_{\mathrm{nd}(C_i)} \mid \boldsymbol{Y}_{\mathrm{pa}(C_i)}, \tag{S10}$$

where $\mathrm{nd}(C_i)$ is the set of the *non-descendents* of C_i, i.e., the set of all the clusters C_j such that there does not exist a directed path from C_i to C_j. These are directly inferrable from the topology of B, meaning that a BN also offers a way to graphically inspect and represent a probability distribution.

S4.5.1 Learning the DAG

The crucial point of training a BN is, of course, acquiring the graph B. There are several structure learning algorithm available[15] which can be used to obtain an estimate of B from observational data, possibly starting from a preliminary subgraph B_0, if such prior knowledge is available.

In this work we chose to employ the Hill-Climbing (HC) algorithm from the `bnlearn` package[22] in `R`. This is a greedy algorithm that searches for the graph B that optimises the *Bayesian Information Criterion*

$$\mathrm{BIC}(B) = \sum_{G \in \mathcal{G}_{train}} \ln \hat{\mathbb{P}}(\boldsymbol{y}_G \mid \mathcal{P}, \mathcal{C}) - \frac{\ln |\mathcal{G}_{\mathrm{train}}|}{2} F, \tag{S11}$$

where the first term is the maximised log-likelihood of the training data according to the model specified by (S8) and (S9) and the second is a penalty proportional to the number of free parameters in the model $F = \sum_{C_i \in \mathcal{C}} 2^{|\mathrm{pa}(C_i)|}$.[15] The optimisation is carried out in a sequence of steps, where each step corresponds to applying one operation, i.e. edge insertion, deletion, or removal, on the current estimate of B. We use prior information, in the form of the subgraph B_0, to provide a starting point to HC. This subgraph is derived by means of structural considerations on the patterns in $\mathcal{P}$, with respect of the notion of pattern domination introduced in Section S4.1.2.

By definition, if $p, q \in \mathcal{P}$ are such that q dominates p, it holds that, for any RDF graph G, if q does not match G, then p cannot match G either. In terms of the elementary graph-level feature matrix X defined in Section S4.2, this reads as: $X_{G,q} = 0$ implies $X_{G,p} = 0$. It is then evident that such patterns p and q have a direct influence on each other, in a similar sense in which a node experiences a direct influence from its parents in a BN. The matter is however made more complicated by the fact that the variables of interest in our model are not patterns, but clusters of patterns. As this work is intended as a mere example of the potential of our proposed data representation, we make here the simplifying assumption that the existence of a pair p and q as above, with patterns belonging to different clusters, implies an influential effect of one cluster on the other. Deciding on the direction of the effect, i.e., which node is the parent, is another delicate point, which we also choose not to discuss in detail. Since HC has the ability to reverse edge orientations, we simply choose a random orientation and leave the matter undecided.

Formally, then, we obtain B_0 by first defining an undirected graph $\tilde{B}_0$, whose nodes are the clusters in $\mathcal{C}$, and we draw an edge between $C_i, C_j \in \mathcal{C}$ if and only if there exists $p \in C_i, q \in C_j$ such that q dominates p. The directed graph B_0 is derived from $\tilde{B}_0$ by randomly orienting all edges in such a way that no directed cycles are formed.

As in Section S4.4, each version of the feature matrix Y, deriving from a different similarity metric-aggregation function pair, will produce a different BN, thus leading to 8 possible networks.

S4.5.2 Parameter Estimation

Estimating the parameters in (S9) is the final step in training the BN model. We employ a Bayesian approach, using the Dirichlet priors

$$\left(\theta_{\boldsymbol{Y}_{\mathrm{pa}(C_i)}},\, 1-\theta_{\boldsymbol{Y}_{\mathrm{pa}(C_i)}}\right) \sim \mathrm{Dirichlet}(\lambda_0,\, \lambda_0), \tag{S12}$$

which are equivalent to the Beta priors

$$\theta_{\boldsymbol{Y}_{\mathrm{pa}(C_i)}} \sim \mathrm{Beta}(\lambda_0,\, \lambda_0), \tag{S13}$$

where $\lambda_0 > 0$ and $\{\theta_{\boldsymbol{Y}_{\mathrm{pa}(C_i)}}\}_{\boldsymbol{Y}_{\mathrm{pa}(C_i)} \in \{0,\,1\}^{|\mathrm{pa}(C_i)|}}$ is the family of the parameters of the conditional distributions $Y_{C_i} \mid \boldsymbol{Y}_{\mathrm{pa}(C_i)}, \mathcal{P}, \mathcal{C}$ for all the possible parent states $\boldsymbol{Y}_{\mathrm{pa}(C_i)} \in \{0,\,1\}^{|\mathrm{pa}(C_i)|}$. The estimation is carried out using the `pgmpy` package in Python.[23]

S4.6 The Complete Training Phase

With the concepts introduced above, it is now possible to completely describe the steps of the training phase of the proposed experiment. This phase is divided in six fundamental steps, listed below. The aim is the isolation the relevant structural information, via preprocessing, pattern mining and clustering, and the fitting of the BN model.

1. Acquiring $\mathcal{G}$ from tmQM-RDF: given a TMC population of interest, the RDF subgraphs describing those TMCs are extracted from tmQM-RDF, by locating the nodes of the form `cmT:XXYYZZ`, where `XXYYZZ` is a CSD code, and by then following the outgoing edges. Since only structural information is of interest here, the extracted subgraphs

will only include the predicates listed in Table S6. See Section S3 for details on the nature of the populations described by $\mathcal{G}$ and the partition in $\mathcal{G}_{\text{train}}$ and $\mathcal{G}_{\text{test}}$.

2. Graph pattern mining: frequent patterns are mined from $\mathcal{G}_{\text{train}}$, using the algorithm in Section S4.1.1.

3. Pattern filtering: the mined patterns are filtered according to the principles described in Section S4.1.2.

4. Elementary feature computation: the elementary graph-level features described in Section S4.2 are computed.

5. Identification of structural families: frequent patterns are clustered together using the similarity-based clustering technique described in Section S4.3. This is repeated for each possible configuration of the similarity metric, as explained in Section S4.3.

6. Aggregated features computation: the elementary features are aggregated according to the identified structural families. This is repeated for each possible configuration of similarity metric and aggregation function, as explained in Section S4.4.

7. Statistical learning: the joint distribution of the graph features is estimated as a BN, accounting for statistical interactions (i.e., correlations) and structural relationships among the features (i.e., dominations). This is repeated for each possible configuration of similarity metric and aggregation function, as explained in Section S4.5.1.

S5 Technical Details About Frequent Pattern Mining

This section describes the idea behind the algorithm[VI] used to mine frequent patterns, as per the definition given in (S1).

[VI] This algorithm has been developed and implemented by B. E. and will be thoroughly addressed in a separate publication.

Table S6: The URIs of the only predicates allowed in frequent patterns. The predicates are grouped according to the structural element they primarily identify via their domain/range.

Identified structural element	URI
Metal centre	cmT:hasMetalCentre<br>lgC:isMetalCentre
Ligand	cmT:hasLigand<br>lgS:bLc<br>lgS:bLl<br>lgL:isLigand
Binding atom	lgB:hasBindingAtom<br>tmA:isAtom

In what follows, let $\mathcal{G}_{\text{train}}$ be a dataset of RDF graphs and let $\alpha \in \mathbb{N}$ be the threshold for pattern frequency. The ultimate goal is to mine a set $\widetilde{\mathcal{P}}$ of frequent graph patterns. The algorithm hereby described proceeds hierarchically, by progressively expanding patterns of a given size (i.e., number of triples) by one additional triple (i.e., by adding a pattern of size 1) at a time.

S5.1 Notation

We briefly recall here that we define a DLG as a subset $G \subseteq \mathcal{T}^3$, where $\mathcal{T}$ is a (possibly infinite) set of terms, eventually with the restrictions imposed by the RDF syntax described in Section S1.1. Using this notation, merging two DLGs G_1, G_2 is achieved simply via the usual set union operation $G_1 \cup G_2$, whereas the subgraph relation coincides with the subset relation $G_1 \subseteq G_2$.

A graph pattern, on the other hand, is a DLG defined as a subset of $(\mathcal{T} \cup \mathcal{V})^3$, where $\mathcal{V}$ is an infinite set of symbols called variables. Given a pattern p, we denote with $\mathcal{T}_p$ and $\mathcal{V}_p$ the set of terms and variables, respectively, that appear in p. Given a function $m : \mathcal{T} \cup \mathcal{V} \to \mathcal{T}$, we denote with $m(p)$ the DLG obtained by applying m to every element of every triple of p. This work only considers patterns that admit variables either in subject or object position (hence never in predicate position). For simplicity, we will still write $p \subseteq (\mathcal{T} \cup \mathcal{V})^3$ and consider this constraint to be enforced implicitly.

Finally, given a DLG G and a pattern p, a match of p against G under the no-repeated-anything graph pattern evaluation semantics is defined as an injective function $\mu : \mathcal{T}_p \cup \mathcal{V}_p \to \mathcal{T}_G$ such that $\mu(t) = t$ for every $t \in \mathcal{T}_G$ and $\mu(p) \subseteq G$. The set of all such functions is denoted as $\Omega_{G,p}$.

S5.2 Preliminary Definitions

Let $\widetilde{\mathcal{P}}_i$ be the set of frequent patterns of size i. In particular, patterns of $\widetilde{\mathcal{P}}_1$, i.e., patterns made of a single triple, are called *triple patterns*.

Consider now two patterns $p_a \in \widetilde{\mathcal{P}}_i$, for some $i \geq 1$, and $p_b \in \widetilde{\mathcal{P}}_1$. The set of the *possible extensions of* p_a *given* p_b, denoted as $\mathcal{E}(p_a \mid p_b)$ is defined as the set of all the injective mappings $m : \mathcal{V}_{p_b} \to \mathcal{V}$ such that $m(p_b) \nsubseteq p_a$ and there exists at least one variable $v \in \mathcal{V}_{p_b}$ such that $m(v) \in \mathcal{V}_{p_a}$ (in other words, $\mathcal{E}(p_a \mid p_b)$ is the set of all the possible relabelling of the variables in p_b that ensure that the transformed triple pattern is not a triple already present in p_a and that the extended pattern $p_a \cup m(p_b)$ is connected). Two mappings that only differ in how they label elements that are not mapped into $\mathcal{V}_{p_a}$ are considered equivalent.

Given two matches $\mu_a \in \Omega_{G,p_a}, \mu_b \in \Omega_{G,p_b}$, for some $G \in \mathcal{G}$, they are said to be *compatible*, given a mapping $m \in \mathcal{E}(p_a \mid p_b)$ if the following conditions hold:

1. $\mu_a(\mathcal{V}_{p_a}) \cap \mathcal{T}_{p_b} = \emptyset$;

2. $\mu_b(\mathcal{V}_{p_b}) \cap \mathcal{T}_{p_a} = \emptyset$;

3. for any $v_a \in \mathcal{V}_{p_a}, v_b \in \mathcal{V}_{p_b}$, $m(v_b) = v_a$, if and only if $\mu_a(v_a) = \mu_b(v_b)$.

Given two compatible matches $\mu_a \in \Omega_{G,p_a}, \mu_b \in \Omega_{G,p_b}$, given $m \in \mathcal{E}(p_a \mid p_b)$, we define the *extension of* μ_a *by* μ_b *via* m as the function $\mu_a \cup m(\mu_b) : \mathcal{T}_{p_a} \cup \mathcal{V}_{p_a} \cup \mathcal{T}_{p_b} \cup m(\mathcal{V}_{p_b}) \to \mathcal{T}$ given

by

$$(\mu_a \cup m(\mu_b))\,(x) := \begin{cases} x & \text{if } x \in \mathcal{T}_{p_a} \cup \mathcal{T}_{p_b} \\ \mu_a(x) & \text{if } x \in \mathcal{V}_{p_a} \\ \mu_b(m^{-1}(x)) & \text{otherwise} \end{cases} . \tag{S14}$$

The expression $m^{-1}(x)$ is a slight abuse of notation as we are implicitly considering the codomain of m to be restricted to $m(\mathcal{V}_{p_b})$. Notice also how the compatibility of μ_a and μ_b ensures that $\mu_a \cup m(\mu_b)$ is well defined and that $\mu_a \cup m(\mu_b) \in \Omega_{G,p_a \cup m(p_b)}$.

Two patterns $p_a, p_b \in \widetilde{\mathcal{P}}_i$ are said to be *isomorphic* if there exists a bijective function $m : \mathcal{V}_{p_a} \cup \mathcal{T}_{p_a} \to \mathcal{V}_{p_b} \cup \mathcal{T}_{p_b}$ such that $m(t) = t$ for each $t \in \mathcal{T}_{p_a}$ and $m(p_a) = p_b$.

S5.3 Frequent Pattern Mining Algorithm

The outline of the pattern mining algorithm used in this work is described in this section. As anticipated, the basic idea is to progressively extend patterns of size i by adding a single triple pattern. Extension of a pattern $p_a \in \widetilde{\mathcal{P}}_i$ with a triple pattern $p_b \in \widetilde{\mathcal{P}}_1$ is performed by considering the possible extensions $m \in \mathcal{E}(p_a \mid p_b)$ to form extended patterns $p_a \cup m(p_b)$. The matches of these extended patterns against graphs $G \in \mathcal{G}$ are immediately computed by considering all the compatible matches $\mu_a \in \Omega_{G,p_a}, \mu_b \in \Omega_{G,p_b}$ and by computing the extension $\mu_a \cup m(\mu_b)$. The mining procedure starts from a single triple pattern $p_0 \in (\mathcal{T} \cup \mathcal{V})^3$, also called a *seed pattern*, which is extended to obtain larger graph patterns.

As the task can be particularly demanding from a computational point of view, a random sampling procedure is introduced in order to use only a fraction of the possible extensions of a pattern. Let then $(\pi_i)_{i \geq 2}$ be a sequence of probabilities. When considering a pattern $p_a \in \widetilde{\mathcal{P}}_i$, to be extended with $p_b \in \widetilde{\mathcal{P}}_1$ to form a pattern of size $i + 1$, each extension $m \in \mathcal{E}(p_a \mid p_b)$ has a probability π_{i+1} of being *discarded*.

In what follows, we then denote with $\hat{\mathcal{E}}(p_a \mid p_b)$ the set of accepted extension. Similarly,

we denote with $\hat{\mathcal{P}}_i$ the set of *mined* patterns of size i that will be part of the output of the algorithm (these set are subsets of the collections $\widetilde{\mathcal{P}}_i$ of *all* the frequent patterns in the dataset).

The full set of inputs of the algorithm is then the following: $\mathcal{G}_{train}$, a set of training DLGs from which frequent patterns are to be mined; $\alpha \in \mathbb{N}$, the threshold used to determine if a pattern if frequent; p_0, a seed triple pattern; and $(\pi_i)_{i\geq 2}$, the sequence of rejection probabilities, a maximum pattern size $M_{\max}$.

1. Compute the set $\widetilde{\mathcal{P}}_1$ of frequent triple patterns that will be used to extend the patterns:
   (a) Extract all the triples from all the graphs of $\mathcal{G}_{train}$ and replace either the subject or the object (or both) with a variable to obtain an initial set of triple patterns[VII] (for each triple pattern, keep track of the graph G from which it was extracted and the original triple).
   (b) For each triple pattern p so obtained, populate the sets $\Omega_{G,p}$ with the matches that replace the new variables with the original terms before the substitution.
   (c) Retain only the triple patterns that match at least α different graphs and use them to populate $\widetilde{\mathcal{P}}_1$.
2. Initialise $\hat{\mathcal{P}}_1 \leftarrow \{p_0\}$.
3. Initialise $i \leftarrow 1$.
4. Repeat the following as long as $\hat{\mathcal{P}}_i \neq \emptyset$ and $i \leq M_{\max}$:
   (a) Initialise $\hat{\mathcal{P}}_{i+1} \leftarrow \emptyset$.
   (b) For each $p_a \in \hat{\mathcal{P}}_i, p_b \in \widetilde{\mathcal{P}}_1$:
      i. Compute $\hat{\mathcal{E}}(p_a \mid p_b)$:

[VII]As mentioned, we do not consider here triple patterns that have a variable in predicate position. Similarly, in this phase it is also possible to implement other forms of constraints regarding variable placement or predicate blacklisting.

(A) Initialise $\hat{\mathcal{E}}(p_a \mid p_b) \leftarrow \emptyset$.

(B) For each $\mathcal{V}'_{p_a} \subseteq \mathcal{V}_{p_a}, \mathcal{V}'_{p_b} \subseteq \mathcal{V}_{p_b}$, with $|\mathcal{V}'_{p_a}| = |\mathcal{V}'_{p_b}| > 0$:

I. For each injective mapping $m_0 : \mathcal{V}'_{p_b} \to \mathcal{V}'_{p_a}$:

a. Extend m_0 to $m : \mathcal{V}_{p_b} \to \mathcal{V}$ by assigning to each variable of $\mathcal{V}_{p_b} \setminus \mathcal{V}'_{p_b}$ a symbol from $\mathcal{V} \setminus \mathcal{V}_{p_a}$.

b. If $m(p_b) \nsubseteq p_a$, with probability $1 - \pi_{i+1}$, update $\hat{\mathcal{E}}(p_a \mid p_b) \leftarrow \hat{\mathcal{E}}(p_a \mid p_b) \cup \{m\}$.

ii. For each $m \in \hat{\mathcal{E}}(p_a \mid p_b)$:

(A) Let $p'_a = p_a \cup m(p_b)$.

(B) For each $G \in \mathcal{G}_{\text{train}}$:

I. For each $\mu_a \in \Omega_{G,p_a}, \mu_b \in \Omega_{G,p_b}$ such that μ_a and μ_b are compatible, update $\Omega_{G,p'_a} \leftarrow \Omega_{G,p'_a} \cup \{\mu_a \cup m(\mu_b)\}$.

(C) If p'_a is frequent and not isomorphic to any pattern in $\hat{\mathcal{P}}_{i+1}$, update $\hat{\mathcal{P}}_{i+1} \leftarrow \hat{\mathcal{P}}_{i+1} \cup \{p'_a\}$.

(c) Update $i \leftarrow i + 1$.

5. Return $\hat{\mathcal{P}}_1 \cup \hat{\mathcal{P}}_2 \cup \cdots \cup \hat{\mathcal{P}}_{M_{\max}}$.

S6 Isolating Interesting Patterns

Let $\widetilde{\mathcal{P}}$ be the set of frequent patterns produced by the algorithm in Section S5. We recall that, as per Section S4.1.2, we consider a pattern $p \in \widetilde{\mathcal{P}}$ to be interesting if it specifies the existence and the identity of at least one binding atom, in a non redundant way.[VIII] Moreover, we say that a pattern $q \in \widetilde{\mathcal{P}}$ dominates a pattern p if, for any RDF graph G, $|\Omega_{G,q}| = 0$ implies $|\Omega_{G,p}| = 0$.

[VIII] We recall that a specified binding atom is considered non-redundant if the pattern does not also specify the chemical identity of the ligand the atom belongs to.

Our aim is then that of computing a subset $\mathcal{P} \subseteq \widetilde{\mathcal{P}}$ made of patterns that are either interesting, or dominate an interesting pattern. Using the notation introduced in Section S5, we consider the decomposition $\widetilde{\mathcal{P}} = \hat{\mathcal{P}}_1 \cup \cdots \cup \hat{\mathcal{P}}_{M_{\max}}$. For simplicity, we construct $\mathcal{P}$ using only patterns from $\hat{\mathcal{P}}_{M_{\min}} \cup \cdots \cup \hat{\mathcal{P}}_{M_{\max}}$, where $M_{\min} \in \mathbb{N}$ is a user-defined minimum pattern size.

The construction of $\mathcal{P}$ proceeds as follows.

1. Initialise $\mathcal{P} \leftarrow \emptyset$.
2. Initialise $\mathcal{R} \leftarrow \mathcal{P}$.
3. Initialise $i \leftarrow M_{\max}$.
4. Repeat the following as long as $i \geq M_{\min}$:
   (a) Update $\mathcal{P} \leftarrow \mathcal{P} \cup \{p \in \hat{\mathcal{P}}_i \mid p \text{ is interesting}\}$.
   (b) Update $\mathcal{P} \leftarrow \mathcal{P} \cup \{q \in \hat{\mathcal{P}}_i \mid \exists p \in \mathcal{R} : q \text{ dominates } p\}$.
   (c) Update $\mathcal{R} \leftarrow \mathcal{P} \setminus \mathcal{R}$.
   (d) Update $i \leftarrow i - 1$.
5. Return $\mathcal{P}$.

S7 Implementation of Pattern Matching

In practice, the matches of a pattern p against a graph G can be easily computed using the SPARQL query language.[12]

As SPARQL is a graph pattern matching-based query language,[12] a graph pattern p as introduced in Section S4.1 can be naturally transformed into a query. In particular, the set of triples of p will form the body of `WHERE` clause of the query. The no-repeated-anything graph pattern evaluation semantics is also easily implemented by including a `FILTER` instruction that prevents any variable to match terms already present in the pattern and any two variables to

match the same term.

Once a pattern is written into a SPARQL query, we employ the `R` package `virtuoso`[24] as a convenient interface to SPARQL.

S8 On the Domination Relationship Between Graph Patterns

Given two patterns p and q, we recall that we say that q dominates p if, for any RDF graph G, $|\Omega_{G,q}| = 0$ implies $|\Omega_{G,p}| = 0$. Equivalently, $|\Omega_{G,p}| > 0$ implies $|\Omega_{G,q}| > 0$.

Proposition S8.1. *Let p, q be two frequent patterns given a graph dataset $\mathcal{G}$. Then the following statements are equivalent:*

1. *q dominates p.*
2. *There exists an injective mapping $\mu : \mathcal{T}_q \cup \mathcal{V}_q \to \mathcal{T}_p \cup \mathcal{V}_p$ such that $\mu(t) = t$ for each term $t \in \mathcal{T}_q$ and $\mu(q)$ is a subgraph of p.*

Proof. First, consider the case in which q dominates p. Construct then an RDF graph G by grounding p using terms from $\mathcal{T} \setminus \mathcal{T}_q$, so that $\mathcal{T}_G = \mathcal{T}_p \cup \mathcal{T}_0,\ \mathcal{T}_q \cap \mathcal{T}_0 = \emptyset$. Trivially, there exists a mapping $\mu_p \in \Omega_{G,p}$ and, in particular, p and G are isomorphic via μ_p. It also holds that

$$\forall t \in \mathcal{T}_p,\ \mu_p^{-1}(t) = t, \tag{S15}$$

$$\forall t \in \mathcal{T}_0,\ \mu_p^{-1}(t) \in \mathcal{V}_p. \tag{S16}$$

But now, since q dominates p, there must also be a mapping $\mu_q \in \Omega_{G,q}$. By the construction of G, it must then be that $\mathcal{T}_q \subseteq \mathcal{T}_p$ and therefore

$$\forall t \in \mathcal{T}_q,\ \ \mu_p^{-1}(\mu_q(t)) = \mu_q(t) = t. \tag{S17}$$

Moreover, since $\mu_q(q)$ is a subgraph of G, which is isomorphic to p via μ_p, it must follow that $\mu_p^{-1}(\mu_q(q))$ is a subgraph of p. Finally, since both μ_p and μ_q are injective, so is the mapping $\mu_p^{-1} \circ \mu_q$, which then satisfies all the required properties.

Suppose now that there exists a mapping μ as described in statement (2) and let G be a graph such that there exists $\mu_p \in \Omega_{G,p}$. It is clear that the mapping $\mu_p \circ \mu : \mathcal{T}_q \cup \mathcal{V}_q \to \mathcal{T}_G$ is injective and that $\mu_p(\mu(q))$ is a subgraph of G. Moreover,

$$\forall t \in \mathcal{T}_q,\ \mu_p(\mu(t)) = \mu_p(t) = t, \tag{S18}$$

where the last equality is based on the fact that, by the properties of μ, it necessarily follows that $\mathcal{T}_q \subseteq \mathcal{T}_p$. We can then conclude that $\mu_p \circ \mu \in \Omega_{G,q}$. □

This proposition offers a convenient way of checking whether two patterns p and q are related via domination. In fact, condition (2) can be equivalently written as follows:

3. Let G_p be an RDF graph built by grounding p using terms from a set $\mathcal{T}_0$, $\mathcal{T}_0 \cap (\mathcal{T}_p \cup \mathcal{T}_q) = \emptyset$. Then $|\Omega_{G_p,q}| > 0$.

It follows, then, that pattern domination is equivalent to pattern matching. We provide a concrete example of this principle in Example S8.1

Example S8.1. Let p be the pattern shown in Figure S11, panel (a). This pattern will match a TMC G if and only if this TMC has Pd as its metal centre and a bidentate ligand which binds to the centre with at least one N atom. Consider now the triple pattern $q = \{(?w0, \texttt{tmA:isAtom}, ?w1)\}$.

If μ_p is a match of p against G, then a match μ_q of q against G is immediately obtained as

$$w_0 \overset{\mu_q}{\longmapsto} \mu_p(v_5) \tag{S19}$$

$$w_1 \longmapsto \texttt{tmAr:N}. \tag{S20}$$

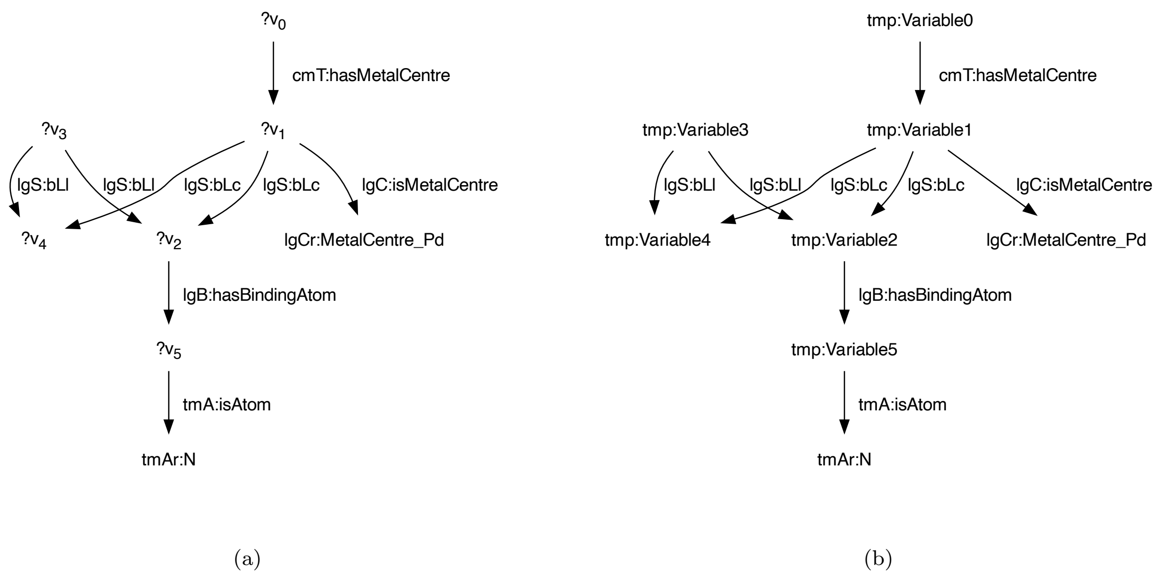

Figure S11: (a) An example of a graph pattern. (b) An example of a graph matched by the graph pattern, obtained by grounding the variables in the pattern itself.

Since p matches G, it must be that $(\mu_p(v_5), \texttt{tmA:isAtom}, \texttt{tmAr:N}) \in G$, which implies that $\mu_q(q) \subseteq G$. It follows then that q dominates p. This can be equivalently written in terms of the composition $\mu_p^{-1} \circ \mu_q$, as

$$w_0 \xmapsto{\mu_p^{-1} \circ \mu_q} v_5 \tag{S21}$$

$$w_1 \longmapsto \texttt{tmAr:N}, \tag{S22}$$

which provides us with the mapping μ whose existence is assured by Proposition S8.1.

Suppose now that, instead of the considerations above, we approach the situation by considering a graph G obtained by grounding p, for example the graph shown in

Figure S11, panel (b). Then, a match μ'_q of q against this G is simply given by

$$w_0 \overset{\mu'_q}{\longmapsto} \texttt{tmp:Variable5} \tag{S23}$$

$$w_1 \longmapsto \texttt{tmAr:N}. \tag{S24}$$

But an equally immediate match μ'_p of p is now given by

$$v_i \overset{\mu'_p}{\longmapsto} \texttt{tmp:Variable}i \qquad i = 0, \ldots, 5 \tag{S25}$$

and this match is, in particular, an invertible function when its codomain is restricted to $\{\texttt{tmp:Variable}i \mid i = 0, \ldots, 5\}$. We can then define the mapping $\mu := (\mu'_p)^{-1} \circ \mu_q$ which has the following action:

$$w_0 \overset{\mu'_q}{\longmapsto} \texttt{tmp:Variable5} \xmapsto{(\mu'_p)^{-1}} v_5 \tag{S26}$$

$$\texttt{?w1} \longmapsto \texttt{tmAr:N} \longmapsto \texttt{tmAr:N}. \tag{S27}$$

By Proposition S8.1, the existence of this mapping ensures that q dominates p. We have then been able to verify the validity of the domination relationship simply by virtue of our capability of computing pattern matches. △

S9 Similarity Metrics

In order to properly discuss the metrics introduced in Section S4.3, it is useful to introduce a more rigorous definition of a DLG. For the remainder of this section, a DLG will be intended to be a triple $G = (V, E, \pi)$, where V is the set of nodes, $E \subseteq V \times V$ is the set of (directed) edges and $\pi : V \cup E \to \mathcal{T}$ is a labelling function. The notation $\pi(v) = t$, for $v \in V$, means that the node v is labelled with the term $t \in \mathcal{T}$, whereas $\pi(v_s, v_o) = t_p$ signifies that the edge which has tail with label $\pi(v_s)$ (the subject) and head with label $\pi(v_o)$ (the object) is given

the label t_p (the predicate). It is straightforward to move from the definition given in Section S1.1.1 to the one just stated. If $G_{\text{set}} \subseteq \mathcal{T}^3$ is a set of triples, we can obtain an equivalent graph G by defining $V = \{t \in \mathcal{T} \mid \exists t_1, t_2 \in \mathcal{T} : (t,\, t_1,\, t_2) \in G_{\text{set}} \;\vee\; (t_1,\, t_2,\, t) \in G_{\text{set}}\}$, $E = \{(t_s,\, t_o) \in V \mid \exists t_p \in \mathcal{T} : (t_s,\, t_p,\, t_o) \in G_{\text{set}}\}$ and π such that $\pi(v) = v$ for every $v \in V \subseteq \mathcal{T}$ and $\pi(t_s, t_o) = t_p$ whenever $(t_s, t_p, t_o) \in G_{set}$.[IX]

In cases in which there may be ambiguity as to which DLG we refer to, we shall specify that by denoting the node set, the edge set and the labelling function as V_G, E_G and π_G respectively.

When DLGs are expressed using the RDF language, the restrictions described in Section S1.1.1 naturally apply.

S9.1 Cosine Similarity

The cosine similarity is a similarity metric defined over vectors of $\mathbb{R}^d$. Given $\boldsymbol{x}, \boldsymbol{y} \in \mathbb{R}^d$, their cosine similarity is

$$s_{\cos}(\boldsymbol{x},\, \boldsymbol{y}) = \frac{\boldsymbol{x} \cdot \boldsymbol{y}}{\|\boldsymbol{x}\|_2 \|\boldsymbol{y}\|_2}, \tag{S28}$$

where $\|\cdot\|_2$ is the Euclidean norm and $\cdot$ is the usual dot product in $\mathbb{R}^d$.

Now, since the objects between which a similarity is to be computed are graph patterns (and therefore labelled graphs, not vectors), in order to use the cosine similarity it is necessary to introduce appropriate feature vectors.

S9.1.1 Proxy Feature Vectors

The easiest way to address the similarity problem is probably to exploit the columns of the (binarised) feature matrix X introduced in Section S4.2 as proxy for the actual patterns, under the assumption that similar patterns will match a similar set of graphs. Using this approach, the similarity between the patterns p and q, measured as the cosine similarity

[IX] Remember that this work only considers sets of triples such that the corresponding graph has at most one edge between any two nodes and at most one node per term.

between the corresponding columns of X, is

$$s_{\cos;\,p}(p,q) := s_{\cos}(\boldsymbol{X}_{\cdot,p}, \boldsymbol{X}_{\cdot,q}) = \frac{\boldsymbol{X}_{\cdot,p} \cdot \boldsymbol{X}_{\cdot,q}}{\|\boldsymbol{X}_{\cdot,p}\|_2 \|\boldsymbol{X}_{\cdot,q}\|_2}, \tag{S29}$$

where $\|\cdot\|_2$ is the Euclidean norm and $\cdot$ is the usual dot product in $\mathbb{R}^{|\mathcal{G}|}$.

S9.1.2 Semantic Feature Vectors

It is not hard to imagine why the feature vectors introduced above may be unsatisfactory. First of all, the very idea of relying on X instead of directly employing the semantic information within the patterns means that we are not using all the available information. Secondly, there may be other reasons, apart from structural similarity, for which two patterns may share a similar matching profile. For instance, if two patterns encode two different structures that are strongly positively correlated, then the two patterns will tend to match a similar set of graphs, even though they may be remarkably different. This implies that statistically significant information may be masked within clusters.

Since the task is to identify families of patterns that encode similar information, an ideal metric should then exploit the semantic content of the patterns themselves, as this is the most complete source of knowledge about the information encoded therein. In this context, "semantic content" could be interpreted as the set of RDFS classes that are present within a pattern. Notice that an item can either receive an explicit class assignment via a predicate that is a subproperty of `rdf:type` (e.g. `tmA:isAtom`) or its class can be inferred from the TBox in Figure S3 in the main text and, if multiple classes can be determined, within tmQM-RDF it is always possible to determine a most specific class with respect to the subclass relation. This concept can be effectively synthesised by introducing a "compressed" representation $\tilde{p}$ of a pattern p, which amounts to a labelled graph derived from p by deleting all triples in which the predicate is a subproperty of `rdf:type` and by labelling all the

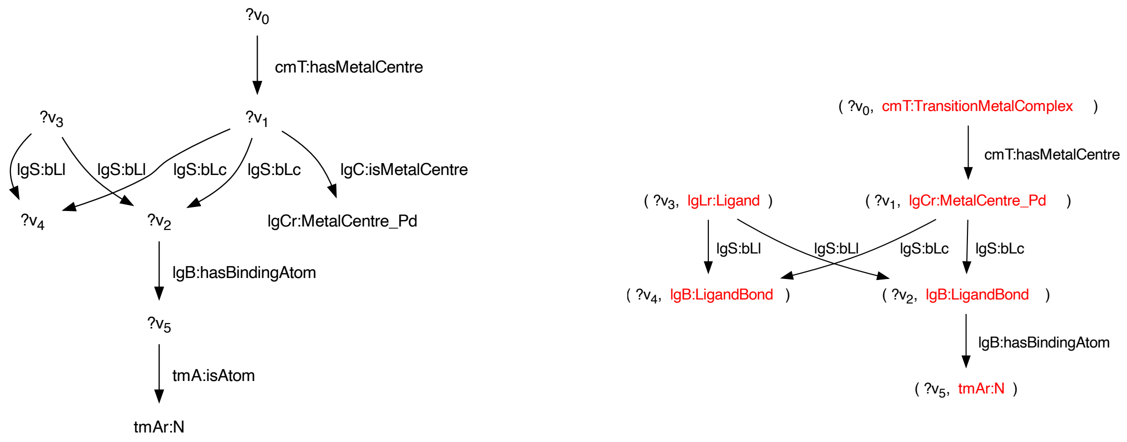

(a) (b)

Figure S12: (a) An example of a graph pattern. (b) Its corresponding compressed representation.

remaining nodes according to their most specific RDFS class. See Figure S12 for an example.

This new compressed representation enables the construction of a different kind of proxy for graph patterns. Consider the (logarithmically smoothed) tf-idf weighting scheme[17] defined as

$$\mathrm{tf}-\mathrm{idf}(\ell, p, \mathcal{P}) = \frac{N_{\ell,p}}{\sum_{\ell' \in p} N_{\ell',p}} \cdot \left(1 + \log \frac{|\mathcal{P}|}{1 + |\mathcal{P}_\ell|}\right) \tag{S30}$$

where ℓ is any of the labels appearing in the compressed representation $\tilde{p}$ of p, $N_{\ell,p}$ is the number of times that ℓ appears in $\tilde{p}$,[X] $\mathcal{P}$ is the set of frequent patterns and $\mathcal{P}_\ell$ is the set of (compressed) patterns in which ℓ appears at least once. Now, if $L_\mathcal{P}$ is the set of all the labels that appear in the graphs of $\{\tilde{p} \mid p \in \mathcal{P}\}$, then the feature vector is

$$\boldsymbol{z}_p := \big(\mathrm{tf}-\mathrm{idf}(\ell, p, \mathcal{P})\big)_{\ell \in L_\mathcal{P}} \tag{S31}$$

[X] In an RDF graph G, there exists a unique node (URI) representing an RDF class, and class assignment is performed via an edge that points to that URI. Hence, in compliance with the constraint imposing at most one node per label, there exists a unique instance of a class label in G. In the compressed representation, however, class assignment is performed via label assignment, hence there will be as many instances of a class as entities belonging to that class.

and the corresponding similarity metric becomes

$$s_{\cos;\,s}(p,q) := s_{\cos}(\boldsymbol{z}_p, \boldsymbol{z}_q). \tag{S32}$$

S9.2 Similarity Metric for DLGs

Another interesting metric is the similarity metric for DLGs introduced by Champin and Solnon (2003), hereafter denoted as s_{DLG}.[18] This is a metric that strongly relies on explicit label assignment, hence it naturally applies to the compressed representation introduced above.

Given two patterns (DLGs) p and q, and their compressed representations $\tilde{p} = (V_{\tilde{p}}, E_{\tilde{p}}, \pi_{\tilde{p}})$ and $\tilde{q} = (V_{\tilde{q}}, E_{\tilde{q}}, \pi_{\tilde{q}})$, s_{DLG} relies on a *multivalent mapping* $m \subseteq V_{\tilde{p}} \times V_{\tilde{q}}$, which maps every vertex in any of the two patterns with one or more (or even zero) nodes in the other. Following the original manuscript,[18] when two nodes $v \in V_{\tilde{p}}$ and $w \in V_{\tilde{q}}$ are mapped onto each other, we will denote it using the functional notation $m(v) = w$ or $m(w) = v$, depending on the context (but we consider the two expressions to be equivalent). Edges are mapped when their extremes are mapped. Each mapping m induces an "intersection" $\tilde{p} \sqcap_m \tilde{q}$ made of the nodes and edges that are mapped onto each other and that share the same label. By assigning a score to nodes, edges and their labels, and by eventually penalising features that are "split" (i.e., mapped onto two or more objects), it is possible to evaluate the entire intersection. Finally, the similarity score is defined as the evaluation of the intersection induced by best possible mapping m. Formally,

$$s_{\mathrm{DLG}}(p, q \mid \omega_{f,V}, \omega_{f,E}, \omega_g) = \max_{m \subseteq V_{\tilde{p}} \times V_{\tilde{q}}} \frac{f(\tilde{p} \sqcap_m \tilde{q} \mid \omega_{f,V}, \omega_{f,E}) - g(\mathrm{splits}(m) \mid \omega_G)}{f(\tilde{p} \cup \tilde{q} \mid \omega_{f,V}, \omega_{f,E})}, \tag{S33}$$

where

$$\tilde{p} \sqcap_m \tilde{q} := \bigcup_{\substack{\tilde{r},\,\tilde{s}\in\{\tilde{p},\tilde{q}\}\\ \tilde{r}\neq\tilde{s}}} \Big(\big\{ v \in V_{\tilde{r}} \mid \exists v' \in V_{\tilde{s}} :\, m(v) = v',\, \pi_{\tilde{r}}(v) = \pi_{\tilde{s}}(v') \big\} \tag{S34}$$

$$\cup\ \big\{ (v,w) \in E_{\tilde{r}} \mid \exists (v',w') \in E_{\tilde{s}} :\, m(v) = v', m(w) = w', \tag{S35}$$

$$\pi_{\tilde{r}}(v,w) = \pi_{\tilde{s}}(v',w') \big\} \Big), \tag{S36}$$

$$\text{splits}(m) := \bigcup_{\substack{\tilde{r},\,\tilde{s}\in\{\tilde{p},\tilde{q}\}\\ \tilde{r}\neq\tilde{s}}} \big\{ (v, s_v) \mid v \in V_{\tilde{r}},\, s_v = \{ v' \in V_{\tilde{s}} \mid m(v) = v' \},\, |s_v| \geq 2 \big\} \tag{S37}$$

$$\tilde{p} \cup \tilde{q} := V_{\tilde{p}} \cup E_{\tilde{p}} \cup V_{\tilde{q}} \cup E_{\tilde{q}} \tag{S38}$$

and

$$f(F \mid \omega_{f,V}, \omega_{f,E}) := \sum_{\tilde{r}\in\{\tilde{p},\tilde{q}\}} \left(\sum_{v \in F \cap V_{\tilde{r}}} \omega_{f,V}(v, \pi_{\tilde{r}}(v)) + \sum_{(v,w) \in F \cap E_{\tilde{r}}} \omega_{f,E}(v, w, \pi_{\tilde{r}}(v,w)) \right), \tag{S39}$$

$$g(S \mid \omega_g) := \sum_{(v,s_v)\in S} \omega_g(v, s_v). \tag{S40}$$

The choice of the weight functions $\omega_{f,V}, \omega_{f,E}$ and ω_g determine the behaviour of the similarity metric.

The optimisation problem in (S33) can be approached via greedy algorithm.[18] This solution, however, is non-deterministic, possibly suboptimal, and particularly expensive to compute, especially for large sets of graph patterns.

S9.2.1 Naive Weighting Scheme

Minimally informative weights can be chosen as

$$\omega_{f,V}^{\text{naive}}(v, \ell \mid \omega_0, \omega_1) = \begin{cases} \omega_0 & \text{if } \ell \in \{\texttt{lgCr:MetalCentre, lgLr:Ligand, tmA:Atom}\} \\ \omega_1 & \text{otherwise} \end{cases}, \tag{S41}$$

$$\omega_{f,E}^{\text{naive}}(v, w, \ell \mid \omega_2) = \omega_2, \tag{S42}$$

$$\omega_g(v, s_v) = |s_v|, \tag{S43}$$

for some non-negative constants $\omega_0, \omega_1, \omega_2$ such that $\omega_0 < \omega_1$. Notice that

$$\pi(v) \in \{\texttt{lgCr:MetalCentre}, \texttt{lgLr:Ligand}, \texttt{tmA:Atom}\} \tag{S44}$$

can only happen if v represents a metal centre, a ligand, or an atom and the pattern does not explicitly assign a class, meaning that the chemical identity of the node is left unspecified. We then define

$$s_{\text{DLG};n}(p, q \mid \omega_0, \omega_1, \omega_2) := s_{\text{DLG}}\left(p, q \mid \omega_{f,V}^{\text{naive}}(\cdot, \cdot \mid \omega_0, \omega_1), \omega_{f,E}^{\text{naive}}(\cdot, \cdot, \cdot \mid \omega_2), \omega_g\right). \tag{S45}$$

In this case, the constraint $\omega_0 < \omega_1$ has the effect of penalising pairs of patterns that specify contradictory chemical identities for the same entity versus pairs in which one pattern does not specify any identity (see Example S9.1).

Example S9.1. Consider the two compressed graph representations shown in Figures S13, panel (a) and S13, panel (b) and name them $\tilde{p}$ and $\tilde{q}$ respectively.

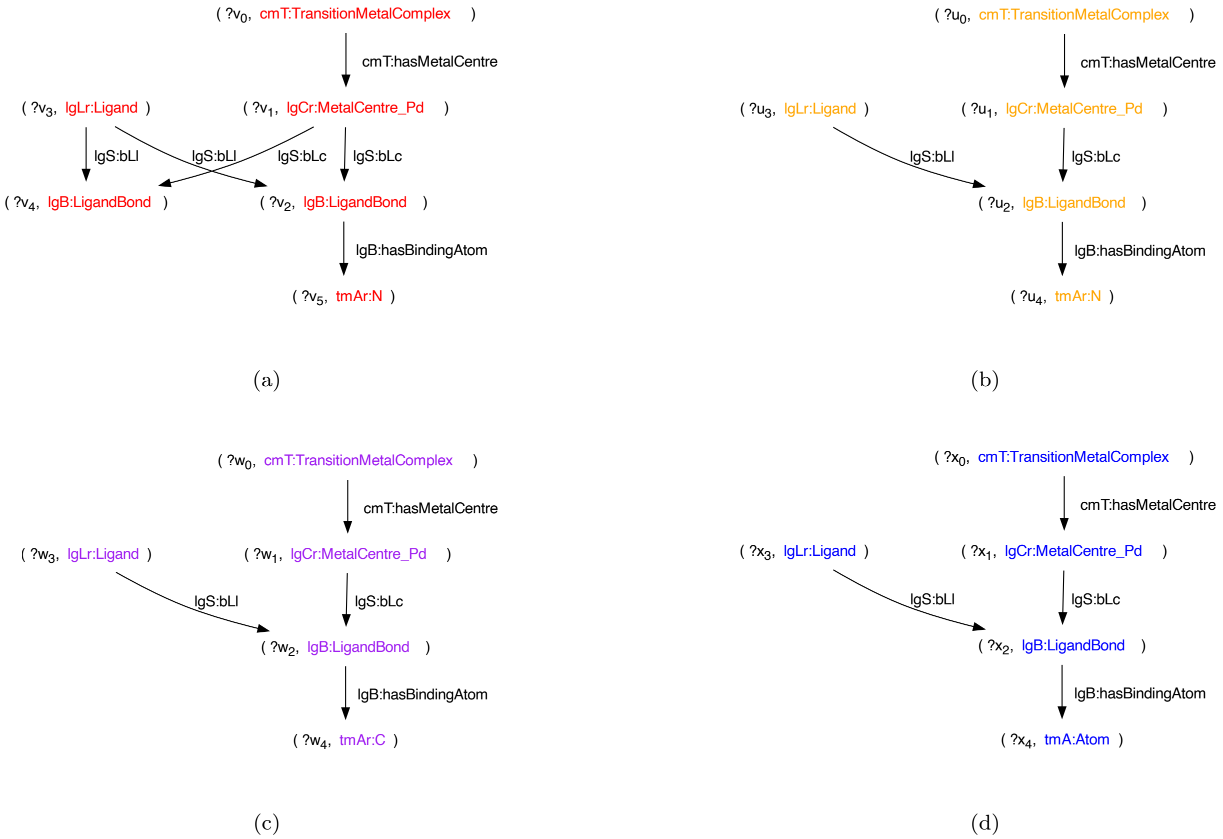

Figure S13: Four examples of compressed patterns. In Example S9.1, these are referred to as $\tilde{p}$, $\tilde{q}$, $\tilde{q}'$ and $\tilde{q}''$ respectively.

Consider then the following multivalent mapping:

$$v_0 \mapsto u_0 \tag{S46}$$

$$v_1 \mapsto u_1 \tag{S47}$$

$$v_2 \mapsto u_2 \tag{S48}$$

$$v_3 \mapsto u_3 \tag{S49}$$

$$v_4 \mapsto u_2 \tag{S50}$$

$$v_5 \mapsto u_4. \tag{S51}$$

This mapping essentially preserves the identity and role of each node and deals with the

different denticity of the specified ligand by collapsing the two bond objects found in $\tilde{p}$ onto the single bond object in $\tilde{q}$.

If we evaluate the objective function of (S33) in this scenario we find that

$$\tilde{p} \sqcap_m \tilde{q} = V_{\tilde{p}} \cup E_{\tilde{p}} \cup V_{\tilde{q}} \cup E_{\tilde{q}}, \tag{S52}$$

$$f(\tilde{p} \sqcap_m \tilde{q}) = \omega_1(|\tilde{p} \sqcap_m \tilde{q}| - 2) + 2\omega_0, \tag{S53}$$

$$\text{splits}(m) = \{(u_2, \{v_2, v_4\})\}, \tag{S54}$$

$$g(\text{splits}(m)) = 2, \tag{S55}$$

$$f(\tilde{p} \cup \tilde{q}) = \omega_1(|\tilde{p} \sqcap_m \tilde{q}| - 2) + 2\omega_0 \tag{S56}$$

where the second and the fourth equation are based on the fact that the intersection (via m) covers the entire union of the two patterns and all the nodes, except two, are assigned a specific chemical identity (when needed). All nodes and edges therefore receive the weight ω_1, except for the two unspecified nodes (the ligands) which receive the weight ω_0. For $\omega_0 = 0.5, \omega_1 = 1$, we get the candidate score

$$\frac{\omega_1(|\tilde{p} \sqcap_m \tilde{q}| - 2) + 2\omega_0 - 2}{\omega_1(|\tilde{p} \sqcap_m \tilde{q}| - 2) + 2\omega_0} = \frac{1 \cdot (21 - 2) + 2 \cdot 0.5 - 2}{1 \cdot (21 - 2) + 2 \cdot 0.5} = \frac{18}{20} = 0.9. \tag{S57}$$

A computational examination of the problem reveals also that the mapping hereby proposed is locally optimal.

Consider now a slightly different case, namely the case in which $\tilde{p}$ is compared first against the graph $\tilde{q}'$, represented in Figure S13, panel (c), and then against $\tilde{q}''$, Figure S13, panel (d). Both graphs are almost identical to $\tilde{q}$, except for the label of the nodes that correspond to u_4 in $\tilde{q}$, namely w_4 in $\tilde{q}'$ and x_4 in $\tilde{q}''$.

First, w_4 is labelled as `tmAr:C`. Under the same mapping used before (with u_i replaced by

w_i), now it holds that

$$\tilde{p} \sqcap_m \tilde{q}' = V_{\tilde{p}} \cup E_{\tilde{p}} \cup V_{\tilde{q}'} \cup E_{\tilde{q}'} \setminus \{v_5, u_4\} \tag{S58}$$

and the candidate score becomes

$$\frac{\omega_1(|\tilde{p} \sqcap_m \tilde{q}'| - 4) + 2\omega_0 - 2}{\omega_1(|\tilde{p} \sqcap_m \tilde{q}'| - 2) + 2\omega_0} = \frac{1 \cdot (21 - 4) + 2 \cdot 0.5 - 2}{1 \cdot (21 - 2) + 2 \cdot 0.5} = \frac{16}{20} = 0.8. \tag{S59}$$

Notice that the denominator did not change with respect to the previous case, as the label change did not alter the number of specified chemical identities.

Now, in $\tilde{q}''$, the node x_4 is labelled `tmA:Atom`. The intersection and the associated score are the same as before, but this time the denominator changes to

$$f(\tilde{p} \cup \tilde{q}'') = \omega_1(|\tilde{p} \sqcap_m \tilde{q}''| - 3) + 3\omega_0, \tag{S60}$$

as now there are three unspecified chemical identities. The similarity score in this case becomes

$$\frac{\omega_1(|\tilde{p} \sqcap_m \tilde{q}''| - 4) + 2\omega_0 - 2}{\omega_1(|\tilde{p} \sqcap_m \tilde{q}''| - 3) + 3\omega_0} = \frac{1 \cdot (21 - 4) + 2 \cdot 0.5 - 2}{1 \cdot (21 - 3) + 3 \cdot 0.5} = \frac{16}{19.5} = 0.82. \tag{S61}$$

This example then showcases the behaviour of the chosen weight functions, i.e., they penalise more two patterns which specify wrong identities with respect to two patterns in which one expresses uncertainty in terms of the identity, △

S9.2.2 Learned Semantic Weights

An alternative and more refined weighting scheme can however be devised. In particular, the weights should take two different principles into account. There is prior ontological information ready to be exploited: specific RDFS classes are more informative than general ones. In addition, there is empirical information that can be learned from the pattern dataset,

in the sense that the frequency of a label can be an indicator of its relevance (for instance, a rare ligand class may be more informative than a frequent one). These two principles can be accounted for by means of two separate mechanisms.

The ontological hierarchy of the labels can be defined by defining the weight of an identity-specifying label as the sum of two components:

$$\omega^{\text{learned}}(\ell) = \begin{cases} \omega_{\text{gen}}(\text{general}(\ell)) & \text{if general}(\ell) = \ell \\ \omega_{\text{gen}} + \omega_0(\ell) & \text{otherwise} \end{cases}, \tag{S62}$$

where ω_0 is an arbitrary (non negative) weighting function and general(ℓ) is the corresponding general-purpose RDFS class (e.g. if $\ell =$ `tmAr:C`, then general(ℓ) $=$ `tmAr:Atom`). By doing so, we can guarantee that specific labels will always have more influence than generic labels (recall the mechanism showcased in Example S9.1).

Now, empirical information can be incorporated by defining the weighting scheme ω_0 as a data-driven inverse document frequency weight:

$$\omega_{\text{gen}}(\ell) = 1 + \log \frac{|\mathcal{P}|}{1 + |\mathcal{P}_\ell|}, \tag{S63}$$

where, once again, $\mathcal{P}$ is the set of frequent patterns and $\mathcal{P}_\ell$ is the set of (compressed) patterns in which ℓ appears at least once. It is easy to see that $\omega_0(\ell)$ decreases as $|\mathcal{P}_\ell|$ increases.

Using these two weighting functions, we can define node and edge weighting functions

$$\omega_{f,V}^{\text{learned}}(v, \ell) = \omega^{\text{learned}}(\ell) \tag{S64}$$

$$\omega_{f,E}^{\text{learned}}(v, w, \ell) = \omega^{\text{learned}}(\ell), \tag{S65}$$

Table S7: The list of all the numerical parameters employed in this work, organised by topic, together with the value used. Unless otherwise specified, the same value of each parameter has been used across the experiments involving the two selection *earlyTM* and *lateTM*.

Parameter	Description	Reference	Value
		Dataset selections	
N_{seed}	Number of ligands in the seed	Section S3	*earlyTM:* 1350<br>*lateTM:* 350
		Frequent pattern mining and filtering	
α	Minimum number of matched graphs	Section S4.1.1	*earlyTM:* 10<br>*lateTM:* 20
p_0	Seed pattern	Section S4.1.1	$(?v_0,\, t_p,\, ?v_1)$, with<br>$t_p =$ `cmT:hasMetalCentre`
$M_{\max}$	Maximum pattern size	Section S5	12
$(\pi_i)_{i=2}^{M_{\max}}$	Rejection probabilities	Section S5	*earlyTM:* (0.1, 0.15, 0.2, 0.25 0.3, 0.4, 0.65, 0.72, 0.895, 0.9, 0.9)<br>*lateTM:* (0.2, 0.25, 0.25, 0.42 0.45, 0.45, 0.53, 0.8, 0.8, 0.798, 0.8)
$M_{\min}$	Minimum pattern size	Section S6	10
		Agglomerative clustering	
Δ	Set of candidate similarity thresholds	Section S4.3	$\left\{\frac{i}{200} \mid i = 0, \ldots, 200\right\}$
$M^{\mathcal{C}}_{\min}$	Minimum number of clusters	Section S4.3	300
$(\omega_0,\, \omega_1, \omega_2)$	Weights of $s_{\text{DLG};\,n}$	Section S9.2.1	(0.5, 1, 1)
		Bayesian Network	
λ_0	Dirichlet prior hyperparameters	Section S4.5.2	10

which finally allows us to define

$$s_{\text{DLG};\,l}(p, q \mid \omega^{\text{learned}}) := s_{\text{DLG}}(p, q \mid \omega_{f,V}^{\text{learned}}, \omega_{f,E}^{\text{learned}}, \omega_g) \tag{S66}$$

where $\omega_g(v, s_v) = |s_v|$, as in Section S9.2.1.

S10 Computational Parameters

We report in Table S7 the list of the values of all the parameters used in this work during the experiments described in in the main text and Section S4.

S11 Extensive Experimental Results

We have performed the experiment from Section 4 in the main text using all the 8 possible combinations of similarity metric and aggregation function to compute the feature matrix Y, as described in Section S4.

We briefly recall that the goal of the experiment was to learn a score function $f : \mathcal{G} \rightarrow [0, 1]$ which could be used as a means of evaluating the outcomes of elementary TMC manipulations. In particular, during the test phase we removed one random ligand from each TMC in the test population $\mathcal{G}_{\text{test}}$ and we then tried to reconstruct the original compound by computing all the possible reconstructions that can be formed using ligands from the training population. We then used f to rank all the possible reconstructions of each incomplete complex. The quality of the learned function f is assessed by measuring the top$-k$ accuracy, for $k \in \{1, 5, 10\}$, intended as the fraction of test TMCs that can be found among the top$-k$ highest scoring reconstructions that can be formed after one of their ligands is removed.

The full set of results is shown in Table S8.

S11.1 Discussion

As observed in the main document, the proposed BN-based scoring function performs differently in the two dataset selections. Furthermore, as seen in Table S8, this kind of behaviour can be observed in all the proposed configurations.

Although in both datasets our method consistently prevails over the baseline for $k = 10$, the two situations diverge at $k = 1$. In the *earlyTM* dataset, the frequency-based baseline always achieves the highest top-1 accuracy, whereas in the *lateTM* dataset we observe a somewhat more favourable situation, in the sense that our method is capable of surpassing the baseline, in most of the cases, already for $k = 1$.

These observations on a larger variety of possible BN and pattern-based scoring functions further supports the conclusion that the proposed approach, although not necessarily more

Table S8: Top−k accuracy measured during the TMC completion task for the two datasets employed and each of the 8 possible combinations of similarity metric and aggregation function. In each column, the highest and second-highest value are highlighted in boldface and italic respectively.

Metric	Variant	Aggregation	*No filter* Top−k accuracy k = 1	*No filter* k = 5	*No filter* k = 10	*Hapticity/denticity order* Top−k accuracy k = 1	*Hapticity/denticity order* k = 5	*Hapticity/denticity order* k = 10	*Charge* Top−k accuracy k = 1	*Charge* k = 5	*Charge* k = 10
					earlyTM						
Baseline	–	–	**0.462**	**0.691**	0.740	**0.540**	0.770	0.828	**0.632**	0.765	0.818
s_{cos}	$s_{cos;\,p}$	a_{max}	0.189	0.626	0.796	0.268	0.806	0.940	0.270	0.772	0.862
		a_{median}	0.200	0.647	0.794	0.264	*0.821*	**0.960**	0.286	0.779	0.845
	$s_{cos;\,s}$	a_{max}	0.172	0.618	0.784	0.244	0.789	**0.960**	0.250	0.762	0.847
		a_{median}	0.194	0.641	*0.806*	0.263	0.785	0.943	0.276	0.768	0.854
s_{DLG}	$s_{DLG;\,n}$	a_{max}	*0.226*	*0.664*	**0.816**	*0.307*	**0.836**	0.953	*0.312*	**0.796**	**0.872**
		a_{median}	0.184	0.623	0.798	0.245	0.804	*0.957*	0.259	0.772	*0.865*
	$s_{DLG;\,l}$	a_{max}	0.178	0.606	0.783	0.262	0.808	**0.960**	0.255	0.747	0.838
		a_{median}	0.182	0.640	0.798	0.250	0.799	0.953	0.270	*0.781*	0.840
					lateTM						
Baseline	–	–	0.153	0.303	0.381	0.200	0.404	0.531	0.205	0.402	0.534
s_{cos}	$s_{cos;\,p}$	a_{max}	*0.297*	**0.504**	*0.709*	*0.354*	**0.620**	*0.860*	**0.327**	**0.684**	*0.853*
		a_{median}	**0.305**	*0.497*	**0.725**	**0.355**	*0.600*	**0.877**	*0.319*	*0.672*	**0.860**
	$s_{cos;\,s}$	a_{max}	0.142	0.404	0.692	0.177	0.517	0.850	0.157	0.629	0.823
		a_{median}	0.162	0.420	0.689	0.200	0.534	0.850	0.193	0.628	0.826
s_{DLG}	$s_{DLG;\,n}$	a_{max}	0.260	0.443	0.688	0.316	0.553	0.830	0.286	0.629	0.826
		a_{median}	0.233	0.451	0.676	0.279	0.560	0.835	0.260	0.635	0.826
	$s_{DLG;\,l}$	a_{max}	0.207	0.430	0.662	0.251	0.535	0.822	0.221	0.611	0.812
		a_{median}	0.205	0.446	0.685	0.244	0.560	0.837	0.234	0.634	0.826

accurate than simple heuristics when dealing with frequently encountered ligands, is capable of modelling the behaviour of rarer ligands better than a elementary frequency-based analyses.

Focusing now only on the possible variations of our method, when comparing the different configurations of similarity metric, variant, and aggregation function, it emerges that no single setting can be considered as superior with respect to the others. At $k = 10$, i.e., once our method effectively surpasses the baseline performances, the maximum difference between the best and worst performing configurations across all filters and dataset is ~ 0.0630, attained in the *no filter* scenario within the *lateTM* dataset between the $(s_{\cos;p}, a_{\text{median}})$ and the $(s_{\text{DLG};l}, a_{\max})$ configurations.

Although this difference cannot be properly considered negligible, it is also not large enough to justify a performance-based distinction between the various configurations. This reason, paired with the intuitive interpretation of the $a_{\max}$ aggregation function and the relative simplicity of the $s_{\cos;p}$ metric, prompted us to choose this pair as the representative configuration whose results have been discussed in in the main document.

References

(1) Hogan, A. et al. Knowledge graphs. *ACM Comput. Surv.* **2021**, *54*, 71:1–71:37, DOI: 10.1145/3447772.

(2) Ell, B. Graph Pattern-based Association Rules Evaluated Under No-repeated-anything Semantics in the Graph Transactional Setting. DOI: 10.48550/arXiv.2512.15308, 2025.

(3) Hayes, P. RDF semantics. `https://www.w3.org/TR/2004/REC-rdf-mt-20040210/#rdfs_interp`, 2004; Accessed: 2025-06-12.

(4) Bergmann, M.; Moor, J.; Nelson, J. *The Logic Book*; McGraw-Hill/Connect Learn Succeed, 2014; ISBN: 978-0-07-803841-9.

(5) Klyne, G.; Carrol, J. J. Resource Description Framework (RDF): Concepts and Abstract Syntax. `https://www.w3.org/TR/2004/REC-rdf-concepts-20040210/#section-Graph-URIref`, 2004; Accessed: 2025-12-03.

(6) Brickley, D.; Guha, R. RDF schema 1.1. `https://www.w3.org/TR/rdf-schema/#ch_introduction`, 2014; Accessed: 2025-06-12.

(7) Ayers, D.; Völkel, M. Cool URIs for the semantic web. `https://www.w3.org/TR/cooluris/#oldweb`, 2008; Accessed: 2025-12-03.

(8) Beckett, D.; Berners-Lee, T.; Prud'hommeaux, E.; Carothers, G. RDF 1.1 turtle.

https://www.w3.org/TR/turtle/#grammar-production-prefixID, 2014; Accessed: 2025-12-03.

(9) De Giacomo, G.; Lenzerini, M. TBox and ABox reasoning in expressive description logics. Proceedings of the 1996 International Workshop on Description Logics, November 2-4, 1996, Cambridge, MA, USA. 1996; pp 37–48, https://aaai.org/papers/037-ws96-05-004/.

(10) Pascazio, L.; Rihm, S.; Naseri, A.; Mosbach, S.; Akroyd, J.; Kraft, M. Chemical species ontology for data integration and knowledge discovery. *Journal of Chemical Information and Modeling* **2023**, *63*, 6569–6586, DOI: 10.1021/acs.jcim.3c00820.

(11) Balasubramanian, V. N.; Ho, S.-S.; Vovk, V. *Conformal Prediction for Reliable Machine Learning: Theory, Adaptations and Applications*; Morgan Kaufmann, 2014; ISBN: 978-0-12-398537-8 978-0-12-401715-3.

(12) Harris, S.; Seaborne, A. SPARQL 1.1 query language. https://www.w3.org/TR/2013/REC-sparql11-query-20130321/, 2013; Accessed: 2025-11-18.

(13) Legendre, P. Cluster analysis. Developments in Environmental Modelling. 2012; pp 337–424, DOI: 10.1016/B978-0-444-53868-0.50008-3.

(14) Koller, D.; Friedman, N. *Probabilistic Graphical Models: Principles and Techniques*; MIT Press, 2009; ISBN: 978-0-262-01319-2.

(15) Kitson, N. K.; Constantinou, A. C.; Guo, Z.; Liu, Y.; Chobtham, K. A survey of Bayesian network structure learning. *Artificial Intelligence Review* **2023**, *56*, 8721–8814, DOI: 10.1007/s10462-022-10351-w.

(16) Lai, H.; Huang, T.; Lu, B.; Zhang, S.; Xiaog, R. Silhouette coefficient-based weighting k-means algorithm. *Neural Computing and Applications* **2025**, *37*, 3061–3075, DOI: 10.1007/s00521-024-10706-0.

(17) Salton, G.; Buckley, C. Term-weighting approaches in automatic text retrieval. *Information Processing & Management* **1988**, *24*, 513–523, DOI: 10.1016/0306-4573(88)90021-0.

(18) Champin, P.-A.; Solnon, C. Measuring the similarity of labeled graphs. 5th Int. Conf. On Case-Based Reasoning (ICCBR 2003). 2003; pp 80–95, DOI: 10.1007/3-540-45006-8_9.

(19) Sorlin, S.; Solnon, C. Reactive tabu search for measuring graph similarity. Graph-Based Representations in Pattern Recognition. 2005; pp 172–182, DOI: 10.1007/978-3-540-31988-7_16.

(20) Jiang, X.; Munger, A.; Bunke, H. On median graphs: properties, algorithms, and applications. *IEEE Transactions on Pattern Analysis and Machine Intelligence* **2001**, *23*, 1144–1151, DOI: 10.1109/34.954604.

(21) Johnstone, I. M.; Titterington, D. M. Statistical challenges of high-dimensional data. *Philosophical Transactions of the Royal Society A: Mathematical, Physical and Engineering Sciences* **2009**, *367*, 4237–4253, DOI: 10.1098/rsta.2009.0159.

(22) Scutari, M. Learning Bayesian Networks with the bnlearn R package. *Journal of Statistical Software* **2010**, *35*, 1–22, DOI: 10.18637/jss.v035.i03.

(23) Ankan, A.; Textor, J. pgmpy: a Python toolkit for Bayesian networks. *Journal of Machine Learning Research* **2024**, *25*, 1–8, `http://jmlr.org/papers/v25/23-0487.html`.

(24) Boettiger, C.; Mecum, B. virtuoso: Interface to 'Virtuoso' using 'ODBC'. `https://CRAN.R-project.org/package=virtuoso`, 2021.
